# Development of applicable thiol-linked antibody–drug conjugates with improved stability and therapeutic index

**DOI:** 10.1080/10717544.2022.2039807

**Published:** 2022-03-04

**Authors:** Yanming Wang, Fei Xie, Lianqi Liu, Xin Xu, Shiyong Fan, Wu Zhong, Xinbo Zhou

**Affiliations:** National Engineering Research Center for the Emergency Drug, Beijing Institute of Pharmacology and Toxicology, Beijing, China

**Keywords:** ADC, linker, maleimide, maleamic methyl ester, stability

## Abstract

Maleimides are typically applicable for coupling with reactive thiol moieties of antibodies in antibody–drug conjugates (ADCs) via the thiol-Michael click chemistry. Even so, the thiosuccinimide group produced in ADCs is unstable under physiological conditions, which is a unresolved issue in the ADC industry that can cause serious off-target toxicity. Committed to solving the stability defects of traditional thiosuccinimide-containing ADCs, we explored a series of linkers based on the ring-opening hydrolysates of thiosuccinimide. Meanwhile, a type of linkers based on maleamic methyl ester were used to conjugate the popular monomethyl auristatin E to an anti-HER2 antibody to generate the target ADCs, which enhances the stability and do not need to change the structure of the ideal stable metabolite of traditional ADCs. *In vivo* studies demonstrate that our preferred ADC mil40-**12b** not only has better efficacy than traditional ADCs but also exhibits better safety parameters in mice. For example, complete tumor regression can still be achieved even when the dose is halved (2.5 mg/kg), and the maximum tolerable dose is increased by 40 mg/kg. This strategy is expected to provide an applicable tool for the construction of thiol-linked ADCs with improved therapeutic index.

## 1. Introduction

Antibody–drug conjugates (ADCs), which combine the selectivity of antibodies with the potency of cytotoxins, represent a rapidly increasing field in cancer therapy (Peters & Brown, 2015; Pryyma et al., 2020; Zhu et al., 2020; Criscitiello et al., 2021). To date, there are 12 FDA-approved ADCs available to patients (Drago et al., 2021; Walsh et al., 2021), and over 80 ADCs have been investigated in different stages from approximately 600 clinical trials (Joubert et al., 2020; Zhao et al., 2020; Alas et al., 2021).

**Figure 1.**
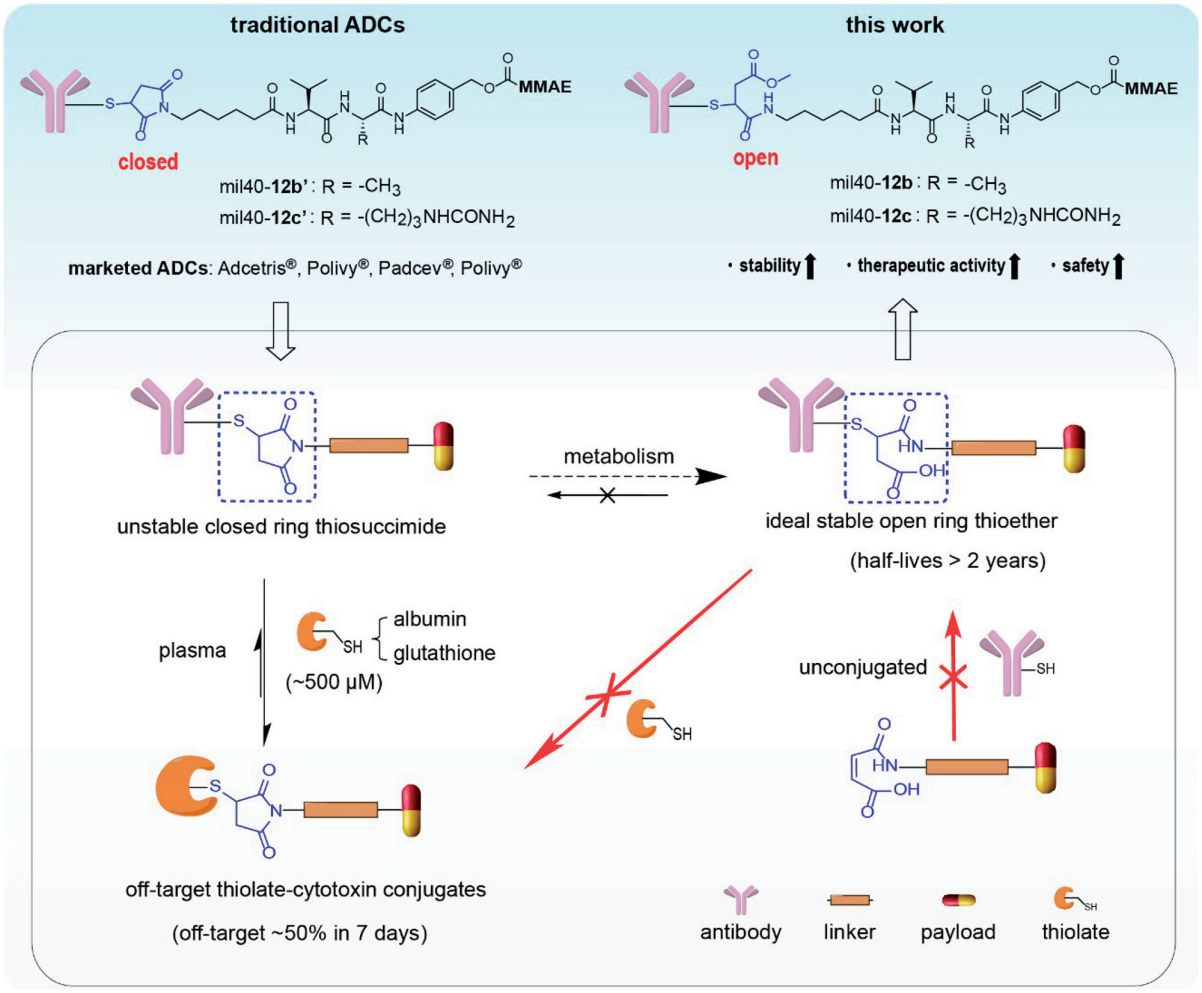
The two different metabolic pathways of traditional thiosuccinimide-containing ADCs in the circulation system and the structure of the designed ADCs based on maleamic methyl ester in this work. ADCs: antibody–drug conjugates; MMAE: monomethyl auristatin E.

Maleimides are widely applicable for coupling with the reactive thiol groups of antibodies via the Michael addition reactions due to their high selectivity, fast reaction kinetics, and mild reaction conditions (Kim et al., 2008; Henkel et al., 2016; Huang et al., 2019; Ravasco et al., 2019). For example, 10 (Adcetris^®^, Kadcyla^®^, Polivy^®^, Lumoxiti^®^, Padcev^®^, Enhertu^®^, Trodelvy^®^, Blenrep^®^, Zynlonta^®^, and Tivdak^®^) of the 12 FDA-approved ADCs use maleimide–thiol reactions during their construction (Walsh et al., 2021). Even so, the thiosuccinimide linkage produced in these ADCs is unstable in the presence of thiol-containing substances, because it can be eliminated by retro-Michael reactions or exchange with endogenous thiols such as albumin and glutathione (Figure 1) (Lyon et al., 2014; Huang et al., 2019), resulting in suboptimal pharmacodynamic, pharmacokinetic, and safety profiles. Reportedly, the shedding rate of payloads from thiosuccinimide-containing ADCs in plasma can be as high as 50–75% within 7–14 days (Alley et al., 2008; Lyon et al., 2014).

The ADC industry has made great efforts to solve the insufficient stability of thiosuccinimide-containing ADCs (Ravasco et al., 2019). Among these efforts, the most valuable discovery is that thiosuccinimide can be partially metabolized into a stable open ring form under physiological conditions, which is resistant to elimination of the thiosuccinimide bond (*t*_1/2_ >2 years) (Tumey et al., 2014; Fontaine et al., 2015). The direct preparation of ‘ring-opening’ product has always been the goal pursued by scientists in ADC industry, unfortunately, it is difficult to achieve due to the weak reactivity of the maleic acid amide group (Knight, 1979; Ryan et al., 2011). The reported methods for the preparation of stable ring-opening products mainly utilize the maleimide–thiol reactions to generate thiosuccinimide and then induce its hydrolysis (Shen et al., 2012; Lyon et al., 2014; Tumey et al., 2014). However, these circuitous strategies usually require an introduction of additional catalysis groups adjacent to maleimide, or hydrolyze thiosuccinimide-containing ADCs in a specific alkaline buffer, resulting in additional clinical verification, and increased risk of contamination and denaturation of the final product. To date, there is still high demand for tuning the chemistry of thiol-linked ADCs.

In this study, we reported an applicable linker technology to directly generate ideal thiol-linked ADCs based on the ring-opening hydrolysate of thiosuccinimide, which address the issue of weak addition reactivity between the maleic acid amide and the thiol groups. For the first time, a novel type of thiol-reactive linkers based on maleamic methyl ester were used to conjugate the popular monomethyl auristatin E (MMAE) to a humanized anti-HER2 antibody to generate the target ADCs (Figure 1). Our tests indicated that the preferred ADC mil40-**12b** not only has better stability and *in vivo* efficacy than traditional ADCs, but also exhibits better safety parameters, such as body weight, hematology, histopathology, and maximum tolerated dose (MTD). For example, compared with traditional thiosuccinimide-containing ADCs, mil40-**12b** can still achieve complete tumor regression even when the dose was halved (2.5 mg/kg), and its MTD is increased by 40 mg/kg. This strategy is expected to provide an applicable and universal tool for the construction of thiol-based traditional ADCs and a new generation of site-specific ADCs.

## 2. Materials and methods

### 2.1. Synthesis information

For details, see Supporting Information.

### 2.2. Preparation of the antibody–drug conjugates

The maleamic methyl ester-based ADCs were generated by previously described methods (Wang et al., 2017, 2019, 2020). Briefly, humanized antibody mil40 (a biosimilar of Herceptin^®^, Hisun Pharmaceutical Co., Ltd., Zhejiang, China) in l-histidine buffer (pH 7.5) was conjugated to maleamic methyl ester-containing linker-MMAE conjugates (8 or 16 equivalents/antibody) for 2–4 h at 25 °C. After quenched the reactions with *N*-acetyl-l-cysteine (NAC), the reaction solution was buffer-exchanged for elution by Sephadex G25 size-exclusion chromatography.

### 2.3. *In vitro* stability with overexposed thiols

ADCs (2 mg/mL, drug-to-antibody ratio (DAR) ≈ 8) based on the traditional maleimide and the maleamic methyl ester were incubated in PBS at 37 °C, and excess thiol substance (NAC, 100 equivalents/antibody) was added to each solution. Aliquots (250 μL; *n* = 3/group) were sampled at each predetermined time points (0, 1, 2, 3, 6, 9, 14, and 21 days) and frozen at −80 °C. After completion of sampling, melted the samples, which were analyzed by HPLC-HIC. Differences in the stabilities of the two ADCs were characterized using the changes in the DAR values over time.

Analogously, the above two ADCs (∼3.4 mg/mL) were incubated in PBS, and an equal volume of albumin in PBS solution (50 mg/mL) was added followed by incubation at 37 °C. Aliquots (250 μL; *n* = 3/group) were sampled at each predetermined time points (0, 6, 12, 24, 36, 48, 72, 168, and 336 h) and frozen at −80 °C. After completion of sampling, all samples were tested according to the above method to detect the proportion of payload shedded from the detected ADCs.

### 2.4. *In vitro* cytotoxicity

HER2^+^ tumor cell lines (SK-BR-3, BT-474, SK-OV-3, and NCI-N87) and HER2^–^ tumor cell lines (MCF-7 and MDA-MB-468) were purchased from the ATCC (Manassas, VA). BT-474 cells and MCF-7 cells were cultured in Dulbecco’s modified Eagle medium (DMEM) (Gibco, Carlsbad, CA), SK-BR-3 cells and SK-OV-3 cells were cultured in McCoy's 5A (Gibco, Carlsbad, CA), NCI-N87 cells were cultured in RPMI-1640 (Gibco, Carlsbad, CA), and MDA-MB-468 were cultured in L-15 (Gibco, Carlsbad, CA), which were all supplemented with 10% heat inactivated fetal bovine serum (Gibco, Carlsbad, CA). Each group was established three holes, tumor cells (3 × 10^4^ cells/mL) were added to each well of plate after which 10 μL of test compounds solution was added. The plates were incubated for seven days at 37 °C, then, incubated at RT. Cell Titer Glo^®^ reagent (40 μL) was added to each well, and incubated the plates for another 30 min. The luminescence of each well was detected using an EnSpire Plate Reader. GraphPad Prism software (Version 8.0.2; La Jolla, CA) was used for the calculation of IC_50_ values.

### 2.5. Colocalization test of cell trafficking

BT-474 cells were incubated for 24 h at 37 °C under 5% CO_2_. After removal of the medium, the cells were treated with FITC-labeled mil40, or FITC-labeled ADC in medium at 4 °C for 30 min, or the test substance was washed off followed by treatment at 37 °C for 24 h. Groups incubated at 4 °C were used to observe the affinity of the naked antibody or ADC to BT-474 cells, and the groups incubated at 37 °C were used to observe internalization. Upon reaching the time endpoints, the BT-474 cells were washed and the lysosomes were stained with LysoTracker and the nuclei were stained with DAPI. Fluorescent images were captured under a fluorescence microscope.

### 2.6. Cell cycle arrest and apoptosis analysis

BT-474 and NCI-N87 tumor cells were seeded (1 × 10^5^ cells/well) and exposed to ADC at different concentrations (0, 10, and 100 μg/mL) for 24 h. Cell apoptosis was detected using propidium iodide and Annexin V-FITC and stained with a FACSCalibur instrument (Becton Dickinson, Franklin Lakes, NJ). Cell cycle arrest was measured using incorporate bromodeoxyuridine, anti-BrdUrd FITC, and PI with the above-mentioned FACSCalibur instrument.

### 2.7. *In vivo* activity in xenograft tumors

In the human breast xenograft tumor model, 6–8-week-old female NOD/SCID mice (∼22 g) were subcutaneously inoculated with approximately 1 × 10^7^ BT-474 cells. When the tumor size reached ∼150 mm^3^, test mice (*n* = 6/group) were given ADC (1, 2.5, or 5 mg/kg), antibody + MMAE, or physiological saline via the tail veins at each predetermined time points (0, 7, 14, and 21 day). The animal weights and tumor sizes were monitored twice weekly. Tumor volume was calculated using the formula: TV=*a*×*b*^2^/2 (*a*: long tumor diameter; *b*: short tumor diameter). Animal experiments were performed following the protocol approved by the Institutional Animal Care and Use Committee at Pharmaron Co., Ltd. (license number: ON-CELL-XEM-06012016).

### 2.8. Safety at the therapeutic dose

Mice in the BT-474 xenograft test were executed to hematological analysis on days 28 and 58. In short, 100 μL of whole blood from each test mice in the 2.5 mg/kg ADC therapy group was drawn (*n* = 6/group), and certain samples were diluted threefold to determine the final complete blood count due to volume limitations. Hematology parameters were analyzed according to the values determined with a corrected dilution factor.

Test mice in specific groups (2.5 mg/kg) were also used for histopathological studies on day 58. Tissue samples of liver, heart, and lung were fixed in 10% buffered formalin for 24 h, and then transferred to 70% ethanol. Dehydration was performed with different concentrations of ethanol (85%, 90%, 95%, and 100%) and followed with xylene and paraffin. The above tissues were sectioned after embedding them in paraffin blocks, and then, H&E staining, and photographing were performed under a microscope.

### 2.9. Acute toxicity study

Seven- to nine-week-old CD-1 mice (20–30 g), purchased from Beijing Vital River Lab Animal Technology Co., Ltd. (Beijing, China), were dosed via the tail veins with 20 mg/kg ADCs, a mixture of mil40 and MMAE (equivalent dose or volume to ADC), or vehicle (physiological saline); each test group consisted of nine female mice (*n* = 9/group). After administration, all test animals were monitored for body weight changes once a day. Three mice were randomly selected from each group (*n* = 3/group) on days 4, 7, and 14 for sacrifice and whole blood hematology analysis. For the hematology analysis, ∼500 μL of whole blood was drawn from each animal.

During the abovementioned test, half of the test mice were euthanized for histopathological testing on day 7. The tissue samples (liver and lung) were subjected to paraffin embedding, sectioning, and H&E staining (*n* = 3/group). Similarly, the femur bones of the test animals were taken to create two bone marrow smears, and the bone marrow smears were air-dried and fixed in methanol and retained for observation under the microscope together with the histopathological sections after the trial.

### 2.10. High-dose tolerance study

Seven- to nine-week-old CD-1 female mice (20–35 g) were dosed with ADCs (10, 20, 40, 80, and 120 mg/kg) via the tail veins, and each test group consisted of three mice. The body weight changes of the animals were monitored once a day, meanwhile, their side reactions and behaviors were observed twice a day. After completion of the observation (14 days), all animals were euthanized by inhalation of ∼100% CO_2_. The criteria for determining MTD in this test were the death of the experimental animal, the loss of more than 20% of the animal’s body weight, or the occurrence of obviously intolerable adverse reactions.

### 2.11. Statistical analysis

Data were expressed as the means ± SD. Unpaired two-tailed *t* test was used to statistical analysis of the indicators, and the level of statistical significance was set at *p* < .05. Statistical analyses were performed using GraphPad software (Version 8.0.2, La Jolla, CA).

## 3. Results

### 3.1. Synthesis of the maleamic methyl ester-based conjugates and ADCs

To develop a novel type of thiol-linked linker system, we designed a variety of thiol-reactive maleimide analogs based on maleic acid amide, fumaric acid amide, fumaramide methyl ester, and maleamic methyl ester groups in order to develop a novel type of thiol-linked linker system for ADCs. Initially, we synthesized two linker-MMAE conjugates (**S5** and **S6**) with a thiol-reactive maleic acid amide (Supporting Information, Scheme S1). During the preparation of the corresponding ADCs, we found that even if the reaction time was extended by 10-fold, only a very small amount (DAR <0.5) of antibody modification could be achieved (Supporting Information, Figure S1). Moreover, we tried to react fumaric acid amide with the reduced antibody, but the desired effect was also not achieved (data not shown). We speculate that the hydrolysis of the maleimide ring to maleic acid changed the ring tension in the original structure. In addition, the generated carboxyl group may significantly reduce the electron cloud density of the double bond or form a hydrogen bond with the thiol (Knight, 1979; Ryan et al., 2011), which is not conducive to the reaction with the thiol groups of the antibody.

Further, we synthesized two thiol-reactive linker-MMAE conjugates (**S12** and **S13**) with thiol-reactive fumaramide methyl ester (Supporting Information, Scheme S2) (Kamimura et al., 2002; Jha et al., 2010). We hoped that the carboxyl group generated by the hydrolysis of the esterified ring can supplement the loss in electron cloud density of the double bond to a certain extent and overcome the hindrance of the coupling process by avoiding hydrogen bonding. However, the results of the antibody coupling experiments showed that this type of linker also cannot effectively couple the sulfhydryl group of antibody (Supporting Information, Figure S2).

**Figure 2.**
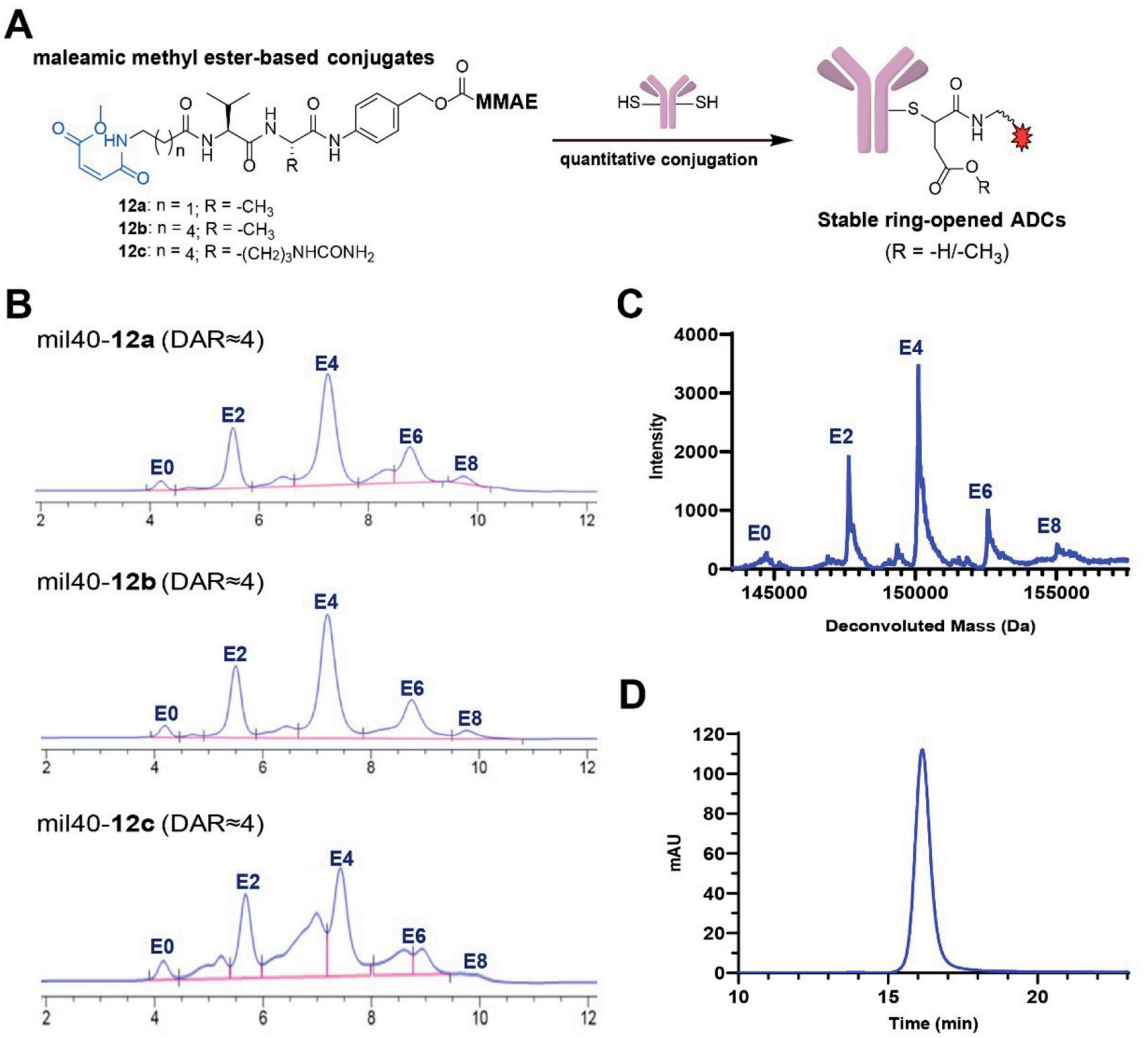
(A) The process of generating ADCs based on a thiol-reactive maleamic methyl ester group. (B) HIC analysis of the novel ADCs with maleamic methyl ester-based linkers. (C) UPLC-Q-TOF-MS analysis of mil40-**12b**. (D) Aggregation of mil40-**12b** that analyzed by size exclusion chromatography. DAR: drug-to-antibody ratio.

Finally, we tried linker-MMAE conjugates (**12a**, **12b**, and **12c**) based on the thiol-reactive maleamic methyl ester group (Supporting Information, Scheme S3) (Yi et al., 2009; Cao & Xi, 2013; Liu et al., 2017). Fortunately, the subsequent antibody coupling reaction showed that the linkers based on the maleamic methyl ester joint have similar reaction characteristics to traditional maleimide and can effectively couple with the thiol groups of antibody (Figure 2(A)). We used the selected maleamic methyl ester-based joint to prepare thiol-reactive model molecules, and tried to carry out Michael addition reactions with various types of thiols (Supporting Information, Scheme S4). The results showed that under mild reaction conditions, the maleamic methyl ester group can undergo addition reactions with different types of mercaptans, thereby directly obtaining a ring-opening product with a clear structure. It is therefore expected that a maleamic methyl ester-based linker system could be developed to directly obtain ring-opening thiol-linked ADCs through a one-step coupling reaction while maintaining the inherent advantages of maleimide-based traditional ADCs.

According to our previous methods (Wang et al., 2019, 2020), a novel type of maleamic methyl ester-based linkers were used to conjugate the popular MMAE to an anti-HER2 antibody mil40 to generate the target ADCs. HPLC-hydrophobic interaction chromatography (HIC) spectra showed that the average DAR of the maleamic methyl ester-based ADCs was approximately 4 (Figure 2(B)), with the drug distribution of mil40-**12b** appearing to be optimal. In addition, we used Q-TOF-MS to further conduct drug loading distribution studies on mil40-**12b**, which had the best drug distribution by HIC analysis. The results showed that the drug distribution obtained by Q-TOF-MS and HPLC-HIC analyses showed a basically consistent drug distribution (Figure 2(C)), and the calculated DAR values were essentially the same (3.80 and 3.91, respectively). In addition, we further analyzed the degree of aggregation of mil40-**12b**, and the results demonstrated that there was no significant difference between the naked antibody mil40 and ADC mil40-**12b**, where the proportion of protein monomers was >99% (Figure 2(D)). The above results showed that the maleamic methyl ester-based linker system has the desired thiol-coupling reaction performance and potential application value.

### 3.2. Conjugate stability assays

**Figure 3.**
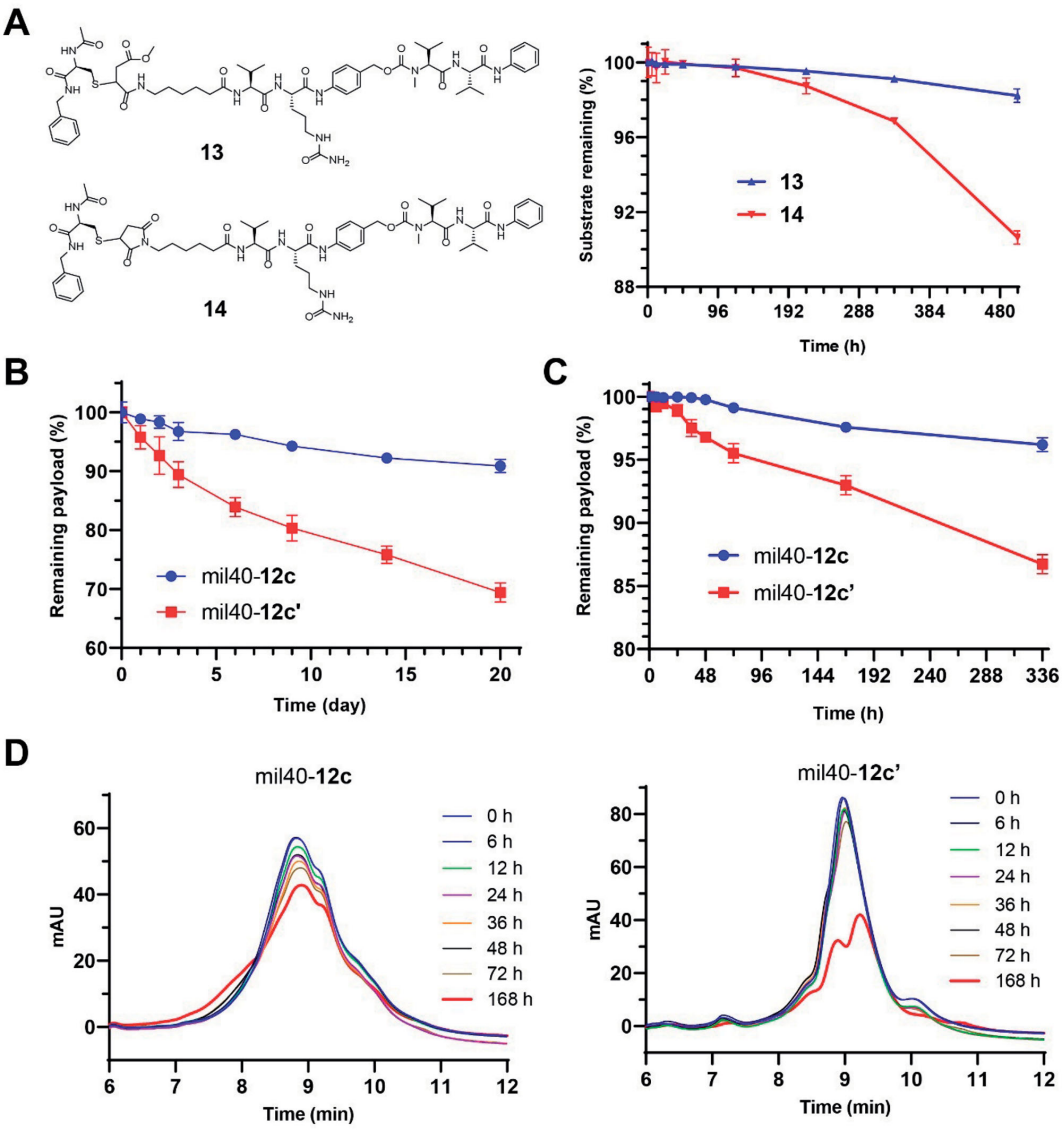
Stability studies of the maleamic methyl ester-based conjugates and ADCs. (A) The structures and stabilities of the model molecules in the presence of GSH. The results are shown as the means ± SD (*n* = 3/group). (B, C) The stability of the maleamic methyl ester-based and maleimide-based ADCs in the presence of NAC and albumin, respectively. The results are shown as the means ± SD (*n* = 3/group). (D) Changes in HIC data of the maleamic methyl ester-based and maleimide-based ADCs over time.

In order to verify the stability of this maleamic methyl ester-based linker system, we first prepared a maleamic methyl ester-based model small molecule (compound **13**; Supporting Information, Scheme S5) that is easier to prepare and detect, and prepared the maleimide-based traditional molecule (compound **14**; Supporting Information, Scheme S5) as a control. As shown in Figure 3(A), in the presence of an excess of the capture agent GSH (100 equiv.), compound **13** was more stable than compound **14** during 21 days of incubation at 37 °C (*p* < .0001), and the substrate loss of the two compounds was 1.8% and 10%, respectively. These results preliminarily show that the development of this novel thio-linked coupling technology based on maleamic methyl ester is expected to overcome the stability problem of the traditional maleimide–thiol coupling system.

To verify the stability advantages of the resulting maleamic methyl ester-based ADCs, ADC mil40-**12c** was evaluated for its *in vitro* stability in the presence of excess thiol (NAC), which was used as a capture agent for linker–cytotoxin conjugates shedded from ADCs. To increase discrimination, a high loading rate mil40-**12c** (DAR ≈8) in PBS was tested, and simultaneous use of the corresponding ADC (mil40-**12c′**; mil40-mc-VC-PABC-MMAE) based on a traditional maleimide linker was used as a control (Wang et al., 2017). After incubation at 37 °C for 21 days, mil40-**12c** showed only a small amount of shedded payload (∼9%) (Figure 3(B)). As a comparison, the shedding rate of the linker–cytotoxin conjugates from mil40-**12c′** reached as high as 31%. The trend of DAR over time for these two ADCs indicates that the maleamic methyl ester-based ADC has significantly improved stability compared with the conventional maleimide-based ADCs (*p* < .01) in the presence of excess reducing thiol groups.

Furthermore, we used an albumin solution to further evaluate the stability of the ADC based on a maleamic methyl ester linker in a physiological environment. As shown in Figure 3(C), for the maleamic methyl ester-based ADC mil40-**12c**, only approximately about 3.8% of the payload was shedded in the albumin solution (25 mg/mL) after 14 days of incubation at 37 °C. As a control, the shedded payload of the traditional ADC mil40-**12c′** was as high as 13.3% (Figure 3(D)). At the end of sampling, we also performed LC–MS detection on the two samples, but only the molecular weight corresponding to albumin-**12c′** was detected (QTOF-MS *m/z*: 67755.3 Da, [M + H]^+^). The test results also showed that the maleamic methyl ester-based ADC had a stability advantage over the traditional maleimide-based ADC (*p* < .0001), which is expected to significantly improve the common off-target cytotoxicity problem of the current thio-linked ADCs.

### 3.3. *In vitro* cytotoxicity

**Figure 4.**
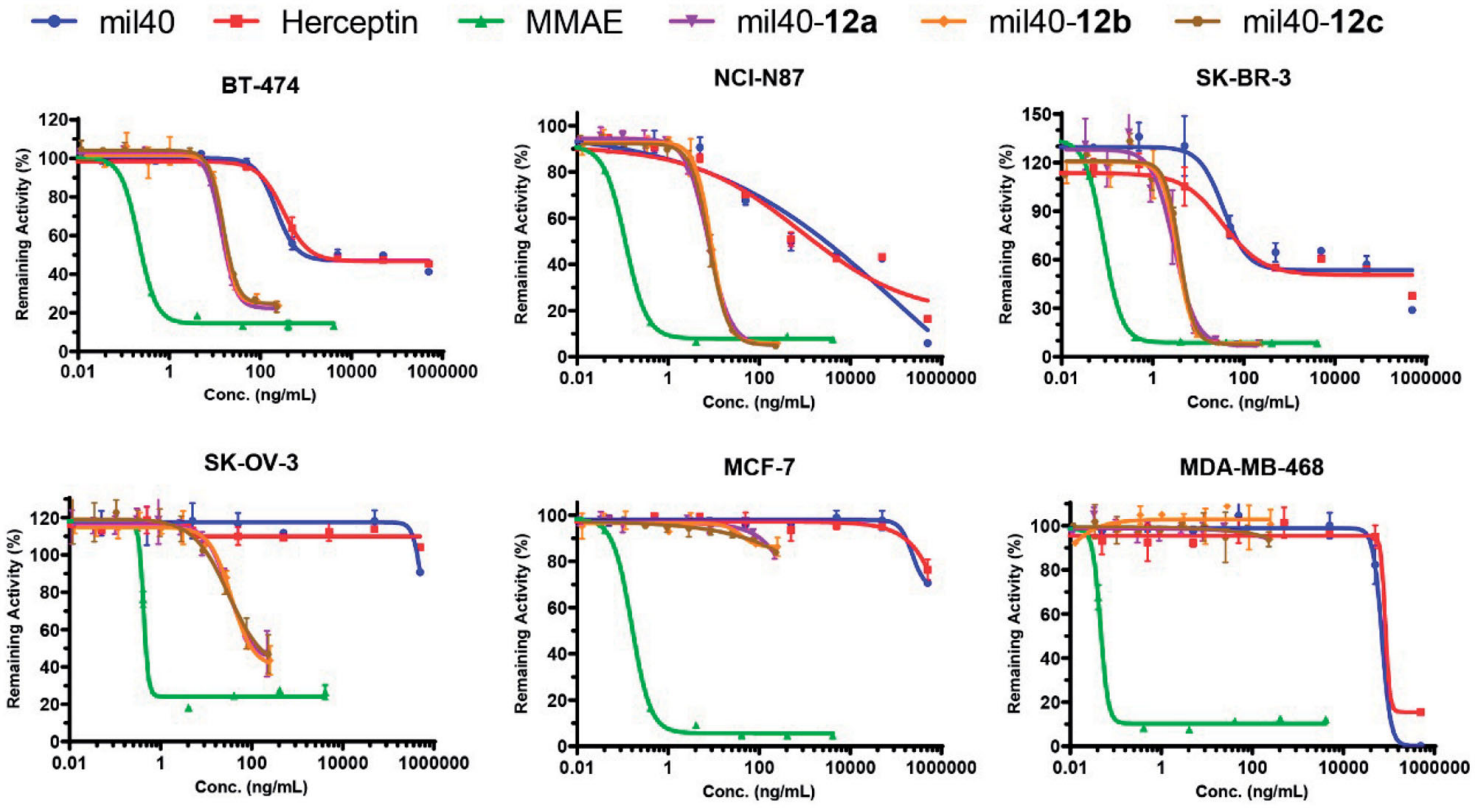
*In vitro* cytotoxicity test of the maleamic methyl ester-based ADCs. The tumor cell lines BT-474, SK-OV-3, SK-BR-3, and NCI-N87 are HER2^+^, and the tumor cell lines MCF-7 and MDA-MB-468 are HER2^–^. Three wells were established for each group, and the results are shown as the means ± SD (*n* = 3/group).

To evaluate the cytotoxicity, HER2^+^ tumor cell lines (SK-BR-3, BT-474, SK-OV-3, and NCI-N87), and HER2^–^ tumor cell lines (MCF-7 and MDA-MB-468) were treated with three generated maleamic methyl ester-based ADCs. As shown in Figure 4, these three ADCs exhibited more potent activity than antibodies in the HER2^+^ cell line according to both their IC_50_ values and inhibition, with the selectivity between HER2^+^ and HER2^–^ cell lines increasing by approximately 1000-fold. As a comparison, their payload MMAE exhibited potent antitumor activity in both HER2-positive and HER2-negative cell lines. In addition, these ADCs also displayed significant cytotoxicity against various tumor cells expressing HER2 with IC_50_ values ranging from 0.02 to 0.2 nM (Table 1), which is consistent with maleimide-based ADCs, as previously reported (Wang et al., 2017).

**Table 1.** *In vitro* cytotoxicity of the antibodies, MMAE and ADCs.

Cell lines	Test compound (IC_50_, nM)					
	Herceptin	mil40	MMAE	mil40-**12a**	mil40-**12b**	mil40-**12c**
BT-474	2.2192	1.5406	0.2986	0.0937	0.1026	0.1038
NCI-N87	0.5447	0.3599	0.1611	0.0513	0.0536	0.0587
SK-BR-3	0.2425	0.2739	0.1164	0.0201	0.0258	0.0232
Sk-OV-3	>2703	2762.6134	0.6444	0.2151	0.2074	0.2372
MCF-7	>2703	3009.3732	0.4028	23.3137	26.4386	20.0725
MDA-MB-468	1233.4115	917.1025	0.1043	24.1351	11.5147	12.7128

### 3.4. Fluorescence microscopy colocalization test

**Figure 5.**
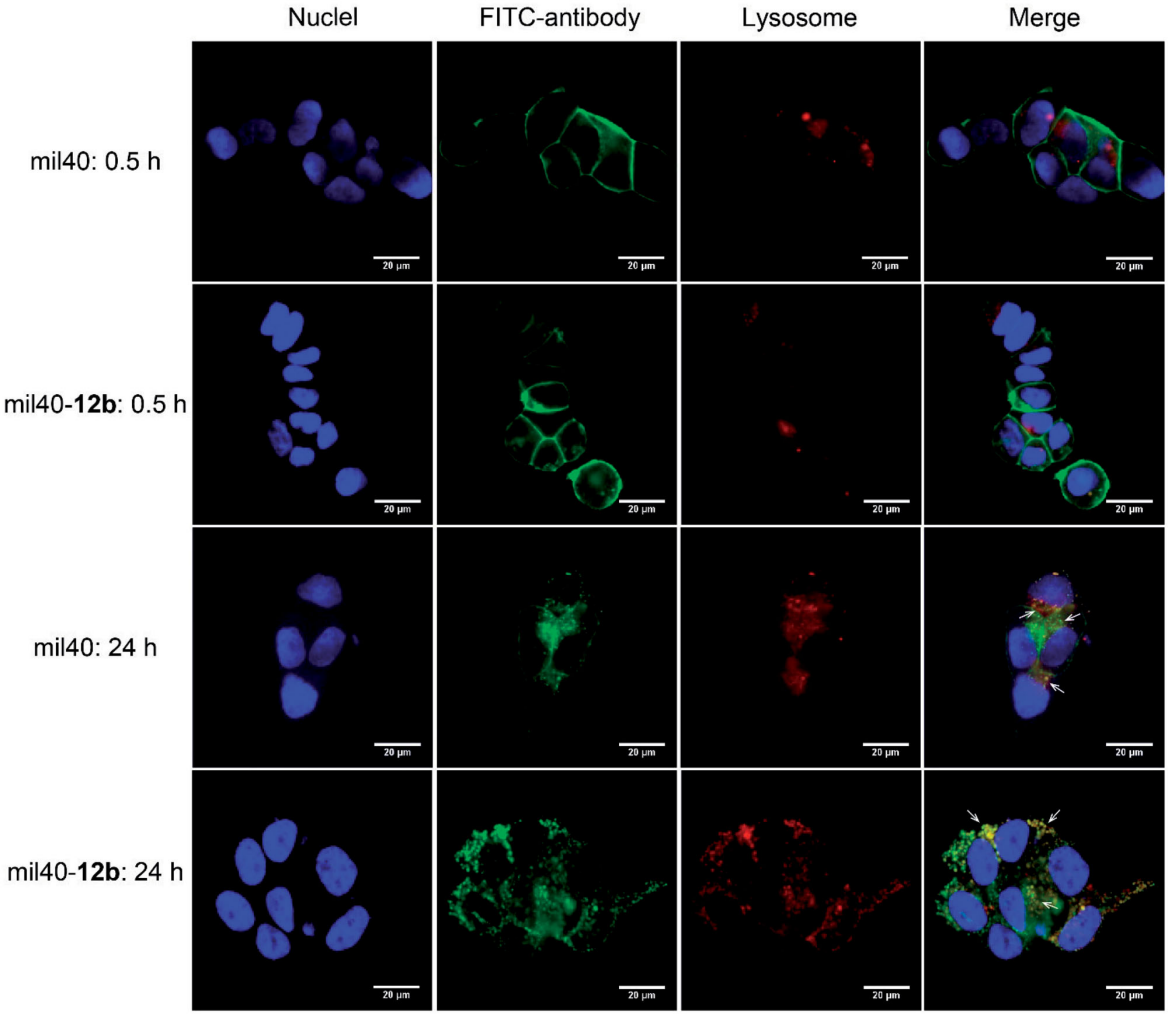
Cell binding and endocytosis of mil40-**12b** in the HER2^+^ BT-474 cells. Cells were incubated at 4 °C with FITC-labeled mil40 and mil40-**12b** for 0.5 h or incubated at 37 °C for 24 h. Each experimental group contains two replicates. Scale bar = 20 μm.

In view of poor HIC drug distribution of mil40-**12c**, mil40-**12b** was selected to further elucidate the intracellular trafficking process in the HER2^+^ breast tumor cell line BT-474. As shown in Figure 5, both the ADC mil40-**12b** and the naked antibody mil40 were bound to the peripheral membrane of BT-474 cells (green) at 4 °C after 30 min but were not localized inside the BT-474 cells or colocalized with lysosomal markers (red). Meanwhile, after 24 h of incubation at 37 °C, the green signals from both mil40 and mil40-**12b** appeared in the cells, and through intracellular signal colocalization, the lysosome sections were found to be yellow (Pearson’s correlation = 0.794; Mander’s overlap = 0.951). This test shows that maleamic methyl ester-based ADCs can be internalized into HER2^+^ tumor cells and transported into lysosomes for degradation.

### 3.5. Cell cycle arrest analysis

**Figure 6.**
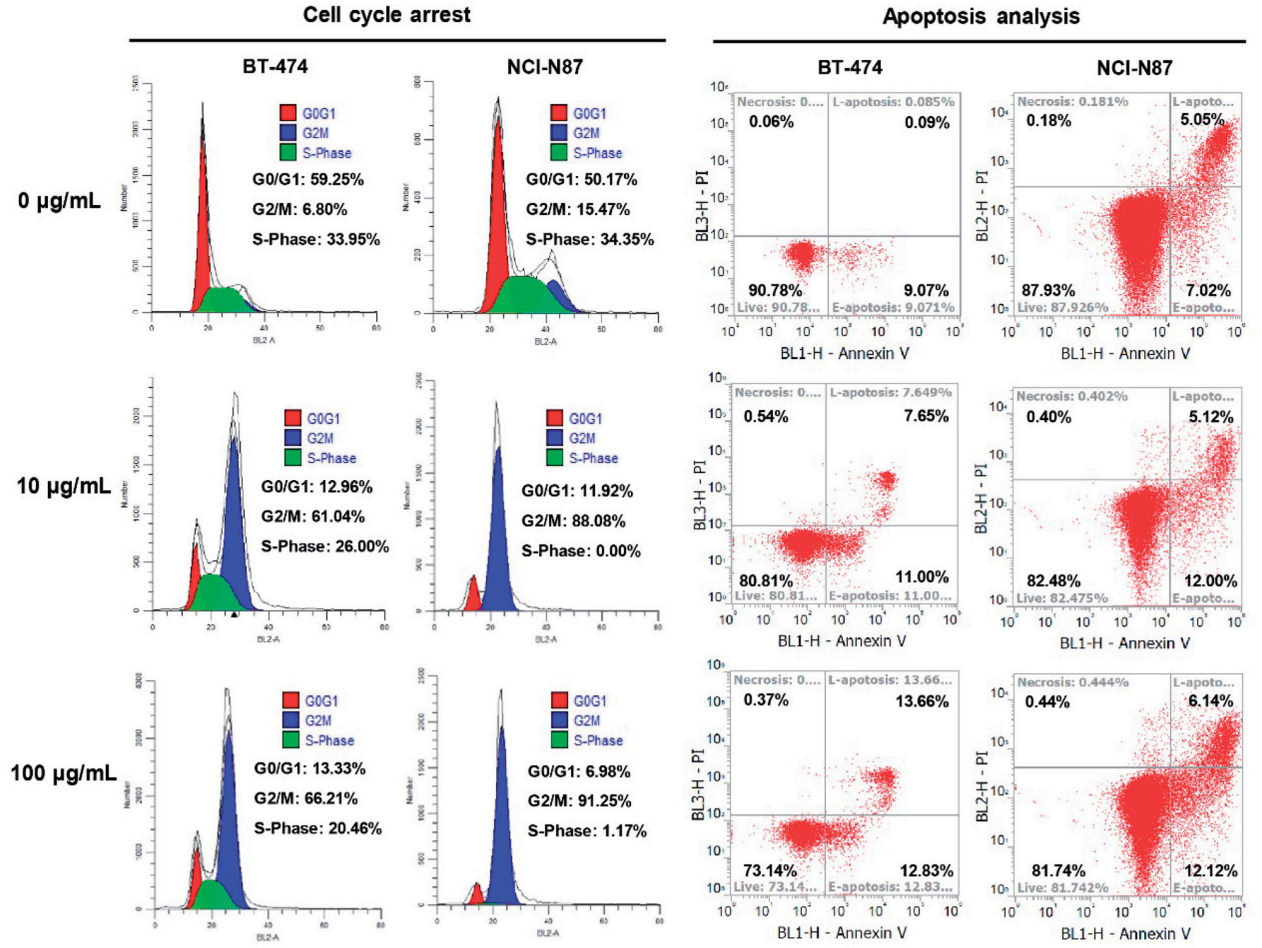
Flow cytometry analysis of the ADC inhibitory effects on cell proliferation. Cell cycle arrest and apoptosis analysis were executed in BT-474 and NCI-N87 cell lines, and the cells were incubated with different concentrations of mil40-**12b** for 24 h. Each experimental group contains two replicates.

We further performed flow cytometry analysis on the optimized mil40-**12b** to preliminarily verify its mechanism of action. As shown in Figure 6, after incubation with mil40-**12b** for 24 h, both BT-474 and NCI-N87 cells showed significant accumulation in the G_2_/M phases of the cell cycle and the magnitude of the increase was concentration-dependent. This conclusion is the same as that of the reported mechanism of MMAE loaded by the antibody (Francisco et al., 2003). In addition, after treatment with mil40-**12b**, BT-474 and NCI-N87 tumor cells showed dose-dependent apoptosis, which may be caused by mil40-**12b** blocking the cell cycle in BT-474 and NCI-N87 cells. The study preliminarily showed that mil40-**12b** can inhibit the tumor cell cycle in the G_2_/M stage in a concentration-dependent manner to inhibit tumor proliferation.

### 3.6. *In vivo* potency and safety

**Figure 7.**
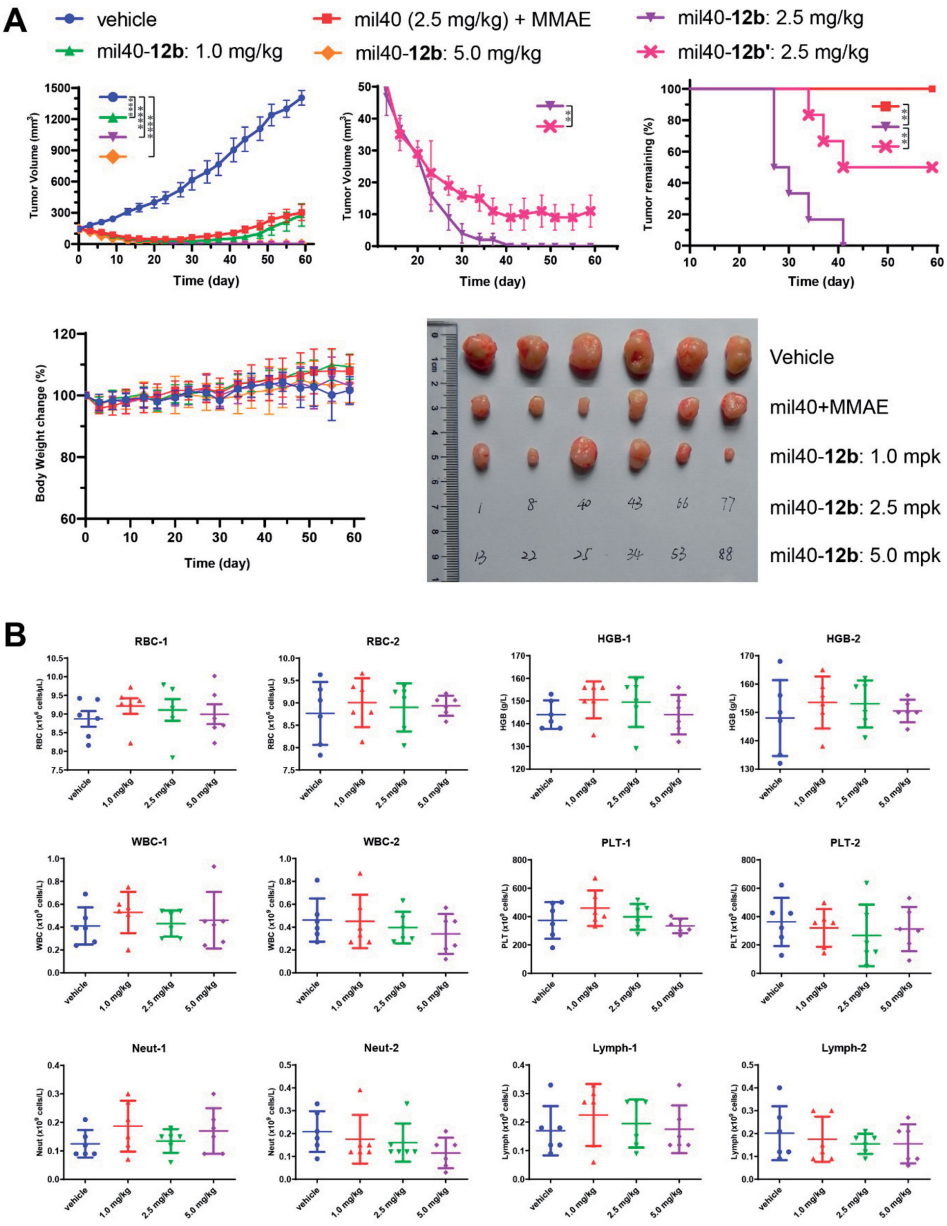
(A) Xenograft studies of mil40-**12b**: changes in tumor volume and body weights of the test mice. The results are shown as the means ± SD, *n* = 6/group. Tumor inhibition comparison for treatment groups performed by unpaired two-tailed *t*-test, significance exhibited as **p< .01, ****p< .0001. (B) Hematological analysis of mil40-**12b**, numbers ‘1’ and ‘2’ represent the first and second samplings on days 28 and 58, respectively. The results are shown as the means ± SD, *n* = 6/group. Statistical difference of *p* values between each group is shown in Supporting Information, Table S1. RBC: red blood cells; HGB: hemoglobin; WBC: white blood cells; PLT: platelets; Neut: neutrophils; Lymph: lymphocytes.

In an SCID mice xenograft model with BT-474 cells (Figure 7(A)), the *in vivo* efficacy of mil40-**12b** was not only significantly better than that of the combination of the same dose (2.5 mg/kg) of naked antibody and MMAE (*p*= .0031), but also significantly better than that of the corresponding maleimide-based ADC mil40-**12b′** (mil40-mc-VA-PABC-MMAE) (Wang et al., 2017). Notably, the tumors of all tested animals treated with mil40-**12b** completely disappeared at a dose of 2.5 mg/kg, and did not relapse within 1 month after stopping treatment. For comparison, the tumors in the tested mice in the mil40-**12b′** treatment group could not be completely resolved (*p*= .0061). Moreover, it can be seen from the tumor remaining curve that the tumors of the test mice in the mil40-**12b** group disappeared faster and more thoroughly than that of mil40-**12b′** group. In addition, the mil40-**12b** groups showed no significant weight loss in the mice during treatment, initially showing its good tolerance at therapeutic doses. This study shows that the maleamic methyl ester-based ADC demonstrated superior efficacy over the corresponding traditional maleimide-based ADC *in vivo*.

To verify the safety of mil40-**12b** at the therapeutic dose, the hematological parameters of the test animals were evaluated after treatment. At the end of the dosing phase in the BT-474 xenograft model (day 28) and at the end of the observation period (day 58), all tested animals were bled for hematological analysis. The results showed that mil40-**12b** displayed no significant hemotoxicity or off-target bone marrow toxicity at the therapeutic dose (Figure 7(B)). The three ADC treatment groups did not show statistically significant toxicity compared to the vehicle control group (Supporting Information, Table S1).

In addition, histopathological studies were also performed on the mil40-**12b** administered group. In the histopathological evaluation by H&E staining of the liver, heart, and lung, and bone marrow smear, there was no significant difference in the ADC group compared with the vehicle (Supporting Information, Figure S3), which further confirmed the safety of mil40-**12b** at the therapeutic dose.

### 3.7. Acute toxicity studies

Maleamic methyl ester-based mil40-**12b** has shown ideal safety characteristics at a therapeutic dose, which is similar to the results of our previously reported maleimide-based ADC mil40-**12b′** (Wang et al., 2017). We therefore conducted a toxicity test at a higher dose to compare the safety differences between the two ADCs. The data regarding the changes in the tested mice body weights over time preliminarily showed that after administration of a single dose of 20 mg/kg, mil40-**12b** and mil40-**12b′** had no significant changes compared with the vehicle over the same time period (Supporting Information, Figure S4).

In the above safety study with a dose of 20 mg/kg, we focused on the changes in Neut counts of the tested animals, which has been proven to be an important hematological toxicity index altered by ADCs loaded MMAE (Lyon et al., 2014). As shown in Supporting Information, Figure S5, on the 7th day after administration, the Neut count in the mil40-**12b** therapy group was slightly lower than vehicle (*p*= .2668); in contrast, the Neut count in the control group (mil40-**12b′**) was significantly lower than that of the vehicle group (*p*= .0306). In addition, the total white blood cell and lymphocyte counts of the test animals in the mil40-**12b′** group were visually reduced by 1/3. Notably, there were no significant differences in the main hematological parameters between the two groups on the 4th day and the 14th day. This test shows that mil40-**12b** has relatively lower hematological toxicity than mil40-**12b′**.

**Figure 8.**
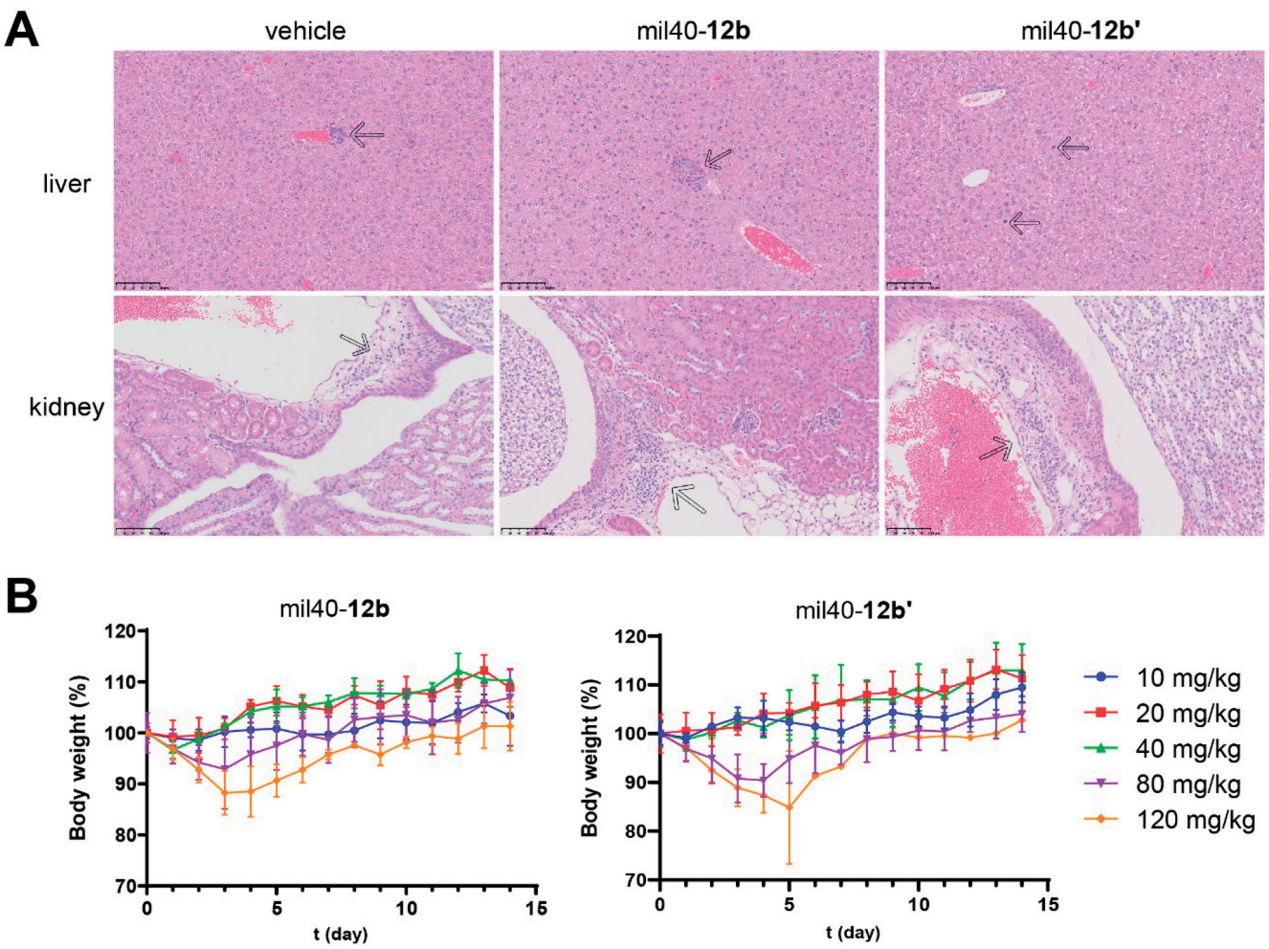
(A) Histopathological studies of the ADC mil40-**12b** at 20 mg/kg. Traditional maleimide linker-containing ADC mil40-**12b′** was used as a control; *n* = 3/group. Arrows indicate mild multifocal mononuclear cell infiltration, a common incidental or spontaneous lesion in experimental CD-1 mice, independent of the test substance. (B) Changes in the body weights of female CD-1 mice. The data points represent the mean body weight ± SD, *n*= 3/group.

Seven days after administration, mice in the mil40-**12b** and mil40-**12b′** groups (three mice/group) were also randomly selected for gross pathological observation. Compared with the saline vehicle group, all mice in the mil40-**12b** group had no significant changes related to the test ADC. In contrast, in the mil40-**12b′** group, bilateral uterine dilation and mild mixed inflammatory cell infiltration in the lamina propria was found in one animal (1/3) on the 7th day after administration (Supporting Information, Figure S6). In addition, histopathological studies showed that mice in both mil40-**12b** and mil40-**12b′** groups had no significant renal histotoxicity, while the mil40-**12b′** group showed a slight increase in hepatocyte mitosis compared with the mil40-**12b** group (Figure 8(A)). In other respects, all animals, including those in the vehicle group, showed mild multifocal mononuclear or mixed inflammatory cell infiltration in the liver and renal interstitium, which may be a common spontaneous or occasional disease in CD-1 mice. In summary, mil40-**12b** has a therapeutic safety advantage over mil40-**12b′** at high dose.

### 3.8. High-dose tolerance study

In order to evaluate the safety profile of mil40-**12b** at higher doses, we evaluated the MTDs of mil40-**12b** and mil40-**12b′** in female CD-1 mice. As shown in Figure 8(B), the test mice in the mil40-**12b** and mil40-**12b′** groups did not experience significant weight loss or adverse reactions at a doses of 40 mg/kg or lower. At a dose of 80 mg/kg, the body weights of the mice in the mil40-**12b** and mil40-**12b′** groups decreased by 7.13% and 9.59% from days 0 to 4, and the average body weight increased by 14.11% and 13.7% from days 5 to 15, respectively. At a dose of 120 mg/kg, the body weights of test mice in the mil40-**12b** and mil40-**12b′** groups decreased by 11.72% and 12.61% from days 0 to 4, while the average body weight increased by 13.06% and 9.79% from days 5 to 15.

In addition, at a dose of 80 mg/kg, the adverse reaction symptoms observed in the mil40-**12b** group of mice included minor alopecia, escharosis, and soft stools, while the additional side effect of small scattered granular pimples in the skin of the dorsal neck occurred in the mil40-**12b′** control group (3/3 occurred). At a dose of 120 mg/kg, the adverse reaction symptoms observed in mice in the mil40-**12b** group included alopecia, escharosis, and half-closed eyes. Notably, on the 5th day after dosing, mice in the mil40-**12b′** group were found dead (1/3 deaths) after a weight loss of 25.52%, and various adverse reactions, such as soft stools and perianal infection, were observed before death. In summary, the MTD of mil40-**12b** to healthy female CD-1 mice can reach 120 mg/kg, while mil40-**12b′** is tolerated at a dose of 80 mg/kg but not tolerated at a dose of 120 mg/kg. The results of this test comprehensively show that mil40-**12b** has a safety advantage over traditional mil40-**12b′**.

## 4. Discussion and conclusions

ADCs are designed to exclusively deliver cytotoxins to tumor cells while reducing systemic toxicity (Nadkarni et al., 2021). However, there are still many challenges in the clinical ADCs, among which toxic side effects are most formidable (Coats et al., 2019; Zhao et al., 2020). Generally, the toxicity of almost ADCs is mainly caused by their cytotoxins, as the toxicity of ADCs is mainly related to their carried payloads (Donaghy, 2016; Zhao et al., 2020).

The development of a new generation of linkers has always been the focus of the future development of ADCs to improve the therapeutic window (Srinivasarao et al., 2015; Beck et al., 2017; Bargh et al., 2019; Pryyma et al., 2020). Despite the well-known stability defects under physiological conditions, the thiosuccinimide produced by the Michael addition reaction between a thiol and maleimide is still widely used as a coupling joint in the structure of ADCs being developed on the market and clinically (Basle et al., 2010; Henkel et al., 2016; Nadkarni et al., 2021). At present, the design of novel generations of maleimide-type reagents has been reported; for example, *N*-aryl maleimides (Christie et al., 2015), β-amino maleimides (Lyon et al., 2014), acetal containing maleimides (Dovgan et al., 2016), and PEG-containing maleimides (Tumey et al., 2014). However, the druggability of ADCs based on these linkers still requires substantial clinical verification.

In this study, we first explored a variety of groups with potential Michael’s addition systems based on maleic acid amide, fumaramide methyl ester, and maleamic methyl ester. By coupling with the thiol of the antibodies, a novel linker based on maleamic methyl ester joint was screened out. This type of linker is easy to prepare and retains almost all of the advantages of the traditional maleimide system, and a stable ring-opening ADC can be obtained through one-step antibody coupling. In addition, the potential ester hydrolysate of this ring-opening product in plasma is exactly the same as that of the ideal ring-opening product of traditional ADCs based on maleimide joints. Further research found that this ring-opening product can undergo Michael’s addition reactions with many types of thiols, indicating its potential application value in the field of biomacromolecule coupling.

In summary, by screening a variety of maleimide derivatives, we used the maleamic methyl ester group as the thiol-reactive joint for the first time to prepare novel thiol-linked ADCs. This type of linker retains almost all of the advantages of the traditional maleimide system, and the prepared ADCs have better stability and *in vivo* efficacy than traditional ADCs based on maleimide joints. Further safety studies, such as hematology, histopathology, and MTD, have shown that the preferred ADC mil40-**12b** based on the maleamic methyl ester joint has safety advantages over the traditional ADC mil40-**12b′** based on the maleimide joint, and the MTD of the former increased significantly to 120 mg/kg. This strategy provides a universal alternative to traditional maleimide linkers without changing the stable medicinal linker structure of existing ADCs. It is therefore expected to further improve the drug therapeutic index, and play a role in promoting the rapid verification and industrialization of ADCs.

## Supplementary Material

Supplemental Material
