# Supplementary material for: Development of applicable thiol-linked antibody–drug conjugates with improved stability and therapeutic index
Source: Drug Deliv. 2022 Mar 4;29(1):754–66. doi: 10.1080/10717544.2022.2039807 (PMC8933021; doi:10.1080/10717544.2022.2039807)
Supplement: Supplemental Material [file IDRD_A_2039807_SM6349.docx]

**Supporting Information**

**Scheme S1.** Synthesis of the maleic acid amide-based linker-MMAE conjugates. Reagents and conditions: (**a**) **9a** / **9b**, DMF, room temperature (RT), overnight; (**b**) bis(4-nitrophenyl) carbonate, DIPEA, DMF, RT, 5 h; (**c**) MMAE, HOBt, DIPEA, DMF, RT, overnight.

**Scheme S2.** Synthesis of the fumaramide methyl ester-based linker-MMAE conjugates. Reagents and conditions: (**a**) **2b**, isobutyl chloroformate, *N*-methylmorpholine, DMF, -20 °C ~ RT, 3 h; (**b**) EDCI, HOBt, DIPEA, DMF, RT, 10 h; (**c**) bis(4-nitrophenyl) carbonate, DIPEA, DMF, RT, 5 h; (**d**) MMAE, HOBt, DIPEA, DMF, RT, overnight.

**Scheme S3.** Reagents and conditions: (**a**) AcOH, RT, 10 ~ 14 h; (**b**) trifluoroacetic anhydride, NHS, 2,4,6-trimethylpyridine, DMF, 0 °C, 1 h; (**c**) MeI, K_2_CO_3_, DMF, RT, 2 h; (**d**) NHS, DCC, *L*-Ala/*L*-Cit, THF, DME, RT, 16 h; (**e**) *p*-aminobenzyl alcohol, EEDQ, MeOH/DCM, RT, 32 h; (**f**) piperidine, DMF, RT, 2 h; (**g**) **5a** ~ **5b**, DMF, RT, overnight; (**h**) bis(4-nitrophenyl) carbonate, DIPEA, DMF, RT, 7 h; (**i**) HOBt, MMAE, DIPEA, DMF, RT, overnight.

**Scheme S4.** Synthesis of the novel maleamic methyl ester-based model small molecule conjugates. Reagents and conditions: (**a**) aniline, isobutyl chloroformate, *N*-methylmorpholine, THF, -20 °C ~ RT, 2 h; (**b**) MeI, K_2_CO_3_, DMF/DCM, RT, 2 h; (**c**) R-SH (2-hydroxy-1-ethanethiol, benzyl mercaptan, (*R*)-2-acetamido-*N*-benzyl-3-mercaptopropanamide), MeOH, RT, 0.5 ~ 2 h.

**Scheme S5.** Synthesis of the maleamic methyl ester-based and maleimide-based model small molecules. Reagents and conditions: (**a**) phenylamine, DCC, THF, RT, overnight; (**b**) TFA, DCM, RT, overnight; (**c**) *N*-methyl-*N*-Boc-valine, EDCI, HOBt, DIPEA, DCM, RT, overnight; (**d**) TFA, DCM, RT, 3 h; EA, H_2_O, NaHCO_3_; (**e**) **11c**, HOBt, DIPEA, DMF, RT, 18 h; (**f**) (*R*)-2-acetamido-*N*-benzyl-3-mercaptopropanamide, DIPEA, MeOH, RT, 30 min; (**g**) **S23**, HOBt, DIPEA, DMF, RT, overnight; (**h**) (*R*)-2-acetamido-*N*-benzyl-3-mercaptopropanamide, TEA, MeOH, RT, 30 min.

**Table S1.** The *p* value between vehicle and ADC treatment groups.

|  | vehicle vs 1.0 mg/kg | vehicle vs 2.5 mg/kg | vehicle vs 5.0 mg/kg |
| --- | --- | --- | --- |
| RBC-1 | 0.27 | 0.52 | 0.72 |
| RBC-2 | 0.53 | 0.72 | 0.59 |
| HGB-1 | 0.15 | 0.31 | 0.98 |
| HGB-2 | 0.43 | 0.45 | 0.67 |
| WBC-1 | 0.26 | 0.81 | 0.41 |
| WBC-2 | 0.93 | 0.50 | 0.27 |
| PLT-1 | 0.27 | 0.71 | 0.51 |
| PLT-2 | 0.64 | 0.42 | 0.61 |
| Neut-1 | 0.17 | 0.71 | 0.26 |
| Neut-2 | 0.57 | 0.36 | 0.10 |
| Lymph-1 | 0.35 | 0.62 | 0.92 |
| Lymph-2 | 0.51 | 0.34 | 0.37 |

Note: numbers “1” and “2” represent the first and second sampling on days 28 and days 58, respectively.


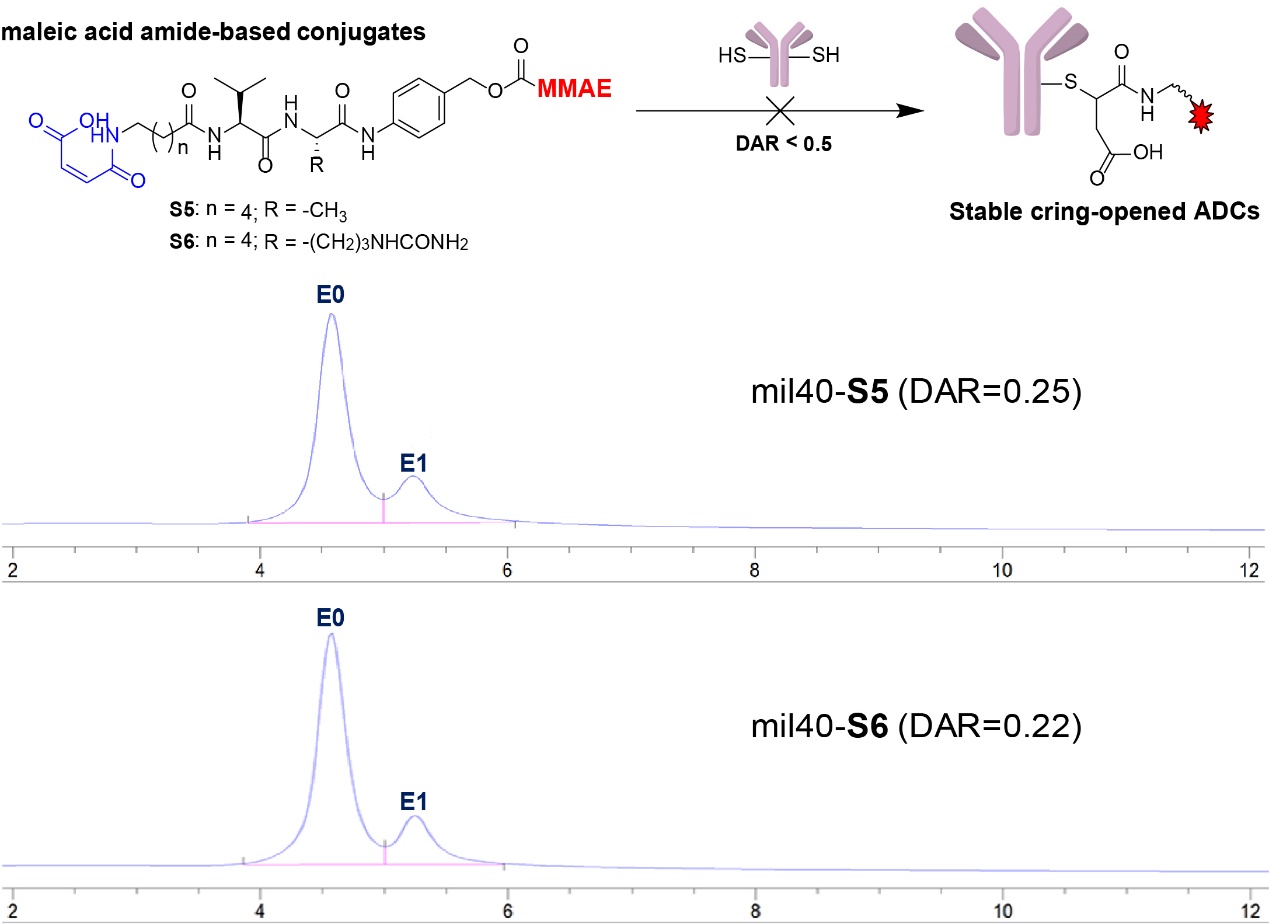


**Figure S1.** HIC analysis of the ADCs that with the maleic acid amide-based linkers. The DAR values of the two ADCs (mil40-**S5** and mil40-**S6**) are 0.25 and 0.22, respectively. E0 and E1 represent the DAR value of 0 and 1.


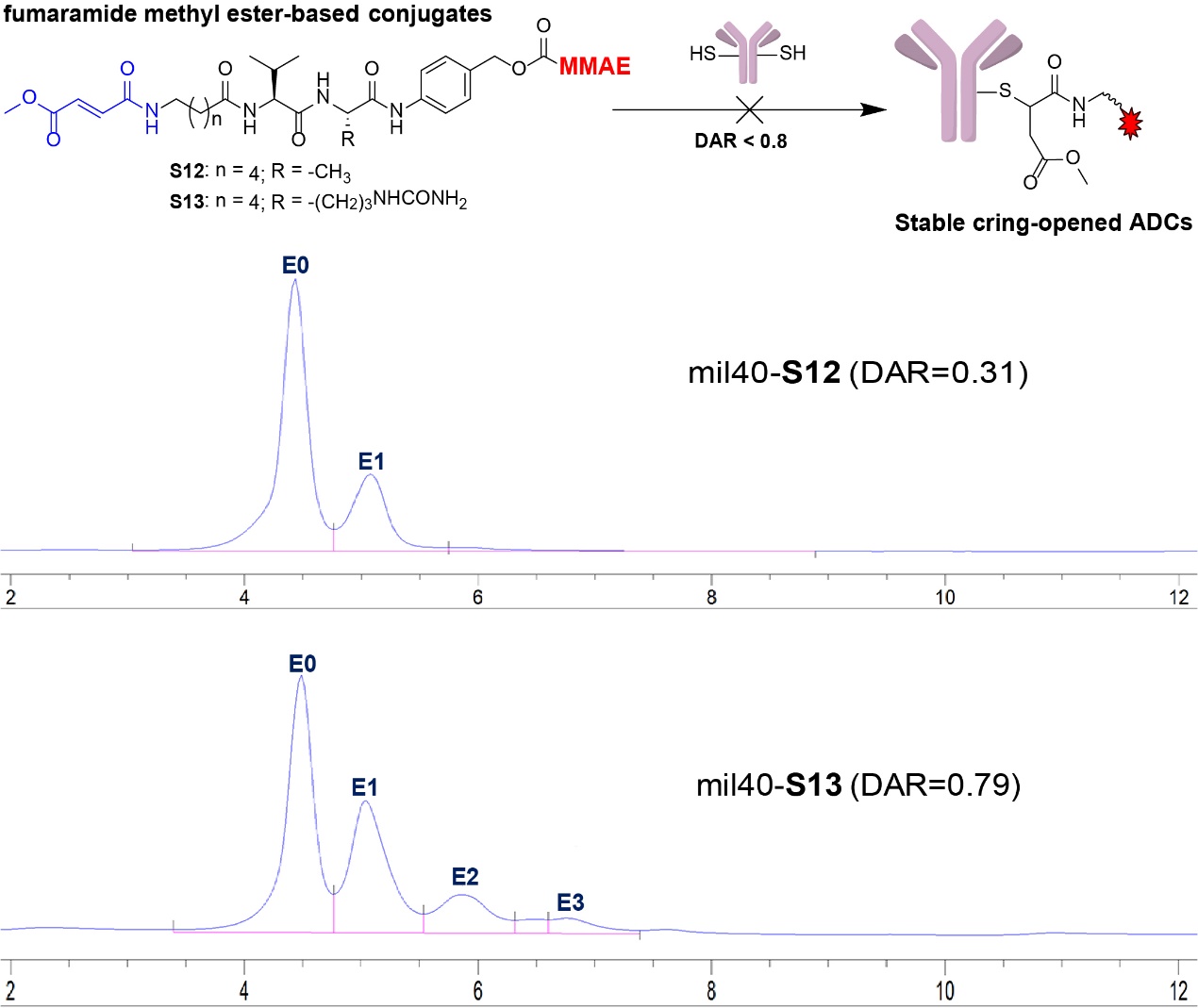


**Figure S2.** HIC analysis of the ADCs that with the fumaramide methyl ester-based linkers. The DAR values of the two ADCs (mil40-**S12** and mil40-**S13**) are 0.31 and 0.79, respectively. E0, E1, E2, and E3 represent the DAR value of 0, 1, 2, and 3.


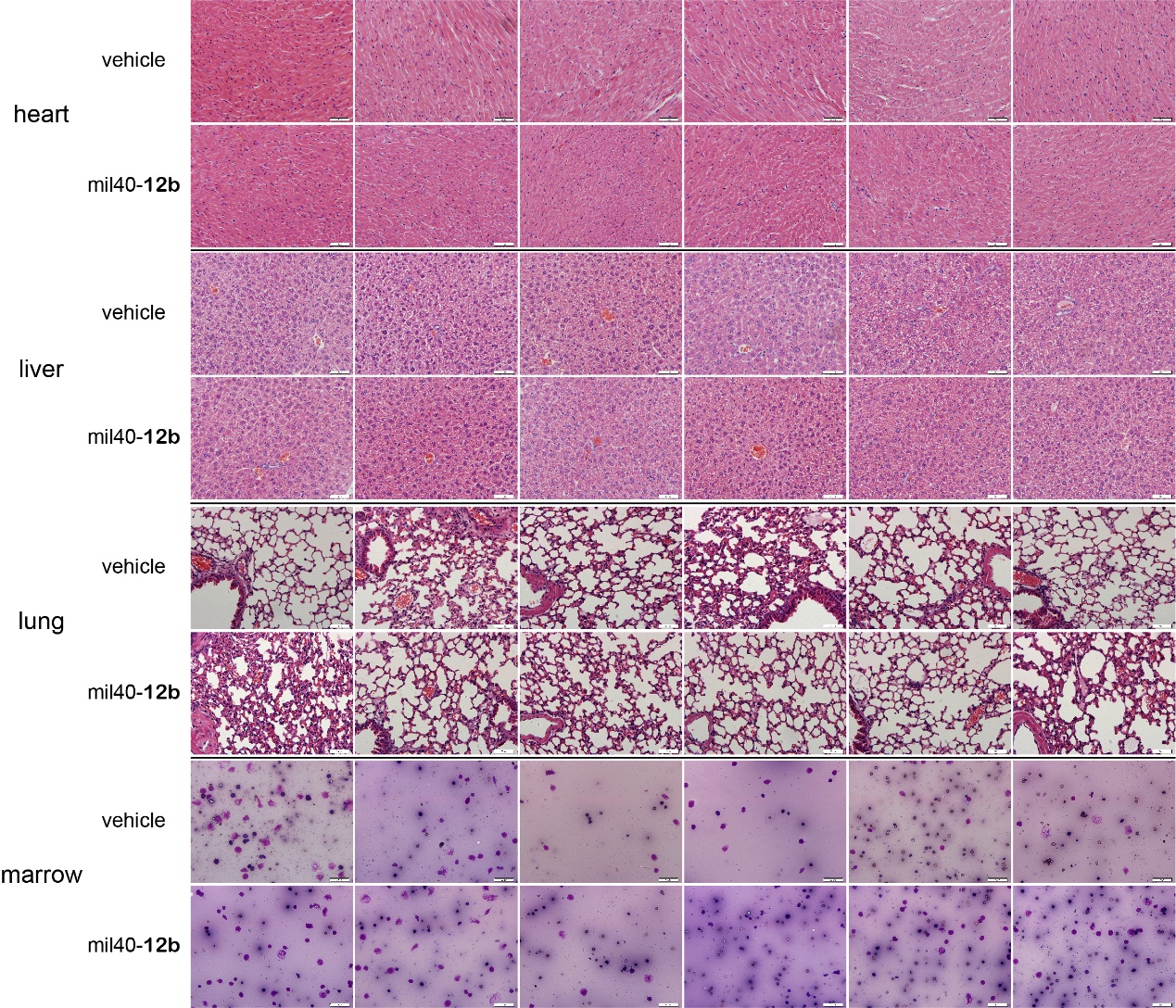


**Figure S3.** Histopathological studies of the maleamic methyl ester-based ADC mil40-**12b** at 2.5 mg/kg. The actual size of each picture is 425 × 320 μm; scale bar: 46:1; n = 6/group.

**Figure S4.** Changes in body weights of the test mice after administration of a single dose of 20 mg/kg. The results are shown as the means ± SD, n = 9/group.

**Figure S5.** Hematological analysis of the maleamic methyl ester-based ADC mil40-**12b** at the dose of 20 mg/kg. The results are shown as the means ± SD, n = 3/group. The comparison for treatment groups performed by unpaired two-tailed *t* test, significance exhibited as * (*P* < 0.05).


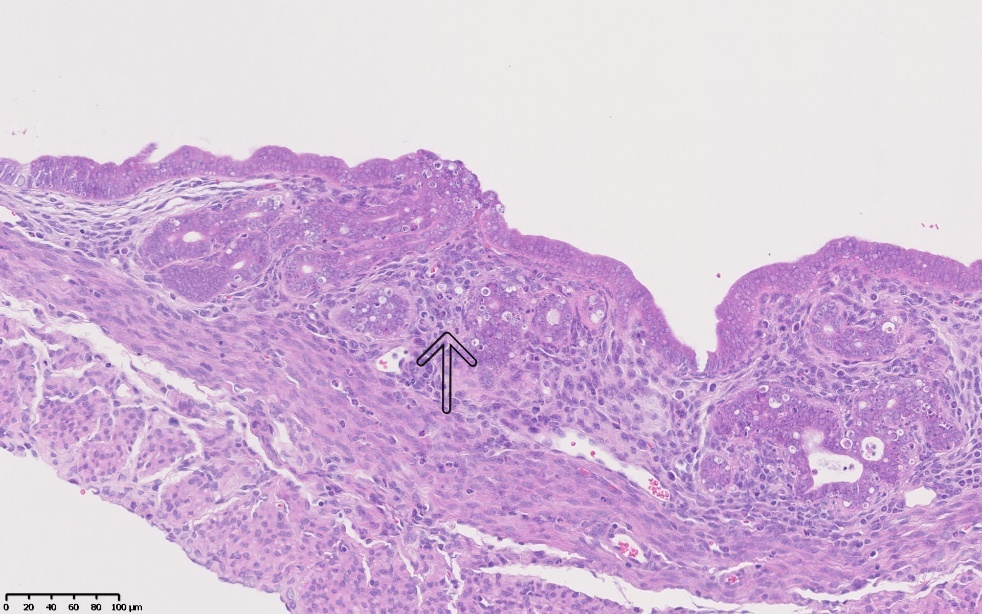


**Figure S6.** The uterine histopathology study (H&E dyeing) of the traditional maleimide-based ADC mil40-**12b’**. Female CD-1 mice were injected with mil40-**12b’** at a dose of 20 mg/kg via tail vein. Arrows indicate mild mixed inflammatory cell infiltration in the lamina propria.

**Synthesis of the** **compounds**

*(Z)-4-((2-carboxyethyl)amino)-4-oxobut-2-enoic acid (compound* ***3a****)*

β-aminopropionic acid (4.13 g, 46.35 mmol) was dissolved in acetic acid (200 mL), and then maleic anhydride (5.0 g, 51 mmol) was slowly added to the mixture. The reaction continued at room temperature (RT) for 10 h. After completion of the reaction, the mixture was filtered, and the filter cake was washed with acetonitrile to obtain compound **3a** as a white solid (7.72 g, 89% yield). ^1^H-NMR (400 MHz, DMSO-*d6*): δ 14.28 (br, 1H), 12.72 (br, 1H), 9.09 (s, 1H), 6.40 (d, *J* = 12.6 Hz, 1H), 6.24 (d, *J* = 12.6 Hz, 1H), 3.37 (t, *J* = 6.7 Hz, 2H), 2.47 (t, *J* = 6.7 Hz, 2H). MS (ESI) m/z: 186.04 [M-H]^-^.

*(Z)-6-(3-carboxyacrylamido)hexanoic acid (compound* ***3b****)*

6-aminocaproic acid (3.94 g, 30.0 mmol) was dissolved in acetic acid (150 mL), and then maleic anhydride (2.94 g, 30.0 mmol) was slowly added to the mixture. The reaction continued at RT for 14 h. After completion of the reaction, the mixture was filtered, and the filter cake was washed with acetonitrile to obtain compound **3b** as a white solid (5.60 g, 81% yield). ^1^H-NMR (400 MHz, DMSO-*d6*): δ 15.08 (br, 1H), 11.92 (br, 1H), 9.12 (t, *J* = 4.2 Hz, 1H), 6.40 (d, *J* = 12.6 Hz, 1H), 6.24 (d, *J* = 12.6 Hz, 1H), 3.16 (q, *J* = 6.5 Hz, 2H), 2.20 (t, *J* = 7.3 Hz, 2H), 1.54-1.43 (m, 4H), 1.32-1.25 (m, 2H). MS (ESI) m/z: 228.09 [M-H]^−^.

*(Z)-4-((3-((2,5-dioxopyrrolidin-1-yl)oxy)-3-oxopropyl)amino)-4-oxobut-2-enoic acid (compound* ***4a****)*

Compound **3a** (2.0 g, 10.68 mmol) was dissolved in anhydrous *N*,*N*-dimethylformamide (DMF), and the mixture was cooled to -5 °C. After stirring for 10 min, *N*-hydroxysuccinimide (NHS) (2.46 g, 21.4 mmol), 2,4,6-trimethylpyridine (4.2 mL, 32.0 mmol) and trifluoroacetic anhydride (4.52 mL, 32.0 mmol) were added slowly in a dropwise fashion; then, the reaction continued at 0 °C for another 1 h. After completion of the reaction, the solution was poured into a dilute hydrochloric acid solution (100 mL) and extracted with an appropriate amount of chloroform (80 mL × 4). The organic phases were combined, washed with brine for three times, dried over sodium sulfate and concentrated *in vacuo* to obtain compound **4a** as a white solid (2.50 g, 82% yield). ^1^H-NMR (400 MHz, CDCl_3_): δ 8.22 (br, 1H), 6.39 (d, *J* = 12.6 Hz, 1H), 6.34 (d, *J* = 12.6 Hz, 1H), 3.77 (q, *J* = 6.1 Hz, 2H), 2.96 (t, *J* = 6.4 Hz, 2H), 2.89 (s, 4H). MS (ESI) m/z: 285.2 [M+H]^+^; 307.2 [M+Na]^+^; 283.2 [M-H]^−^.

*(Z)-4-((6-((2,5-dioxopyrrolidin-1-yl)oxy)-6-oxohexyl)amino)-4-oxobut-2-enoic acid (compound* ***4b****)*

Compound **3b** (4.0 g, 17.45 mmol) and NHS (8.03 g, 69.80 mmol) were dissolved in anhydrous DMF (70 mL), and the mixture was cooled to -5 °C. After stirring for 15 min, 2,4,6-trimethylpyridine (9.31 mL, 69.80 mmol) and trifluoroacetic anhydride (9.8 mL) were added slowly in a dropwise fashion; then, the reaction continued for another 1 h at 0 °C. After completion of the reaction, the solution was poured into dilute hydrochloric acid solution (200 mL) and extracted with appropriate amount of chloroform (100 mL × 4). The organic phases were combined, washed with brine three times, dried over sodium sulfate and concentrated *in vacuo* to obtain compound **4b** as a white solid (5.74 g, 90% yield). ^1^H-NMR (400 MHz, DMSO-*d6*): δ 15.13 (br, 1H), 9.11 (t, *J* = 5.0 Hz, 1H), 6.41 (d, *J* = 12.6 Hz, 1H), 6.23 (d, *J* = 12.6 Hz, 1H), 3.17 (q, *J* = 6.5 Hz, 2H), 2.81 (s, 1H), 2.67 (t, *J* = 7.3 Hz, 2H), 1.67-1.60 (m, 2H), 1.54-1.46 (m, 2H), 1.41-1.34 (m, 2H). MS (ESI) m/z: 327.5 [M+H]^+^; 325.4 [M-H]^−^.

*Methyl (Z)-4-((3-((2,5-dioxopyrrolidin-1-yl)oxy)-3-oxopropyl)amino)-4-oxobut-2-enoate (compound* ***5a****)*

Compound **4a** (2.40 g, 9.27 mmol) was dissolved in anhydrous DMF (30 mL), and then anhydrous K_2_CO_3_ (2.56 g, 18.53 mmol) was added. Methyl iodide (2.63 g, 18.53 mmol) was added dropwise with stirring at RT, and the reaction continued for 2 h after completion of the addition. The insoluble salt was removed by filtration, and the solvent was concentrated under reduced pressure. The residue was dissolved in ethyl acetate (EA), filtered, and washed three times with brine. After drying over MgSO_4_, the organic phase was concentrated under reduced pressure to give crude compound **5a** as a white solid (0.65 g, 24% yield). As the resulting product was found to be unstable during the purification process, compound **5a** was directly subjected to the next reaction without further purification or structural confirmation.

*2,5-dioxopyrrolidin-1-yl (Z)-6-(4-methoxy-4-oxobut-2-enamido)hexanoate (compound* ***5b****)*

Compound **4b** (1.50 g, 4.60 mmol) was dissolved in anhydrous DMF (25 mL), and then anhydrous K_2_CO_3_ (1.27 g, 9.2 mmol) was added. Methyl iodide (1.30 g, 9.20 mmol) was added dropwise with stirring at RT, and the reaction continued for 2 h after completion of the addition. The insoluble salt was removed by filtration, and the solvent was concentrated under reduced pressure. The residue was dissolved in EA, filtered, and washed three times with brine. After drying over MgSO_4_, the organic phase was concentrated under reduced pressure to give the crude product. Further purification was performed by silica column chromatography (PE/EA = 1:1) to give compound **5b** as a white solid (1.10 g, 66% yield). ^1^H-NMR (400 MHz, CDCl_3_): δ 8.21 (br, 1H), 6.35 (d, *J* = 12.6 Hz, 1H), 6.13 (d, *J* = 12.6 Hz, 1H), 3.80 (s, 3H), 3.35 (q, *J* = 6.3 Hz, 2H), 2.84 (s, 4H), 2.63 (t, *J* = 7.3 Hz, 2H), 1.80 (m, 4H), 1.63 (m, 2H), 1.49 (m, 2H). MS (ESI) m/z: 341.2 [M+H]^+^; 363.2 [M+Na]^+^.

*(((9H-fluoren-9-yl)methoxy)carbonyl)-L-valyl-L-alanine (compound* ***7a****)*

Compound **6** (25.0 g, 74.0 mmol), NHS (8.52 g, 74.0 mmol) and DCC (15.27 g, 74.0 mmol) in tetrahydrofuran (THF) (250 mL) were stirred at RT for 16 h. The reaction mixture was cooled to 0 °C for 2 h and then filtered to remove the insoluble dicyclohexylurea. The filter cake was washed with THF, and then the filtrate was evaporated under reduced pressure to give the crude product, which was used in the next step without further purification. The crude product was redissolved in dimethoxyethane (DME) (200 mL) and THF (100 mL), and then an aqueous sodium hydrogencarbonate solution of *L*-alanine (6.95 g, 78 mmol) was added. The reaction mixture was stirred at RT for 16 h, poured into aqueous citric acid solution (400 mL, 15%), and filtered after the filter cake was dried under reduced pressure to give the crude product. The crude product was suspended in ether, and purification was performed by ultrasound and filtration 3 times to give compound **7a** as a white solid (16.20 g, 53% yield). ^1^H-NMR (400 MHz, DMSO-*d6*): δ 12.48 (s, 1H), 8.25 (d, *J* = 6.9 Hz, 1H), 7.89 (d, *J* = 7.5 Hz, 2H), 7.74 (t, *J* = 6.6 Hz, 2H), 7.43 (m, 3H), 7.33 (m, 2H), 4.22 (m, 4H), 3.89 (t, *J* = 7.2 Hz, 1H), 1.96 (m, 1H), 1.27 (d, *J* = 7.3 Hz, 3H), 0.88 (dd, *J* = 6.8 Hz, 6H). MS (ESI) m/z: 411.3 [M+H]^+^; 433.4 [M+Na]^+^.

*(S)-2-((S)-2-((((9H-fluoren-9-yl)methoxy)carbonyl)amino)-3-methylbutanamido)-5-(carboxyamino)pentanoic acid (compound* ***7b****)*

Compound **7b** was synthesized according to the same experimental protocol described for **7a**. The yield of the white solid was 70%. ^1^H-NMR (400 MHz, DMSO-*d6*): δ 12.61 (s, 1H), 8.21 (d, *J* = 7.2 Hz, 1H), 7.89 (d, *J* = 7.6 Hz, 2H), 7.76 (t, *J* = 7.2 Hz, 2H), 7.43-7.33 (m, 5H), 5.98 (t, *J* = 4.8 Hz, 1H), 5.43 (s, 2H), 4.25 (m, 3H), 4.16 (q, 1H), 3.94 (t, *J* = 8.0 Hz, 1H), 2.96 (m, 2H), 1.98 (m, 1H), 1.72 (m, 1H), 1.58 (m, 1H), 1.42 (m, 2H), 0.89 (dd, *J* = 6.8 Hz, 6H). MS (ESI) m/z: 497.6 [M+H]^+^; 519.6 [M+Na]^+^.

*(9H-fluoren-9-yl)methyl ((S)-1-(((S)-1-((4-(hydroxymethyl)phenyl)amino)-1-oxopropan-2-yl)amino)-3-methyl-1-oxobutan-2-yl)carbamate (compound* ***8a****)*

To a solution of compound **7a** (5.50 g, 13.4 mmol) in DCM (150 mL) and methanol (75 mL), *p*-aminobenzyl alcohol (3.30 g, 26.8 mmol) and EEDQ (6.63 g, 26.8 mmol) were added. The mixture was stirred at RT for 32 h and then evaporated under reduced pressure to give the crude product. The crude product was suspended in ether, and purification was performed by ultrasound and filtration 3 times to give compound **8a** as a white solid (5.34 g, 77% yield). ^1^H-NMR (400 MHz, DMSO-*d6*): δ 9.96 (s, 1H), 8.22 (d, *J* = 7.0 Hz, 1H), 7.89 (d, *J* = 7.5 Hz, 2H), 7.75 (t, *J* = 7.1 Hz, 2H), 7.54 (m, *J* = 8.4 Hz, 4H), 7.47-7.40 (m, 3H), 7.34 (t, *J* = 7.4 Hz, 2H), 7.24 (t, *J* = 8.5 Hz, 2H), 5.13 (t, *J* = 5.7 Hz, 1H), 4.42 (m, 3H), 4.30-4.22 (m, 3H), 3.91 (t, *J* = 7.2 Hz, 1H), 2.00 (m, 1H), 1.31 (d, *J* = 7.1 Hz, 3H), 0.90 (d, *J* = 6.8 Hz, 3H), 0.86 (d, *J* = 6.8 Hz, 3H). MS (ESI) m/z: 516.4 [M+H]^+^; 538.3 [M+Na]^+^.

*(9H-fluoren-9-yl)methyl ((S)-1-(((S)-1-((4-(hydroxymethyl)phenyl)amino)-1-oxo-5-ureidopentan-2-yl)amino)-3-methyl-1-oxobutan-2-yl)carbamate (compound* ***8b****)*

Compound **8b** was synthesized according to the same experimental protocol described for compound **8a**. The yield of the light yellow solid was 92%. ^1^H-NMR (400 MHz, DMSO-*d6*): δ 9.99 (s, 1H), 8.12 (d, *J* = 7.2 Hz, 1H), 7.89 (d, *J* = 7.6 Hz, 2H), 7.74 (t, *J* = 7.2 Hz, 2H), 7.54 (d, *J* = 8.4 Hz, 2H), 7.46-7.32 (m, 5H), 7.23 (d, *J* = 8.4 Hz, 2H), 5.98 (t, *J* = 5.6 Hz, 1H), 5.42 (s, 2H), 5.12 (s, 1H), 4.41 (m, 3H), 4.30 (q, *J* = 7.6 Hz, 1H), 4.23 (q, 2H), 3.92 (q, 1H), 3.00 (m, 2H), 1.98 (m, 1H), 1.69 (m, 1H), 1.57 (m, 1H), 1.41 (m, 2H), 0.86 (dd, *J* = 6.8 Hz, 6H). MS (ESI) m/z: 624.5 [M+Na]^+^.

*(S)-2-amino-N-((S)-1-((4-(hydroxymethyl)phenyl)amino)-1-oxopropan-2-yl)-3-methylbutanamide (compound* ***9a****)*

Piperidine (2 mL) was added to a solution of compound **8a** (4.0 g, 7.76 mmol) in DMF (40 mL). The mixture was stirred at RT for 2 h and then evaporated under reduced pressure to give the crude product. Purification was performed by silica column chromatography (DCM/MeOH = 5:1) to give compound **9a** as a white solid (2.05 g, 90% yield). ^1^H-NMR (400 MHz, DMSO-*d6*): δ 10.00 (s, 1H), 8.18 (s, 1H), 7.53 (d, *J* = 7.6 Hz, 2H), 7.24 (d, *J* = 7.6 Hz, 2H), 5.13 (s, 1H), 4.48 (s, 1H), 4.43 (s, 1H), 3.00 (d, *J* = 5.1 Hz, 1H), 2.73 (s, 1H), 1.92 (m, 1H), 1.29 (d, *J* = 6.9 Hz, 3H), 0.78 (dd, *J* = 6.8 Hz, 6H). MS (ESI) m/z: 294.2 [M+H]^+^; 316.2 [M+Na]^+^.

*(S)-2-((S)-2-amino-3-methylbutanamido)-N-(4-(hydroxymethyl)phenyl)-5-ureidopentanamide (compound* ***9b****)*

Compound **9b** was synthesized according to the same experimental protocol described for compound **9a**. The yield of the light yellow solid was 84%. ^1^H-NMR (400 MHz, DMSO-*d6*): δ 10.07 (s, 1H), 8.17 (br, 1H), 7.54 (d, *J* = 8.4 Hz, 2H), 7.23 (d, *J* = 8.4 Hz, 2H), 6.01 (br, 1H), 5.44 (s, 2H), 5.13 (br, 1H), 4.47 (s, 1H), 4.43 (s, 2H), 3.05 (d, *J* = 6.5 Hz, 1H), 2.95 (m, 2H), 1.94 (m, 1H), 1.64 (m, 2H), 1.38 (m, 2H), 0.86 (dd, *J_1_* = 15.7 Hz, *J_2_* = 6.8 Hz, 6H). MS (ESI) m/z: 380.3 [M+H]^+^; 402.3 [M+Na]^+^.

*Methyl (Z)-4-((3-(((S)-1-(((S)-1-((4-(hydroxymethyl)phenyl)amino)-1-oxopropan-2-yl)amino)-3-methyl-1-oxobutan-2-yl)amino)-3-oxopropyl)amino)-4-oxobut-2-enoate (compound* ***10a****)*

Compound **5a** (0.60 g, 2.0 mmol) and compound **9a** (0.59 g, 2.0 mmol) were dissolved in anhydrous DMF (20 mL), and the mixture stirred at RT overnight. After completion of the reaction, the mixture was evaporated under reduced pressure to give the crude product. Purification was performed by silica column chromatography (DCM/MeOH = 10:1) to give compound **10a** as a white solid (0.59 g, 62% yield). ^1^H-NMR (400 MHz, DMSO-*d6*): δ 9.85 (s, 1H), 8.24 (t, *J* = 5.7 Hz, 1H), 8.19 (d, *J* = 7.3 Hz, 2H), 7.96 (d, *J* = 8.4 Hz, 2H), 7.54 (d, *J* = 8.4 Hz, 2H), 7.23 (d, *J* = 8.4 Hz, 2H), 6.27 (d, *J* = 12.6 Hz, 1H), 6.23 (d, *J* = 12.6 Hz, 1H), 5.11 (t, *J* = 5.7 Hz, 1H), 4.42 (d, *J* = 5.6 Hz, 2H), 4.39 (t, *J* = 7.0 Hz, 1H), 4.19 (t, *J* = 7.6 Hz, 1H), 3.64 (s, 3H), 3.27 (q, *J* = 5.9 Hz, 2H), 2.38 (t, *J* = 7.0 Hz, 2H), 1.97 (m, *J* = 6.8 Hz, 1H), 1.30 (d, *J* = 7.0 Hz, 3H), 0.85 (dd, *J_1_* = 15.7 Hz, *J_2_* = 6.8 Hz, 6H). MS (ESI) m/z: 477.24 [M+H]^+^; 499.22 [M+Na]^+^; 975.45 [2M+Na]^+^.

*Methyl (Z)-4-((6-(((S)-1-(((S)-1-((4-(hydroxymethyl)phenyl)amino)-1-oxopropan-2-yl)amino)-3-methyl-1-oxobutan-2-yl)amino)-6-oxohexyl)amino)-4-oxobut-2-enoate (compound* ***10b****)*

Compound **5b** (0.50 g, 1.70 mmol) and compound **9a** (0.58 g, 1.70 mmol) were dissolved in anhydrous DMF (15 mL), and the mixture stirred at RT overnight. After completion of the reaction, the mixture was evaporated under reduced pressure to give the crude product. The obtained crude product was further dispersed and stirred in diethyl ether, and the filter cake obtained after filtration was dried to give compound **10b** as a white powdery solid (0.73 g, 83% yield). ^1^H-NMR (400 MHz, DMSO-*d6*): δ 9.84 (s, 1H), 8.15 (m, 2H), 7.81 (d, *J* = 8.7 Hz, 1H), 7.53 (d, *J* = 8.4 Hz, 2H), 7.23 (d, *J* = 8.4 Hz, 2H), 6.27 (d, *J* = 12.6 Hz, 1H), 6.22 (d, *J* = 12.6 Hz, 1H), 5.07 (br, 1H), 4.43 (s, 2H), 4.39 (t, *J* = 7.1 Hz, 1H), 4.17 (t, *J* = 7.7 Hz, 1H), 3.63 (s, 3H), 3.05 (q, *J* = 6.5 Hz, 2H), 2.16 (m, 2H), 1.97 (m, 1H), 1.53-1.38 (m, 4H), 1.30 (d, *J* = 7.0 Hz, 3H), 1.24 (m, 2H), 0.85 (dd, *J* = 6.8 Hz, 6H). MS (ESI) m/z: 519.28 [M+H]^+^; 541.27 [M+Na]^+^.

*Methyl (6S,9S,Z)-1-amino-6-((4-(hydroxymethyl)phenyl)carbamoyl)-9-isopropyl-1,8,11,18-tetraoxo-2,7,10,17-tetraazahenicos-19-en-21-oate (compound* ***10c****)*

Compound **5b** (1.11 g, 2.94 mmol) and compound **9b** (1.0 g, 2.94 mmol) were dissolved in anhydrous DMF (30 mL), and the mixture stirred at RT overnight. After completion of the reaction, the mixture was evaporated under reduced pressure to give the crude product. The obtained crude product was further purified by silica column chromatography (DCM/MeOH = 10:1) to give compound **10c** as a light yellow solid (1.19 g, 41% yield). ^1^H-NMR (400 MHz, DMSO-*d6*): δ 9.92 (s, 1H), 8.20 (t, *J* = 5.2 Hz, 1H), 8.09 (d, *J* = 7.3 Hz, 1H), 7.85 (d, *J* = 8.7 Hz, 1H), 7.54 (d, *J* = 8.4 Hz, 2H), 7.22 (d, *J* = 8.7 Hz, 2H), 6.27 (d, *J* = 12.6 Hz, 1H), 6.23 (d, *J* = 12.6 Hz, 1H), 5.99 (br, 1H), 5.43 (s, 2H), 5.11 (t, *J* = 5.6 Hz, 1H), 4.42 (d, *J* = 5.3 Hz, 2H), 4.37 (m, 1H), 4.19 (t, *J* = 7.7 Hz, 1H), 3.63 (s, 3H), 3.05 (q, *J* = 6.7 Hz, 2H), 2.95 (m, 2H), 2.17 (m, 2H), 1.95 (m, 1H), 1.74-1.55 (m, 2H), 1.51-1.48 (m, 2H), 1.45-1.37 (m, 4H), 1.24 (m, 2H), 0.85 (dd, *J* = 6.7 Hz, 6H). MS (ESI) m/z: 605.7 [M+H]^+^; 627.7 [M+Na]^+^.

*Methyl (Z)-4-((3-(((S)-3-methyl-1-(((S)-1-((4-((((4-nitrophenoxy)carbonyl)oxy)methyl)phenyl)amino)-1-oxopropan-2-yl)amino)-1-oxobutan-2-yl)amino)-3-oxopropyl)amino)-4-oxobut-2-enoate (compound* ***11a****)*

Compound **10a** (0.42 g, 0.88 mmol), bis(4-nitrophenyl) carbonate (0.54 g, 1.76 mmol) and DIPEA (230 μL, 1.32 mmol) were dissolved in DMF (15 mL), and then the mixture was stirred at RT for 7 h, and evaporated under reduced pressure to give the crude product. The crude product was suspended in ether, and purification was performed by ultrasound and filtration 3 times to give compound **11a** as a light yellow solid (0.31 g, 55% yield). ^1^H-NMR (400 MHz, DMSO-*d6*): δ 10.01 (s, 1H), 8.31 (d, *J* = 9.2 Hz, 2H), 8.24 (m, 1H), 7.97 (d, *J* = 8.4 Hz, 1H), 7.64 (d, *J* = 8.7 Hz, 2H), 7.57 (d, *J* = 9.3 Hz, 2H), 7.41 (d, *J* = 8.7 Hz, 2H), 6.27 (d, *J* = 12.6 Hz, 1H), 6.23 (d, *J* = 12.6 Hz, 1H), 5.24 (s, 2H), 4.39 (m, 1H), 4.19 (t, *J* = 15.1 Hz, 1H), 3.64 (s, 3H), 3.28 (q, *J* = 7.0 Hz, 2H), 2.39 (t, *J* = 7.2 Hz, 2H), 1.96 (m, 1H), 1.31 (d, *J* = 7.0 Hz, 3H), 0.86 (dd, *J* = 6.7 Hz, 6H). MS (ESI) m/z: 642.4 [M+H]^+^; 664.5 [M+Na]^+^; 680.4 [M+K]^+^.

*Methyl (Z)-4-((6-(((S)-3-methyl-1-(((S)-1-((4-((((4-nitrophenoxy)carbonyl)oxy)methyl)phenyl)amino)-1-oxopropan-2-yl)amino)-1-oxobutan-2-yl)amino)-6-oxohexyl)amino)-4-oxobut-2-enoate (compound* ***11b****)*

Compound **11b** was synthesized from compound **10b** using a similar preparation method as that for compound **11a**. The yield of the light yellow solid was 86%. ^1^H-NMR (400 MHz, DMSO-*d6*): δ 10.03 (s, 1H), 8.31 (d, *J* = 9.3 Hz, 2H), 8.20 (m, 1H), 7.84 (d, *J* = 6.7 Hz, 1H), 7.64 (d, *J* = 8.4 Hz, 2H), 7.57 (d, *J* = 9.0 Hz, 2H), 7.41 (d, *J* = 8.4 Hz, 2H), 6.28 (d, *J* = 12.6 Hz, 1H), 6.23 (d, *J* = 12.6 Hz, 1H), 5.24 (s, 2H), 4.38 (t, *J* = 7.0 Hz, 1H), 4.18 (t, *J* = 7.6 Hz, 1H), 3.62 (s, 3H), 3.05 (q, *J* = 6.3 Hz, 2H), 2.15 (m, 2H), 1.96 (m, 1H), 1.49 (m, 2H), 1.40 (m, 2H), 1.31 (d, *J* = 7.0 Hz, 3H), 1.25 (m, 2H), 0.86 (m, 6H). MS (ESI) m/z: 684.30 [M+H]^+^; 706.28 [M+Na]^+^.

*Methyl (6S,9S,Z)-1-amino-9-isopropyl-6-((4-((((4-nitrophenoxy)carbonyl)oxy)methyl)phenyl)carbamoyl)-1,8,11,18-tetraoxo-2,7,10,17-tetraazahenicos-19-en-21-oate (compound* ***11c****)*

Compound **11c** was synthesized from compound **10c** using a similar preparation method as that for compound **11a**. The yield of the light yellow solid was 85%. ^1^H-NMR (400 MHz, DMSO-d6): δ 10.08 (s, 1H), 8.31 (d, *J* = 9.0 Hz, 2H), 8.20 (t, *J* = 5.2 Hz, 1H), 8.13 (d, *J* = 7.3 Hz, 2H), 7.83 (d, *J* = 8.4 Hz, 1H), 7.65 (d, *J* = 8.4 Hz, 2H), 7.57 (d, *J* = 9.24 Hz, 2H), 7.41 (d, *J* = 8.4 Hz, 2H), 6.28 (d, *J* = 12.6 Hz, 1H), 6.23 (d, *J* = 12.6 Hz, 1H), 5.99 (s, 1H), 5.43 (s, 2H), 5.24 (s, 2H), 4.38 (t, *J* = 6.8 Hz, 1H), 4.19 (t, *J* = 7.6 Hz, 1H), 3.63 (s, 3H), 3.08-2.92 (m, 4H), 2.17 (m, 2H), 1.71 (m, 1H), 1.60 (m, 2H), 1.52-1.37 (m, 6H), 1.25 (m, 2H), 0.85 (m, 6H). MS (ESI) m/z: 770.7 [M+H]^+^; 792.6 [M+Na]^+^.

*Methyl (Z)-4-((3-(((S)-1-(((S)-1-((4-((5S,8S,11S,12R)-11-((S)-sec-butyl)-12-(2-((S)-2-((1R,2R)-3-(((1S,2R)-1-hydroxy-1-phenylpropan-2-yl)amino)-1-methoxy-2-methyl-3-oxopropyl)pyrrolidin-1-yl)-2-oxoethyl)-5,8-diisopropyl-4,10-dimethyl-3,6,9-trioxo-2,13-dioxa-4,7,10-triazatetradecyl)phenyl)amino)-1-oxopropan-2-yl)amino)-3-methyl-1-oxobutan-2-yl)amino)-3-oxopropyl)amino)-4-oxobut-2-enoate (compound* ***12a****)*

DIPEA (12 μL, 0.0669 mmol) was added to a solution of compound **11a** (33.67 mg, 0.0613 mmol), MMAE (40 mg, 0.0557 mmol), HOBt (9.03 mg, 0.0669 mmol) in DMF (3 mL). The mixture was stirred at RT overnight and then poured into water (20 mL). The mixture was extracted with EA (20 mL × 3) and the organic layers were combined, washed with brine, dried over anhydrous Na_2_SO_4_, and evaporated under reduced pressure to give the crude product. Purification was performed by silica column chromatography (DCM/MeOH = 10:1) to give compound **12a** as a white solid (47.6 mg, 72% yield). HR-MS (ESI) m/z: 1220.7181 [M+H]^+^; 1242.7023 [M+Na]^+^.

*Methyl (Z)-4-((6-(((S)-1-(((S)-1-((4-((5S,8S,11S,12R)-11-((S)-sec-butyl)-12-(2-((S)-2-((1R,2R)-3-(((1S,2R)-1-hydroxy-1-phenylpropan-2-yl)amino)-1-methoxy-2-methyl-3-oxopropyl)pyrrolidin-1-yl)-2-oxoethyl)-5,8-diisopropyl-4,10-dimethyl-3,6,9-trioxo-2,13-dioxa-4,7,10-triazatetradecyl)phenyl)amino)-1-oxopropan-2-yl)amino)-3-methyl-1-oxobutan-2-yl)amino)-6-oxohexyl)amino)-4-oxobut-2-enoate (compound* ***12b****)*

Compound **12b** was synthesized from compound **11b** using a similar preparation method as that for compound **12a**. The yield of the white solid was 75%. HR-MS (ESI) m/z: 1262.7648 [M+H]^+^; 1284.7490 [M+Na]^+^.

*Methyl (6S,9S,Z)-1-amino-6-((4-((5S,8S,11S,12R)-11-((S)-sec-butyl)-12-(2-((S)-2-((1R,2R)-3-(((1S,2R)-1-hydroxy-1-phenylpropan-2-yl)amino)-1-methoxy-2-methyl-3-oxopropyl)pyrrolidin-1-yl)-2-oxoethyl)-5,8-diisopropyl-4,10-dimethyl-3,6,9-trioxo-2,13-dioxa-4,7,10-triazatetradecyl)phenyl)carbamoyl)-9-isopropyl-1,8,11,18-tetraoxo-2,7,10,17-tetraazahenicos-19-en-21-oate (compound* ***12c****)*

Compound **12c** was synthesized from compound **11c** using a similar preparation method as that for compound **12a**. The yield of the white solid was 68%. HR-MS (ESI) m/z: 1348.8118 [M+H]^+^; 1370.7935 [M+Na]^+^.

*(Z)-4-((6-(((S)-1-(((S)-1-((4-(hydroxymethyl)phenyl)amino)-1-oxopropan-2-yl)amino)-3-methyl-1-oxobutan-2-yl)amino)-6-oxohexyl)amino)-4-oxobut-2-enoic acid (compound* ***S1****)*

Compound **4b** (600 mg, 1.84 mmol) and compound **9a** (540 mg, 1.84 mmol) were dissolved in anhydrous DMF (15 mL), and the mixture was stirred at room temperature (RT) for 5 h. After completion of the reaction, the mixture was evaporated under reduced pressure to give the crude product. Purification was performed by silica column chromatography (DCM/MeOH = 10:1) to give compound **S1** as a white solid (720 mg, 78% yield). ^1^H-NMR (400 MHz, DMSO-*d6*) δ 9.87 (s, 1H), 9.43 (br, 1H), 8.17 (d, *J* = 7.0 Hz, 1H), 7.85 (d, *J* = 8.7 Hz, 1H), 7.53 (d, *J* = 8.7 Hz, 2H), 7.23 (d, *J* = 7.0 Hz, 2H), 6.31 (d, *J* = 12.6 Hz, 1H), 6.21 (d, *J* = 12.6 Hz, 1H), 5.11 (br, 1H), 4.42 (s, 1H), 4.38 (m, *J* = 7.0 Hz, 1H), 4.17 (t, *J* = 7.7 Hz, 1H), 3.15 (q, *J* = 6.5 Hz, 2H), 2.17 (m, 2H), 1.97 (m, 1H), 1.48 (m, 4H), 1.31-1.24 (m, 5H), 0.86 (dd, 6H). MS (ESI) m/z: 505.4 [M+H]^+^; 527.9 [M+Na]^+^; 503.4 [M-H]^-^.

*(6S,9S,Z)-1-amino-6-((4-(hydroxymethyl)phenyl)carbamoyl)-9-isopropyl-1,8,11,18-tetraoxo-2,7,10,17-tetraazahenicos-19-en-21-oic acid (compound* ***S2****)*

Compound **S2** was synthesized from compound **4b** and **9b** using a similar preparation method as that for compound **S1**. The yield of the light yellow solid was 86%. ^1^H-NMR (400 MHz, DMSO-*d6*) δ 11.60 (br, 1H), 9.91 (s, 1H), 9.62 (br, 1H), 8.11 (d, *J* = 7.6 Hz, 1H), 7.88 (d, *J* = 8.4 Hz, 1H), 7.54 (d, *J* = 8.4 Hz, 2H), 7.22 (d, *J* = 7.6 Hz, 2H), 6.21 (d, *J* = 7.6 Hz, 1H), 6.05 (d, *J* = 6.0 Hz, 1H), 5.45 (d, 2H), 5.11 (br, 1H), 4.42 (s, 1H), 4.37 (m, 1H), 4.18 (t, *J* = 8.4 Hz, 1H), 3.14 (q, 2H), 2.98 (m, 2H), 2.17 (m, 2H), 1.97 (m, 1H), 1.70 (m, 1H), 1.59 (m, 1H), 1.52-1.34 (m, 6H), 1.27 (m, 2H), 0.83 (dd, 6H). MS (ESI) m/z: 591.4 [M+H]^+^; 613.4 [M+Na]^+^; 589.5 [M-H]^-^.

*(Z)-4-((6-(((S)-3-methyl-1-(((S)-1-((4-((((4-nitrophenoxy)carbonyl)oxy)methyl)phenyl)amino)-1-oxopropan-2-yl)amino)-1-oxobutan-2-yl)amino)-6-oxohexyl)amino)-4-oxobut-2-enoic acid (compound* ***S3****)*

Compound **S1** (300 mg, 0.6 mmol), bis(4-nitrophenyl) carbonate (360 mg, 1.2 mmol) and DIPEA (104 mg, 0.9 mmol) were dissolved in DMF (15 mL), and then the mixture was stirred at RT for 10 h. After completion of the reaction, the mixture was evaporated under reduced pressure to give the crude product. The crude product was suspended in ether and purification was performed by ultrasound and filtration 3 times to give compound **11a** as a white solid (0.31 g, 55% yield). ^1^H-NMR (400 MHz, DMSO-*d6*) δ 15.22 (br, 1H), 10.03 (s, 1H), 9.18 (br, 1H), 8.31 (d, *J* = 9.2 Hz, 2H), 8.21 (d, *J* = 6.7 Hz, 1H), 7.84 (d, *J* = 8.7 Hz, 1H), 7.64 (d, *J* = 8.4 Hz, 2H), 7.57 (d, *J* = 9.2 Hz, 2H), 7.41 (d, *J* = 8.4 Hz, 2H), 6.37 (d, *J* = 12.6 Hz, 1H), 6.23 (d, *J* = 12.6 Hz, 1H), 5.24 (s, 2H), 4.40 (m, 1H), 4.18 (t, *J* = 7.8 Hz, 1H), 3.15 (q, *J* = 6.4 Hz, 2H), 2.16 (m, 2H), 1.96 (m, 1H), 1.47 (m, 4H), 1.32-1.24 (m, 5H), 0.85 (dd, *J* = 6.4 Hz, 6H). MS (ESI) m/z: 670.4 [M+H]^+^; 692.4 [M+Na]^+^; 668.4 [M+K]^+^.

*(6S,9S,Z)-1-amino-9-isopropyl-6-((4-((((4-nitrophenoxy)carbonyl)oxy)methyl)phenyl)carbamoyl)-1,8,11,18-tetraoxo-2,7,10,17-tetraazahenicos-19-en-21-oic acid (compound* ***S4****)*

Compound **S4** was synthesized from compound **S2** using a similar preparation method as that for compound **S3**. The yield of the light yellow solid was 16%. ^1^H-NMR (400 MHz, DMSO-*d6*) δ 15.22 (br, 1H), 10.10 (s, 1H), 9.30 (br, 1H), 8.31 (d, *J* = 9.0 Hz, 2H), 8.15 (d, *J* = 7.6 Hz, 1H), 7.86 (d, *J* = 8.4 Hz, 1H), 7.65 (d, *J* = 8.7 Hz, 2H), 7.57 (d, *J* = 9.2 Hz, 2H), 7.41 (d, *J* = 8.7 Hz, 2H), 7.22 (d, *J* = 8.7 Hz, 1H), 6.36 (d, *J* = 12.6 Hz, 1H), 6.22 (d, *J* = 12.6 Hz, 1H), 6.03 (s, 2H), 5.46 (s, 2H), 5.24 (s, 2H), 4.41 (m, 1H), 4.20 (t, *J* = 5.6 Hz, 1H), 3.15 (q, *J* = 7.7 Hz, 2H), 2.98 (m, 2H), 2.16 (m, 2H), 1.97 (m, 1H), 1.69 (m, 1H), 1.60 (m, 1H), 1.48 (m, 6H), 1.27 (m, 2H), 0.85 (m, 6H). MS (ESI) m/z: 756.7 [M+H]^+^; 778.4 [M+Na]^+^; 754.6 [M-H]^-^.

*(Z)-4-((6-(((S)-1-(((S)-1-((4-((5S,8S,11S,12R)-11-((S)-sec-butyl)-12-(2-((S)-2-((1R,2R)-3-(((1S,2R)-1-hydroxy-1-phenylpropan-2-yl)amino)-1-methoxy-2-methyl-3-oxopropyl)pyrrolidin-1-yl)-2-oxoethyl)-5,8-diisopropyl-4,10-dimethyl-3,6,9-trioxo-2,13-dioxa-4,7,10-triazatetradecyl)phenyl)amino)-1-oxopropan-2-yl)amino)-3-methyl-1-oxobutan-2-yl)amino)-6-oxohexyl)amino)-4-oxobut-2-enoic acid (compound* ***S5****)*

DIPEA (10.68 μL) was added to a solution of compound **S3** (41 mg, 0.06 mmol), MMAE (40 mg, 0.06 mmol), HOBt (8.28 mg, 0.06 mmol) in DMF (3 mL). The mixture was stirred at RT for 18 h, and then evaporated under reduced pressure to give the crude product. Purification was performed by silica column chromatography (DCM/MeOH = 20:1) to give compound **S5** as a white solid (39.4 mg, 57% yield). HR-MS (ESI) m/z: 1248.7497 [M+H]^+^; 1270.7308 [M+Na]^+^.

*(6S,9S,Z)-1-amino-6-((4-((5S,8S,11S,12R)-11-((S)-sec-butyl)-12-(2-((S)-2-((1R,2R)-3-(((1S,2R)-1-hydroxy-1-phenylpropan-2-yl)amino)-1-methoxy-2-methyl-3-oxopropyl)pyrrolidin-1-yl)-2-oxoethyl)-5,8-diisopropyl-4,10-dimethyl-3,6,9-trioxo-2,13-dioxa-4,7,10-triazatetradecyl)phenyl)carbamoyl)-9-isopropyl-1,8,11,18-tetraoxo-2,7,10,17-tetraazahenicos-19-en-21-oic acid (compound* ***S6****)*

Compound **S6** was synthesized from compound **S4** using a similar preparation method as that for compound **S5**. The yield of the white solid was 57%. HR-MS (ESI) m/z: 1334.7972 [M+H]^+^; 1356.7790 [M+Na]^+^.

*(E)-6-(4-methoxy-4-oxobut-2-enamido)hexanoic acid (compound* ***S7****)*

To monomethyl fumarate (2.0 g, 15.37 mmol) in DCM (50 mL) was added *N*-methylmorpholine (2.54 mL, 23.06 mM). After cooling the solution to -20 °C, isobutyl chloroformate (2.0 mL) was added dropwise and continue stirring for 3 h. Then, added a solution of 6-aminocaproic acid (2.02 g, 15.37 mM) in DMF (10 mL) and continue stirring at -20 °C for another 1 h. After completion of the reaction, the mixture was evaporated under reduced pressure to give the crude product as an orange oil. The obtained residue was redissolved in water, and the pH was adjusted to approximate 1 with concentrated hydrochloric acid to precipitate solid insoluble matter. The insoluble matter was separated by suction filtration, and the filter cake was further dried to obtain a light pink solid (3.33 g, 89% yield). ^1^H-NMR (400 MHz, DMSO-*d6*) δ 12.02 (s, 1H), 8.54 (t, *J* = 5.46 Hz, 1H), 7.00 (d, *J* = 15.68 Hz, 1H), 6.57 (d, *J* = 15.68 Hz, 1H), 3.72 (s, 3H), 3.15 (m, 2H), 2.19 (t, *J* = 7.42 Hz, 2H), 1.49 (m, 2H), 1.43 (m, 2H), 1.27 (m, 2H). MS (ESI) m/z: 242.10 [M-H]^-^.

*Methyl (E)-4-((6-(((S)-1-(((S)-1-((4-(hydroxymethyl)phenyl)amino)-1-oxopropan-2-yl)amino)-3-methyl-1-oxobutan-2-yl)amino)-6-oxohexyl)amino)-4-oxobut-2-enoate (compound* ***S8****)*

To a solution of **S7** (0.61 g, 2.5 mmol) in DCM (8 mL) was added EDCI (0.71 g, 3.7 mM), HOBt (0.5 g, 3.7 mM), and DIPEA (0.61 mL, 3.7 mM). The mixture was stirred at RT for 1 h, then, a solution of **9a** (0.87 g, 3.0 mM) in DMF (12 mL) was added, and the reaction was continued to stir at RT for another 1h. After completion of the reaction, the mixture was evaporated under reduced pressure to give the crude product. The obtained crude product was further purified by silica column chromatography (DCM/MeOH = 10:1) to give compound **S8** as a white solid (1.05 g, 81% yield). ^1^H-NMR (400 MHz, DMSO-*d6*) δ 9.91 (s, 1H), 8.56 (t, *J* = 5.62 Hz, 1H), 8.19 (d, 1H), 7.86 (d, 1H), 7.54 (d, 2H), 7.23 (d, 2H), 7.01 (d, *J* = 15.42 Hz, 1H), 6.56 (d, *J* = 15.42 Hz, 1H), 5.13 (br, 1H), 4.42 (s, 2H), 4.38 (m, 1H), 4.17 (q, 1H), 3.72 (s, 3H), 3.12 (m, 2H), 2.16 (m, 2H), 1.96 (m, 1H), 1.43 (m, 4H), 1.31 (s, 2H), 1.30 (s, 3H), 0.85 (dd, *J* = 6.72 Hz, 6H). MS (ESI) m/z: 541.26 [M+H]^+^。

*Methyl (6S,9S,E)-1-amino-6-((4-(hydroxymethyl)phenyl)carbamoyl)-9-isopropyl-1,8,11,18-tetraoxo-2,7,10,17-tetraazahenicos-19-en-21-oate (compound* ***S9****)*

Compound **S9** was synthesized from compound **S7** and **9b** using a similar preparation method as that for compound **S8**. The yield of the white solid was 39%. ^1^H-NMR (400 MHz, DMSO-*d6*) δ 9.93 (s, 1H), 8.54 (t, *J* = 5.64 Hz, 1H), 8.07 (d, 1H), 7.83 (d, 1H), 7.55 (d, 2H), 7.22 (d, 2H), 7.01 (d, *J* = 15.4 Hz, 1H), 6.56 (d, *J* = 15.4 Hz, 1H), 6.03 (t, 1H), 5.42 (s, 2H), 5.10 (t, 1H), 4.42 (d, 2H), 4.37 (m, 1H), 4.19 (m, 1H), 3.72 (s, 3H), 3.15 (m, 2H), 2.99 (m, 2H), 2.16 (m, 2H), 1.98 (m, 1H), 1.66 (m, 2H), 1.44 (m, 6H), 1.25 (m, 2H), 0.84 (dd, *J* = 6.68 Hz, 6H). MS (ESI) m/z: 605.5 [M+H]^+^; 627.5 [M+Na]^+^.

*Methyl (E)-4-((6-(((S)-3-methyl-1-(((S)-1-((4-((((4-nitrophenoxy)carbonyl)oxy)methyl)phenyl)amino)-1-oxopropan-2-yl)amino)-1-oxobutan-2-yl)amino)-6-oxohexyl)amino)-4-oxobut-2-enoate (compound* ***S10****)*

Compound **S10** was synthesized from compound **S8** using a similar preparation method as that for compound **S3**. The yield of the white solid was 75%. ^1^H-NMR (400 MHz, DMSO-*d6*) δ 10.04 (s, 1H), 8.53 (br, 1H), 8.31 (d, 2H), 8.22 (d, 1H), 7.85 (d, 1H), 7.64 (d, 2H), 7.57 (d, 2H), 7.41 (d, 2H), 7.01 (d, *J* = 15.44 Hz, 1H), 6.56 (d, *J* = 15.44 Hz, 1H), 5.24 (s, 2H), 4.39 (t, 1H), 4.18 (t, 1H), 3.71 (s, 3H), 3.13 (q, 2H), 2.16 (m, 2H), 1.95 (m, 1H), 1.49 (br, 2H), 1.43 (t, 2H), 1.31 (d, 3H), 1.24 (m, 2H), 0.85 (dd, 6H). MS (ESI) m/z: 684.29 [M+H]^+^; 706.27 [M+Na]^+^; 722.24 [M+K]^+^.

*Methyl (6S,9S,E)-1-amino-9-isopropyl-6-((4-((((4-nitrophenoxy)carbonyl)oxy)methyl)phenyl)carbamoyl)-1,8,11,18-tetraoxo-2,7,10,17-tetraazahenicos-19-en-21-oate (compound* ***S11****)*

Compound **S11** was synthesized from compound **S9** using a similar preparation method as that for compound **S3**. The yield of the light yellow solid was 71%. ^1^H-NMR (400 MHz, DMSO-*d6*) δ 10.09 (s, 1H), 8.53 (br, 1H), 8.32 (d, 2H), 8.15 (d, 1H), 7.84 (d, 1H), 7.65 (d, 2H), 7.57 (d, 2H), 7.41 (d, 2H), 6.98 (d, *J* = 15.68 Hz, 1H), 6.56 (d, *J* = 15.68 Hz, 1H), 5.99 (br, 1H), 5.45 (s, 2H), 5.24 (s, 2H), 4.38 (br, 1H), 4.19 (t, 1H), 3.71 (s, 3H), 3.13 (d, 2H), 3.03 (m, 1H), 2.93 (m, 1H), 2.16 (m, 2H), 1.99 (s, 1H), 1.93 (m, 1H), 1.69 (m, 1H), 1.60 (m, 1H), 1.49 (m, 1H), 1.43 (t, 2H), 1.25 (m, 4H), 0.84 (dd, 6H). MS (ESI) m/z: 770.6 [M+H]^+^; 792.4 [M+Na]^+^.

*Methyl (E)-4-((6-(((S)-1-(((S)-1-((4-((5S,8S,11S,12R)-11-((S)-sec-butyl)-12-(2-((S)-2-((1R,2R)-3-(((1S,2R)-1-hydroxy-1-phenylpropan-2-yl)amino)-1-methoxy-2-methyl-3-oxopropyl)pyrrolidin-1-yl)-2-oxoethyl)-5,8-diisopropyl-4,10-dimethyl-3,6,9-trioxo-2,13-dioxa-4,7,10-triazatetradecyl)phenyl)amino)-1-oxopropan-2-yl)amino)-3-methyl-1-oxobutan-2-yl)amino)-6-oxohexyl)amino)-4-oxobut-2-enoate (compound* ***S12****)*

Compound **S12** was synthesized from compound **S10** using a similar preparation method as that for compound **S5**. The yield of the light yellow solid was 71%. ^1^H-NMR (400 MHz, CDCl_3_) δ 9.21 (br, 1H), 7.52 (t, 4H), 7.31 (m, 5H), 7.21 (m, 3H), 6.84 (dd, 1H), 6.74 (m, 1H), 6.56 (m, 0.5H), 5.06 (m, 2H), 4.91 (s, 1H), 4.73 (m, 3H), 4.46 (t, 1H), 4.23 (m, 1H), 4.12 (m, 1H), 4.02 (m, 1H), 3.79 (s, 4H), 3.46 (m, 1H), 3.36 (s, 5H), 3.25 (d, 4H), 3.12 (d, 1H), 2.99 (s, 2H), 2.88 (d, 3H), 2.73 (d, 0.5H), 2.43 (m, 2H), 2.28 (s, 2H), 2.17 (m, 1H), 2.02 (m, 3H), 1.81 (s, 2H), 1.63 (m, 3H), 1.51 (m, 2H), 1.38 (d, 2H), 1.30 (t, 3H), 1.21 (d, 4H), 1.01 (m, 8H), 0.79 (m, 24H). HR-MS (ESI) m/z: 1262.7641 [M+H]^+^; 1284.7491 [M+Na]^+^.

*Methyl (6S,9S,E)-1-amino-6-((4-((5S,8S,11S,12R)-11-((S)-sec-butyl)-12-(2-((S)-2-((1R,2R)-3-(((1S,2R)-1-hydroxy-1-phenylpropan-2-yl)amino)-1-methoxy-2-methyl-3-oxopropyl)pyrrolidin-1-yl)-2-oxoethyl)-5,8-diisopropyl-4,10-dimethyl-3,6,9-trioxo-2,13-dioxa-4,7,10-triazatetradecyl)phenyl)carbamoyl)-9-isopropyl-1,8,11,18-tetraoxo-2,7,10,17-tetraazahenicos-19-en-21-oate (compound* ***S13****)*

Compound **S13** was synthesized from compound **S11** using a similar preparation method as that for compound **S5**. The yield of the light yellow solid was 56%. ^1^H-NMR (400 MHz, CDCl_3_) δ 10.01 (br, 1H), 8.54 (t, 1H), 8.13 (d, 1H), 7.92 (d, 0.5H), 7.84 (d, 1H), 7.67 (d, 0.5H), 7.58 (d, 2H), 7.30 (m, 6H), 7.16 (m, 1H), 7.01 (d, *J* = 15.72 Hz, 1H), 6.57 (d, *J* = 15.72 Hz, 1H), 6.00 (br, 1H), 5.45 (br, 2H), 5.37 (d, 0.5H), 5.01 (m, 2H), 4.74 (m, 0.5H), 4.64 (m, 0.5H), 4.42 (m, 3H), 4.23 (m, 2H), 3.98 (m, 2H), 3.71 (s, 3H), 2.91 (m, 8H), 2.41 (d, 1H), 2.21 (m, 6H), 1.98 (m, 4H), 1.74 (m, 4H), 1.49 (m, 8H), 1.26 (m, 6H), 1.02 (m, 9H), 0.91 (m, 4H), 0.82 (m, 24H). HR-MS (ESI) m/z: 1348.8121 [M+H]^+^; 1370.7944 [M+Na]^+^.

*(Z)-4-oxo-4-((6-oxo-6-(phenylamino)hexyl)amino)but-2-enoic acid (compound* ***S14****)*

The solution of **3b** (3.0 g, 13.1 mM) in THF (50 mL) was cooled to -20 °C, and then the *N*-methylmorpholine (1.59 m, 15.7 mM) and isobutyl chloroformate (2.14 g, 15.7 mM) were added dropwise. After stirring the reaction at -20 °C for 20 min, aniline (1.83 g, 19.6 mM) was added and stirred for another 2 h. After completion of the reaction, dilute hydrochloric acid was added and extracted with ethyl acetate. The organic phases were combined, washed with saturated brine, and concentrated to obtain a residue. The obtained crude product was further purified by silica column chromatography (DCM/MeOH = 5:1) to give compound **S14** as a white solid (3.2 g, 81% yield). ^1^H-NMR (400 MHz, DMSO-*d6*) δ 11.98 (br, 1H), 11.35 (s, 1H), 8.68 (t, 1H), 7.61 (d, *J* = 7.6 Hz, 2H), 7.32 (t, *J* = 7.8 Hz, 2H), 7.07 (t, *J* = 7.4 Hz, 2H), 6.26 (dd, 2H), 3.12 (q, 2H), 1.54-1.42 (m, 4H), 1.30 (m, 2H). MS (ESI) m/z: 303.14 [M-H]^-^.

*Methyl (Z)-4-oxo-4-((6-oxo-6-(phenylamino)hexyl)amino)but-2-enoate (compound* ***S15****)*

To a solution of **S14** (0.33 g, 1.1 mmol) in DMF (8 mL) was added K_2_CO_3_ (0.23 g, 1.63 mM) and methyl iodide (0.23 mL, 1.63 mM). The mixture was stirred at RT for 4 h, and then, the mixture was filtered to remove insoluble salts and evaporated under reduced pressure to give the crude product. The obtained crude product was further purified by silica column chromatography (PE:EA = 1:1) to give compound **S15** as a white solid (0.32 g, 93% yield). ^1^H-NMR (400 MHz, DMSO-*d6*) δ 11.33 (s, 1H), 8.67 (t, 1H), 7.61 (d, *J* = 7.6 Hz, 2H), 7.32 (t, *J* = 7.8 Hz, 2H), 7.07 (t, *J* = 7.4 Hz, 2H), 6.26 (dd, 2H), 3.57 (s, 3H), 3.12 (q, 2H), 2.29 (t, 2H), 1.57-1.41 (m, 4H), 1.30 (m, 2H). MS (ESI) m/z: 319.16 [M+H]^+^; 341.14 [M+Na]^+^.

*Methyl 3-((2-hydroxyethyl)thio)-4-oxo-4-((6-oxo-6-(phenylamino)hexyl)amino)butanoate (compound* ***S16****)*

To a solution of **S15** (0.15 g, 0.47 mM) in methanol (5 mL) was added 2-hydroxy-1-ethanethiol (52 μL, 0.7 mM). The mixture was reacted at RT for 30 minutes, then, the mixture was evaporated under reduced pressure to give the crude product. The obtained crude product was further purified by silica column chromatography (DCM:MeOH = 20:1) to give compound **S16** as a white solid (0.20 g, 80% yield). ^1^H-NMR (400 MHz, DMSO-*d6*) δ 10.00 (s, 1H), 8.09 (d, *J* = 5.74 Hz, 2H), 7.56 (d, *J* = 7.56 Hz, 2H), 7.28 (t, *J* = 7.98 Hz, 2H), 7.01 (t, *J* = 7.42 Hz, 1H), 4.83 (br, 1H), 3.70 (t, *J* = 7.58 Hz, 1H), 3.57 (s, 3H), 3.51 (t, *J* = 7.0 Hz, 2H), 3.02 (m, 2H), 2.80 (m, 1H), 2.67 (m, 3H), 2.25 (t, *J* = 7.44 Hz, 2H), 1.49 (m, 2H), 1.38 (m, 2H), 1.25 (m, 2H). MS (ESI) m/z: 397.18 [M+H]^+^; 419.16 [M+Na]^+^.

*Methyl 3-(benzylthio)-4-oxo-4-((6-oxo-6-(phenylamino)hexyl)amino)butanoate(compound* ***S17****)*

To a solution of **S15** (0.15 g, 0.47 mM) in methanol (5 mL) was added benzyl mercaptan (164 μL, 1.41 mM). The mixture was reacted at RT for 30 minutes, and then, evaporated under reduced pressure to give the crude product. The obtained crude product was further purified by silica column chromatography (PE:EA = 1:1) to give compound **S17** as a white solid (0.17 g, 82% yield). ^1^H-NMR (400 MHz, CDCl_3_) δ 10.02 (s, 1H), 8.09 (t, *J* = 5.62 Hz, 2H), 7.57 (d, *J* = 7.56 Hz, 2H), 7.32-7.22 (m, 7H), 7.02 (t, *J* = 7.44 Hz, 1H), 3.86 (m, 2H), 3.76 (t, *J* = 7.58 Hz, 1H), 3.56 (s, 3H), 3.05 (m, 2H), 2.91 (m, 1H), 2.67 (m, 1H), 2.24 (m, 2H), 1.49 (m, 2H), 1.38 (m, 2H), 1.25 (m, 2H). MS (ESI) m/z: 443.22 [M+H]^+^; 465.20 [M+Na]^+^.

*Methyl 3-(((R)-2-acetamido-3-(benzylamino)-3-oxopropyl)thio)-4-oxo-4-((6-oxo-6-(phenylamino)hexyl)amino)butanoate (compound* ***S18****)*

To a solution of **S15** (0.15 g, 0.47 mM) in methanol (5 mL) was added (*R*)-2-acetamido-*N*-benzyl-3-mercaptopropanamide (100 mg, 0.38 mM). The mixture was reacted at RT for 30 minutes, and then, evaporated under reduced pressure to give the crude product. The obtained crude product was further purified by silica column chromatography (DCM:MeOH = 30:1) to give compound **S18** as a white solid (0.17 g, 82% yield). ^1^H-NMR (400 MHz, DMSO-*d6*) δ 10.00 (s, 1H), 8.53 (t, *J* = 5.88 Hz, 1H), 8.23 (d, *J* = 8.12 Hz, 2H), 7.97 (t, *J* = 5.46 Hz, 1H), 7.55 (d, *J* = 8.12 Hz, 2H), 7.32-7.21 (m, 7H), 7.02 (t, *J* = 7.28 Hz, 1H), 4.49 (q, *J* = 7.37 Hz, 1H), 4.27 (m, 2H), 3.75 (t, *J* = 7.44 Hz, 2H), 3.57 (s, 3H), 3.03 (q, *J* = 6.16 Hz, 2H), 2.98-2.88 (m, 2H), 2.80 (m, 1H), 2.66 (dd, 1H), 2.24 (t, *J* = 7.42 Hz, 2H), 1.87 (s, 3H), 1.48 (m, 2H), 1.38 (m, 2H), 1.23 (m, 2H). MS (ESI) m/z: 571.26 [M+H]^+^; 593.24 [M+Na]^+^.

*tert-butyl (S)-(3-methyl-1-oxo-1-(phenylamino)butan-2-yl)carbamate (compound* ***S20****)*

To a solution of *N*-Boc-Valine (**S19**) in THF (30 mL) was added phenylamine (0.93 g, 10 mM) and DCC (2.39 g, 11 mM). The mixture was reacted at room temperature overnight, then the insoluble substance (DCU) was removed by filtration, and the resulting organic phase was concentrated under reduced pressure to obtain the crude product. The crude product was further purified by silica column chromatography (PE:EA = 5:1) to give compound **S20** as a white solid (2.2 g, 75% yield). ^1^H-NMR (400 MHz, DMSO-*d6*) δ 9.98 (s, 1H), 7.60 (d, *J* = 7.6 Hz, 2H), 7.30 (d, *J* = 8.0 Hz, 2H), 7.04 (t, *J* = 7.4 Hz, 1H), 6.90 (d, *J* = 8.4 Hz, 1H), 3.91 (t, *J* = 7.0 Hz, 1H), 1.96 (m, 1H), 1.39 (s, 9H), 0.88 (d, *J* = 6.7 Hz, 6H). MS (ESI) m/z: 293.1 [M+H]^+^; 315.2 [M+Na]^+^.

*(S)-2-amino-3-methyl-N-phenylbutanamide (compound* ***S21****)*

To a solution of **S20** (5.85 g, 20 mM) in DCM (50 mL) was added TFA (12.5 mL). The mixture was reacted at RT overnight, then the solution was concentrated under reduced pressure to obtain the crude product. The crude product was further purified by silica column chromatography (DCM:MeOH = 50:1) to give compound **S21** as a light yellow oil (2.8 g, 88% yield). ^1^H-NMR (400 MHz, DMSO-*d6*) δ 9.84 (br, 1H), 7.63 (dd, *J* = 8.7 Hz, 2H), 7.30 (d, *J* = 8.0 Hz, 2H), 7.03 (td, *J* = 7.4Hz, 1H), 3.10 (d, *J* = 5.6 Hz, 1H), 1.93 (m, 1H), 0.88 (d, *J* = 6.7 Hz, 6H). MS (ESI) m/z: 193.1 [M+H]^+^; 215.1 [M+Na]^+^.

*tert-butyl methyl((S)-3-methyl-1-(((S)-3-methyl-1-oxo-1-(phenylamino)butan-2-yl)amino)-1-oxobutan-2-yl)carbamate (compound* ***S22****)*

To a solution of *N*-methyl-*N*-Boc-Valine (0.58 g, 2.5 mM) in DCM (30 mL) was added EDCI (0.58 g, 3 mM), HOBt (0.41 g, 3 mM), and DIPEA (0.51 mL, 3.0 mM). After the reaction solution was stirred at room temperature for 1 h, **S21** (0.48 g, 2.5 mM) was then added, and the resulting mixture was continued to stir at room temperature overnight. After the reaction is completed, the solution was concentrated under reduced pressure to obtain the crude product. The crude product was further purified by silica column chromatography (PE:EA = 5:1) to give compound **S22** as a light pink solid (0.43 g, 42% yield). ^1^H-NMR (400 MHz, DMSO-*d6*) δ 10.11 (d, 1H), 7.95 (d, *J* = 8.3 Hz, 1H), 7.58 (d, *J* = 7.6 Hz, 2H), 7.31 (t, *J* = 8.0 Hz, 2H), 7.05 (t, *J* = 7.4 Hz, 1H), 4.23 (t, *J* = 9.4 Hz, 2H), 2.77 (s, 3H), 2.04 (m, 2H), 1.43 (s, 9H), 0.87-0.78 (m, 12H). MS (ESI) m/z: 406.2 [M+H]^+^; 428.3 [M+Na]^+^.

*(S)-3-methyl-N-((S)-3-methyl-1-oxo-1-(phenylamino)butan-2-yl)-2-(methylamino)butanamide (compound* ***S23****)*

To a solution of **S22** (0.42 g, 1.04 mM) in DCM (5 mL) was added TFA (1.25 mL). The mixture was reacted at room temperature for 3 h, then the solution was concentrated under reduced pressure to obtain the crude product. The crude product was redissolved in ethyl acetate, washed twice with saturated sodium bicarbonate, the solvent was removed by concentration under reduced pressure again, and the obtained solid was recrystallized from ethyl acetate to obtain compound **S23** as a white powdery solid (0.31 g, 99% yield). ^1^H-NMR (400 MHz, DMSO-*d6*) δ 10.16 (s, 1H), 8.03 (d, *J* = 9.0 Hz, 1H), 7.59 (d, *J* = 8.7 Hz, 2H), 7.30 (t, *J* = 7.1 Hz, 2H), 7.04 (t, *J* = 7.4 Hz, 1H), 4.39 (t, *J* = 8.0 Hz, 1H), 2.69 (d, *J* = 6.2 Hz, 1H), 2.20 (s, 3H), 2.03 (m, 1H), 1.76 (m, 1H), 0.89 (m, 12H). MS (ESI) m/z: 306.22 [M+H]^+^; 328.20 [M+Na]^+^.

*methyl (6S,9S,Z)-1-amino-9-isopropyl-6-((4-(((methyl((S)-3-methyl-1-(((S)-3-methyl-1-oxo-1-(phenylamino)butan-2-yl)amino)-1-oxobutan-2-yl)carbamoyl)oxy)methyl)phenyl)carbamoyl)-1,8,11,18-tetraoxo-2,7,10,17-tetraazahenicos-19-en-21-oate (compound* ***S24****)*

To a solution of **11c** (254 mg, 0.33 mM) in DMF (10 mL) was added **S23** (100 mg, 0.33 mM), HOBt (44.6 mg, 0.33 mM), and DIPEA (85.3 mg, 0.66 mM). After the reaction solution was stirred at room temperature for 18 h, the solvent was concentrated under reduced pressure, and the obtained crude product was further purified by silica column chromatography (DCM:MeOH = 8:1) to give compound **S24** as a white solid (0.43 g, 86% yield). ^1^H-NMR (400 MHz, DMSO-*d6*) δ 10.07-9.97 (m, 2H), 8.19 (t, *J* = 5.7 Hz, 1H), 8.12 (d, *J* = 7.3Hz, 1H), 7.94 (d, *J* = 7.8 Hz, 1H), 7.83 (d, *J* = 8.4Hz, 2H), 7.60-7.57 (m, 4H), 7.34-7.28 (m, 4H), 7.05 (t, *J* = 7.4 Hz, 1H), 6.27 (d, *J* = 12.6 Hz, 2H), 6.23 (d, *J* = 12.6Hz, 2H), 5.98 (t, *J* = 5.6Hz, 1H), 5.43 (s, 1H), 5.11-5.01 (m, 2H), 4.39 (m, 2H), 4.18 (q, *J* = 8.2Hz, 2H), 3.63 (s, 3H), 3.08-2.95 (m, 4H), 2.85 (s, 3H), 2.17 (m, 2H), 2.09 (s, 1H), 1.97 (m, 1H), 1.69 (m, 1H), 1.61 (m, 1H), 1.53-1.31 (m, 6H), 1.23 (m, 2H), 0.87-0.77 (m, 18H). MS (ESI) m/z: 936.8 [M+H]^+^; 959.1 [M+Na]^+^.

*4-((S)-2-((S)-2-(6-(2,5-dioxo-2,5-dihydro-1H-pyrrol-1-yl)hexanamido)-3-methylbutanamido)-5-ureidopentanamido)benzyl methyl((S)-3-methyl-1-(((S)-3-methyl-1-oxo-1-(phenylamino)butan-2-yl)amino)-1-oxobutan-2-yl)carbamate (compound* ***S26****)*

The compound MC-VC-PAB-PNP (**S25**) was synthesized according to our previously reported method (*Int J Mol Sci*, 2017, 18(9):1860.). To a solution of **S25** (1.0 g, 1.36 mM) in DMF (20 mL) was added **S23** (410 mg, 1.36 mM), HOBt (180 mg, 1.36 mM), and DIPEA 176 mg, 1.36 mM). After the reaction solution was stirred at room temperature overnight, the solvent was concentrated under reduced pressure, and the obtained crude product was further purified by silica column chromatography (DCM/MeOH=10:1) to give compound **S26** as a white solid (0.43 g, 86% yield). ^1^H-NMR (400 MHz, DMSO-*d6*) δ 10.01 (m, 2H), 8.13 (m, 1H), 8.12 (d, *J* = 7.3Hz, 1H), 7.82 (d, 1H), 7.58 (m, 4H), 7.30 (m, 4H), 7.04 (t, 1H), 7.00 (s, 2H), 5.98 (t, *J* = 5.6Hz, 1H), 5.43 (s, 2H), 5.11-5.00 (m, 2H), 4.35 (m, 2H), 4.18 (q, 2H), 3.30 (m, 2H), 3.08-2.91 (m, 2H), 2.85 (s, 3H), 2.17 (m, 3H), 1.99 (m, 2H), 1.71-1.56 (m, 2H), 1.47 (m, 6H), 1.21 (m, 2H), 0.87-0.77 (m, 18H). MS (ESI) m/z: 904.9 [M+H]^+^; 926.9 [M+Na]^+^.

*methyl (6S,9S)-19-(((R)-2-acetamido-3-(benzylamino)-3-oxopropyl)thio)-1-amino-9-isopropyl-6-((4-(((methyl((S)-3-methyl-1-(((S)-3-methyl-1-oxo-1-(phenylamino)butan-2-yl)amino)-1-oxobutan-2-yl)carbamoyl)oxy)methyl)phenyl)carbamoyl)-1,8,11,18-tetraoxo-2,7,10,17-tetraazahenicosan-21-oate (compound* ***13****)*

To a solution of **S24** (300 mg, 0.32 mM) in DMF (10 mL) was added (*R*)-2-acetamido-*N*-benzyl-3-mercaptopropanamide (100 mg, 0.38 mM) and catalytic amount of DIPEA. After the mixture was stirred at room temperature for 30 minutes, the insoluble material was separated by filtration, and the filter cake was washed twice with methanol. The crude product was suspended in ether, and purification was performed by ultrasound and filtration 3 times to give compound **13** as a white solid (210 mg, 55.2% yield). ^1^H-NMR (400 MHz, DMSO-*d6*) δ 10.02 (m, 2H), 8.57 (dt, 1H), 8.27-8.14 (m, 3H), 8.05-7.95 (m, 2H), 7.83 (d, 1H), 7.59 (m, 4H), 7.34-7.21 (m, 9H), 7.04 (t, 1H), 5.98 (t, 1H), 5.44 (s, 2H), 5.05 (m, 2H), 4.49 (m, 1H), 4.39-4.28 (m, 4H), 4.18 (m, 2H), 3.62 (m, 1H), 3.56 (s, 3H), 3.03-2.83 (m, 9H), 2.77-2.59 (m, 2H), 2.21-1.91 (m, 5H), 1.85 (m, 2H), 1.69 (m, 1H), 1.58 (m, 1H), 1.46-1.32 (m, 6H), 1.23 (m, 2H), 0.91-0.77 (m, 18H). MS (ESI) m/z: 1188.61 [M+H]^+^; 1210.59 [M+Na]^+^.

*4-((2S)-2-((2S)-2-(6-(3-(((R)-2-acetamido-3-(benzylamino)-3-oxopropyl)thio)-2,5-dioxopyrrolidin-1-yl)hexanamido)-3-methylbutanamido)-5-ureidopentanamido)benzyl methyl((S)-3-methyl-1-(((S)-3-methyl-1-oxo-1-(phenylamino)butan-2-yl)amino)-1-oxobutan-2-yl)carbamate (compound* ***14****)*

The compound **14** was synthesized from compound **S26** using a similar preparation method as that for compound **13**, a white solid (63.1% yield). ^1^H-NMR (400 MHz, DMSO-*d6*) δ 10.02 (m, 2H), 8.63 (m, 1H), 8.28-8.11 (m, 2H), 7.96-7.82 (m, 2H), 7.59 (m, 5H), 7.34-7.21 (m, 9H), 7.04 (t, 1H), 5.98 (t, 1H), 5.43 (s, 2H), 5.06 (m, 2H), 4.52 (m, 1H), 4.40-4.28 (m, 4H), 4.18 (q, 2H), 3.99 (m, 1H), 3.32 (m, 9H), 2.87 (s, 2H), 2.17 (m, 3H), 1.99 (m, 2H), 1.87 (m, 2H), 1.71 (m, 1H), 1.59 (m, 1H), 1.45 (m, 6H), 1.19 (m, 2H), 0.91-0.77 (m, 18H). MS (ESI) m/z: 1156.41 [M+H]^+^; 1178.56 [M+Na]^+^.

**Original Mass and NMR spectrogram**


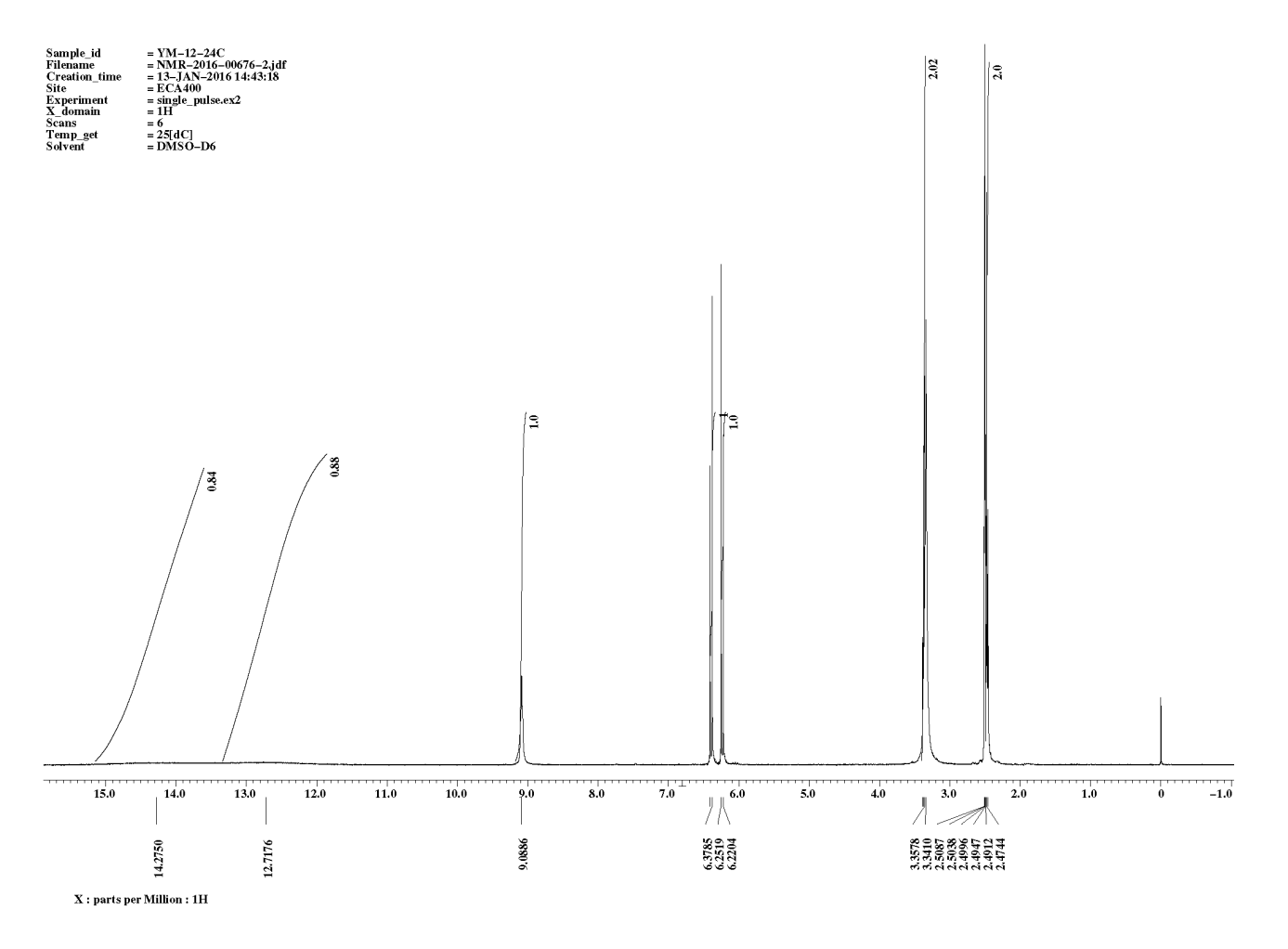


The ^1^H-NMR spectrum of Compd. **3a**


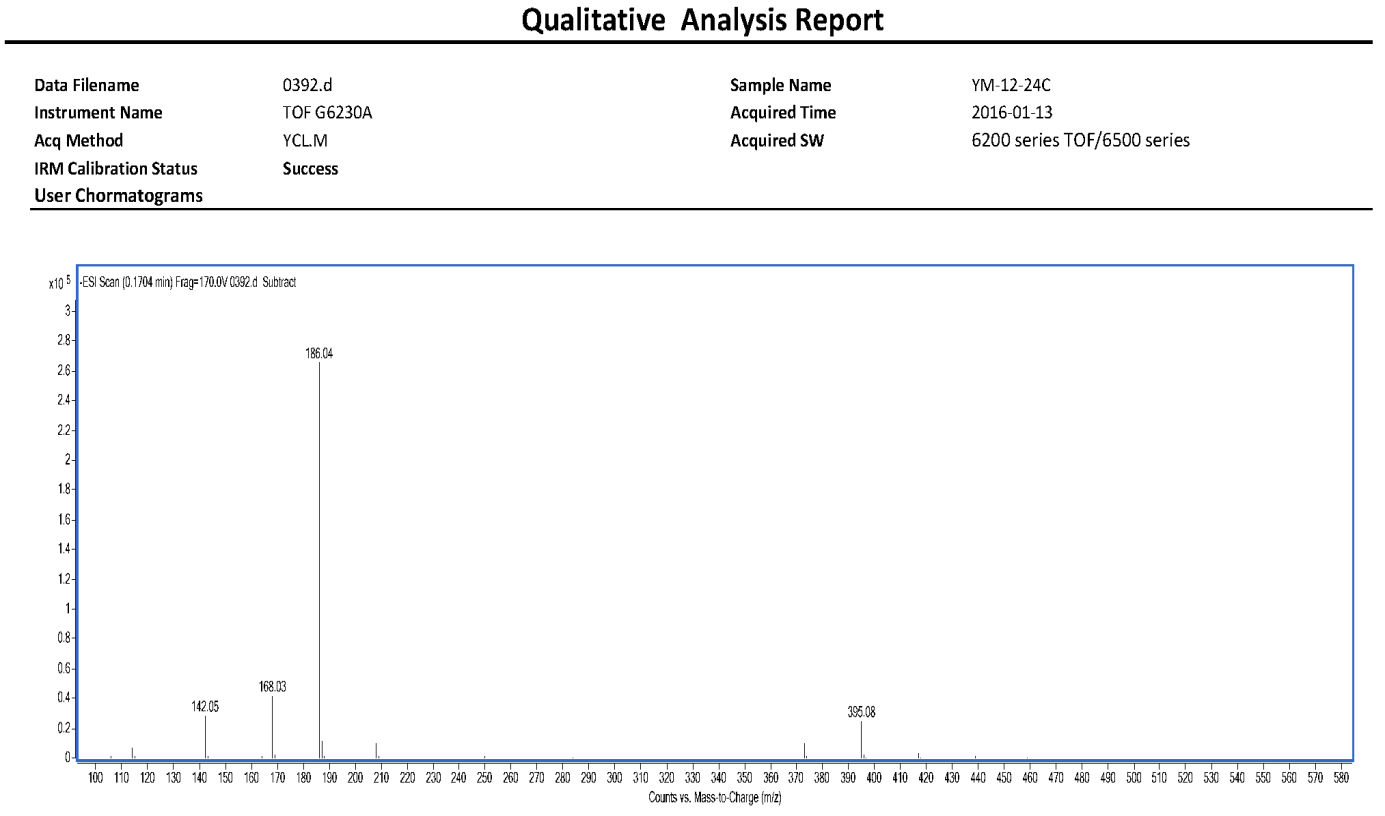


The MS spectrum of Compd. **3a**





The ^1^H-NMR spectrum of Compd. **3b**


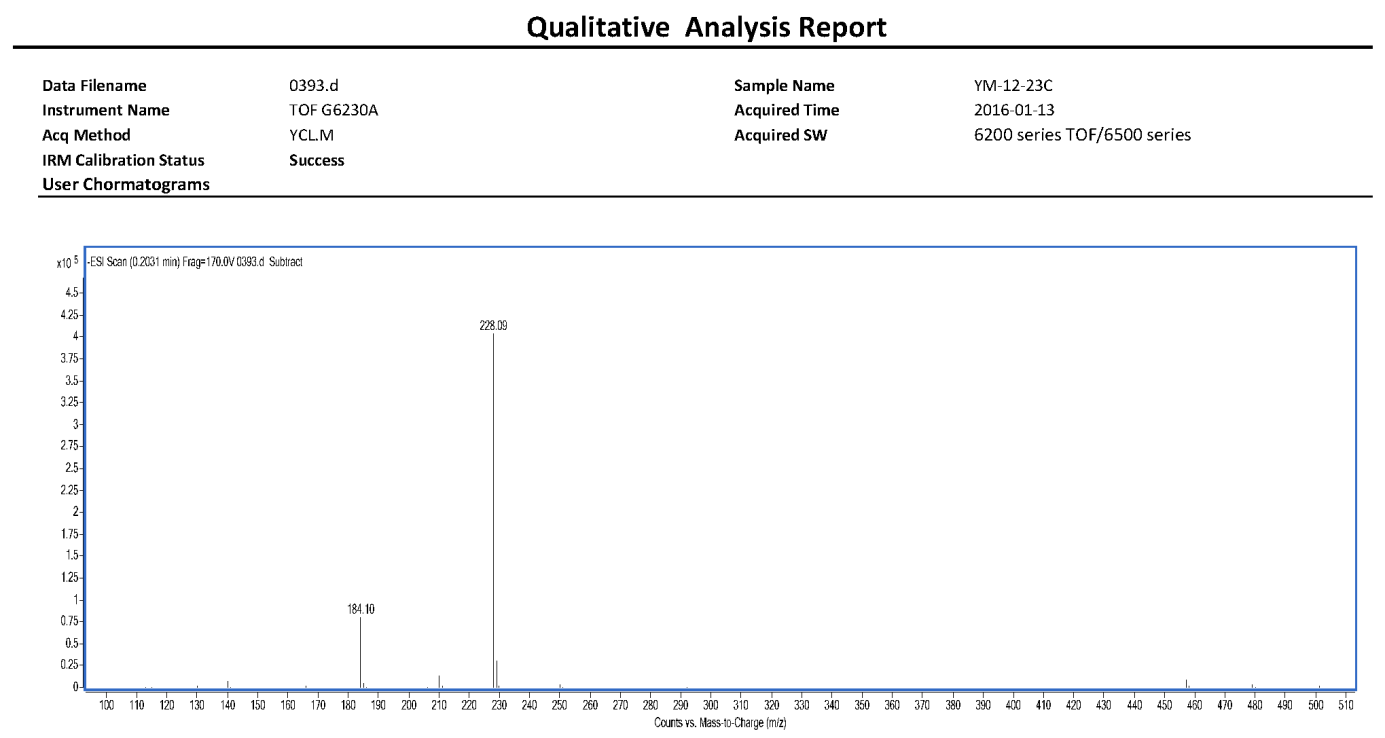


The MS spectrum of Compd. **3b**


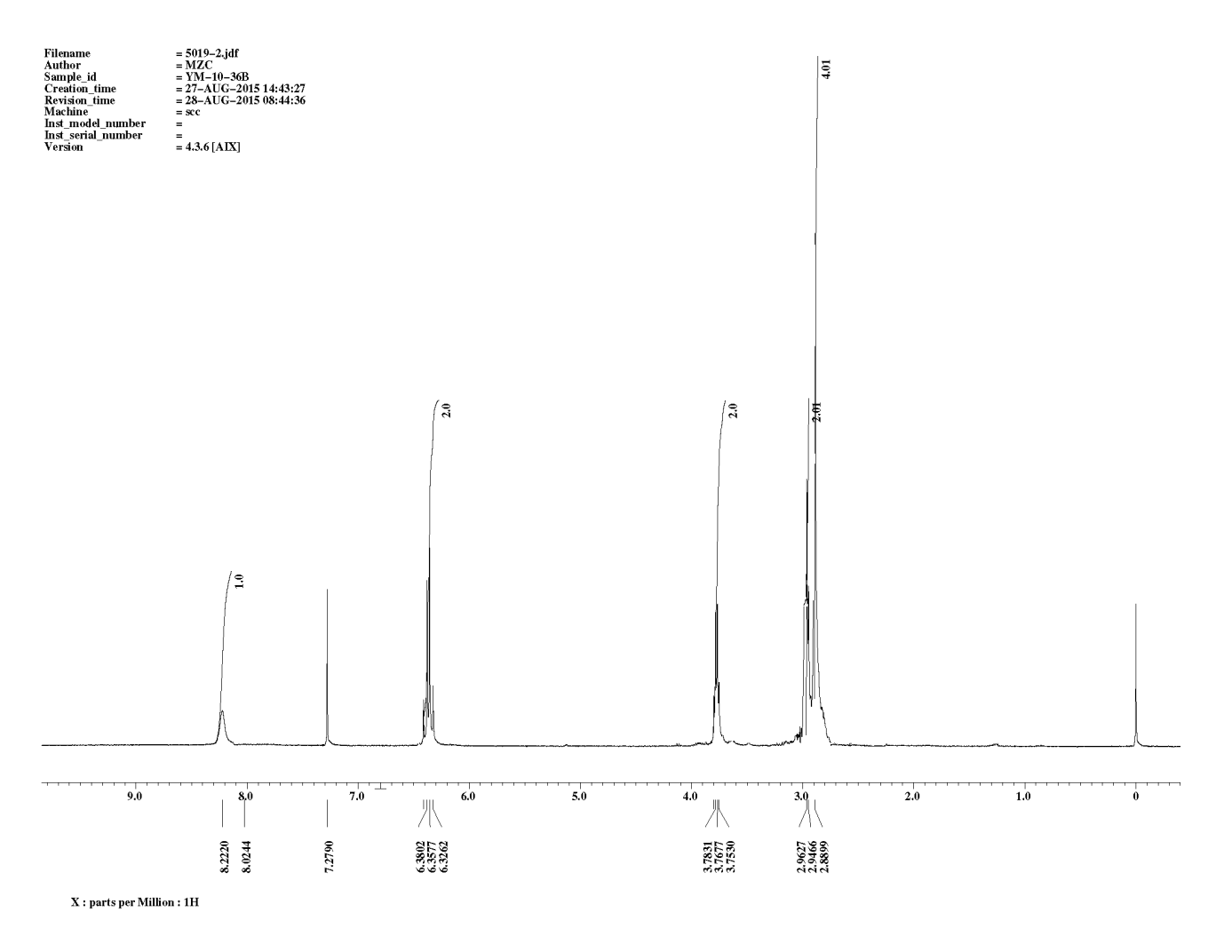


The ^1^H-NMR spectrum of Compd. **4a**


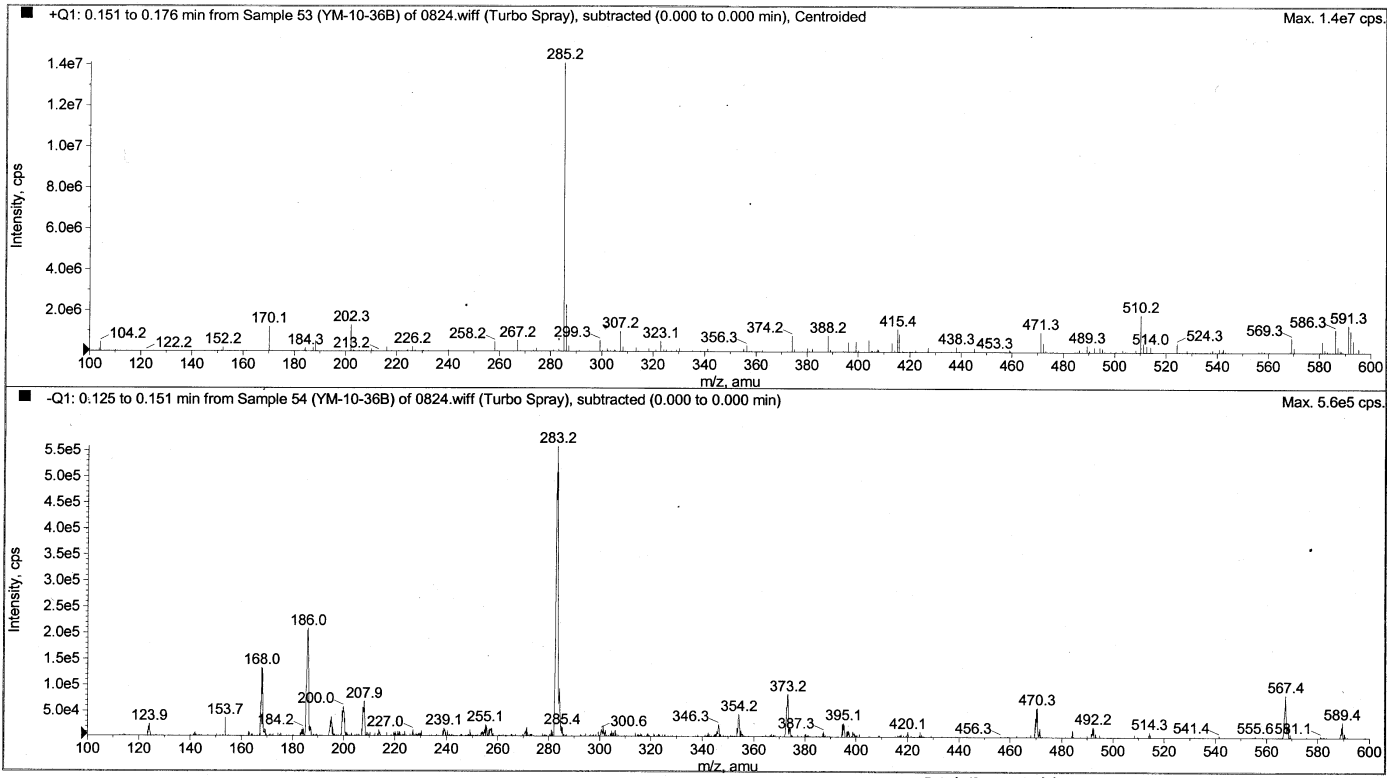


The MS spectrum of Compd. **4a**


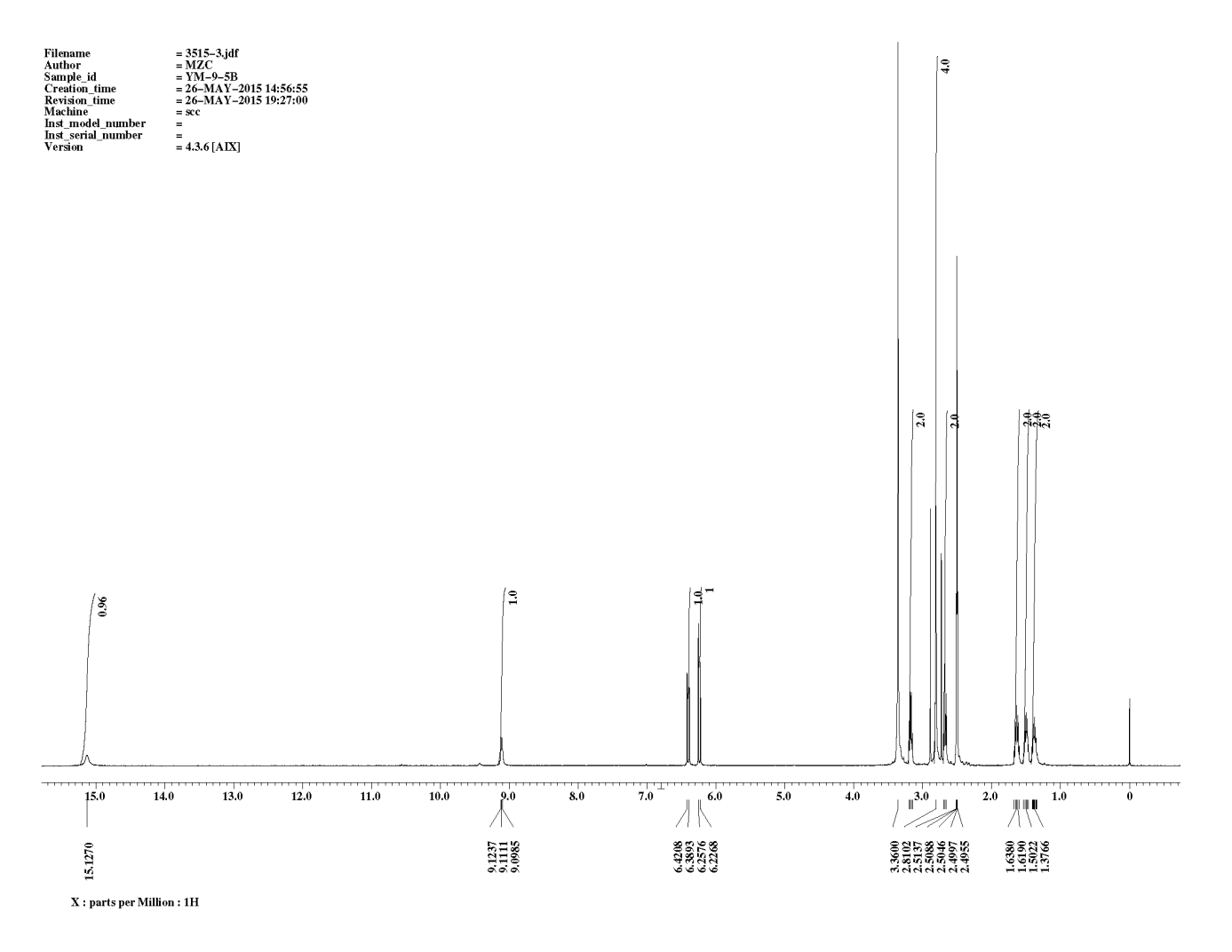


The ^1^H-NMR spectrum of Compd. **4b**


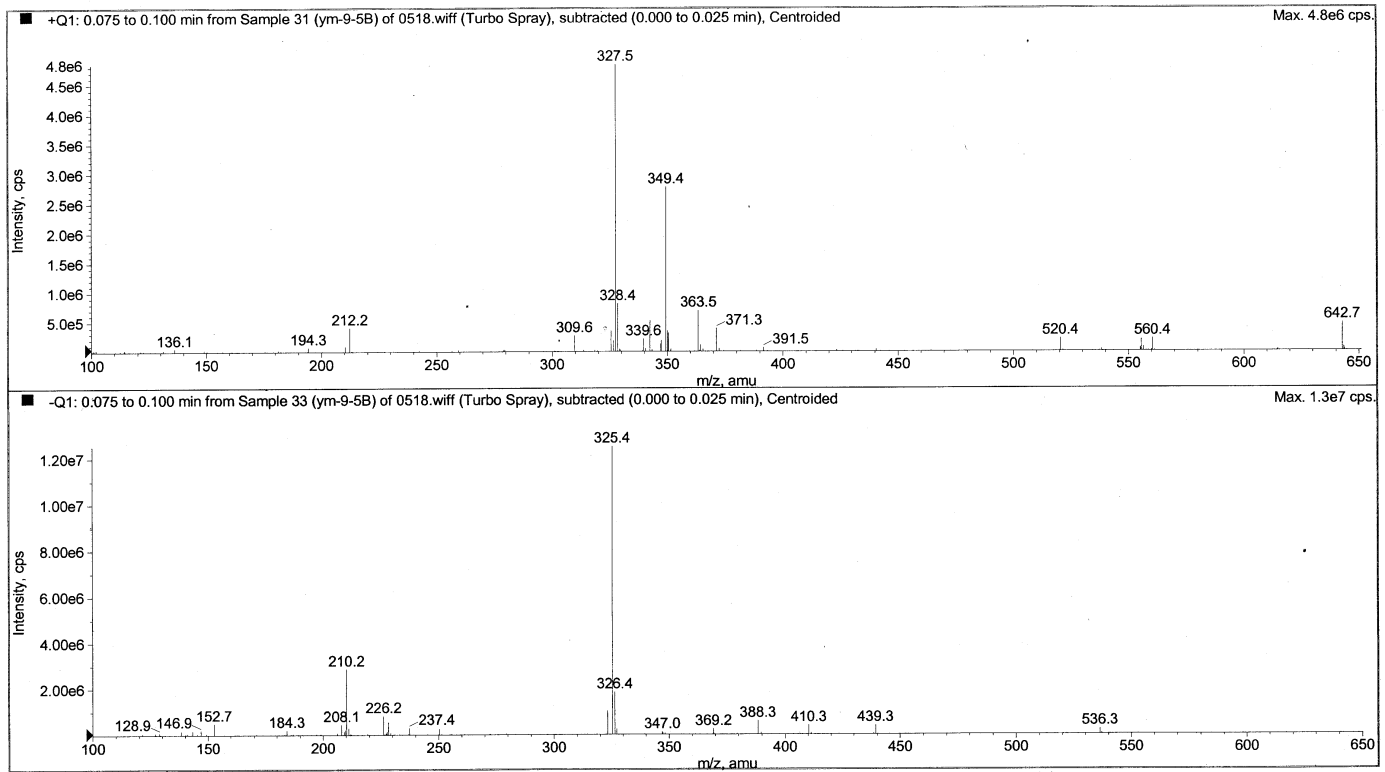


The MS spectrum of Compd. **4b**


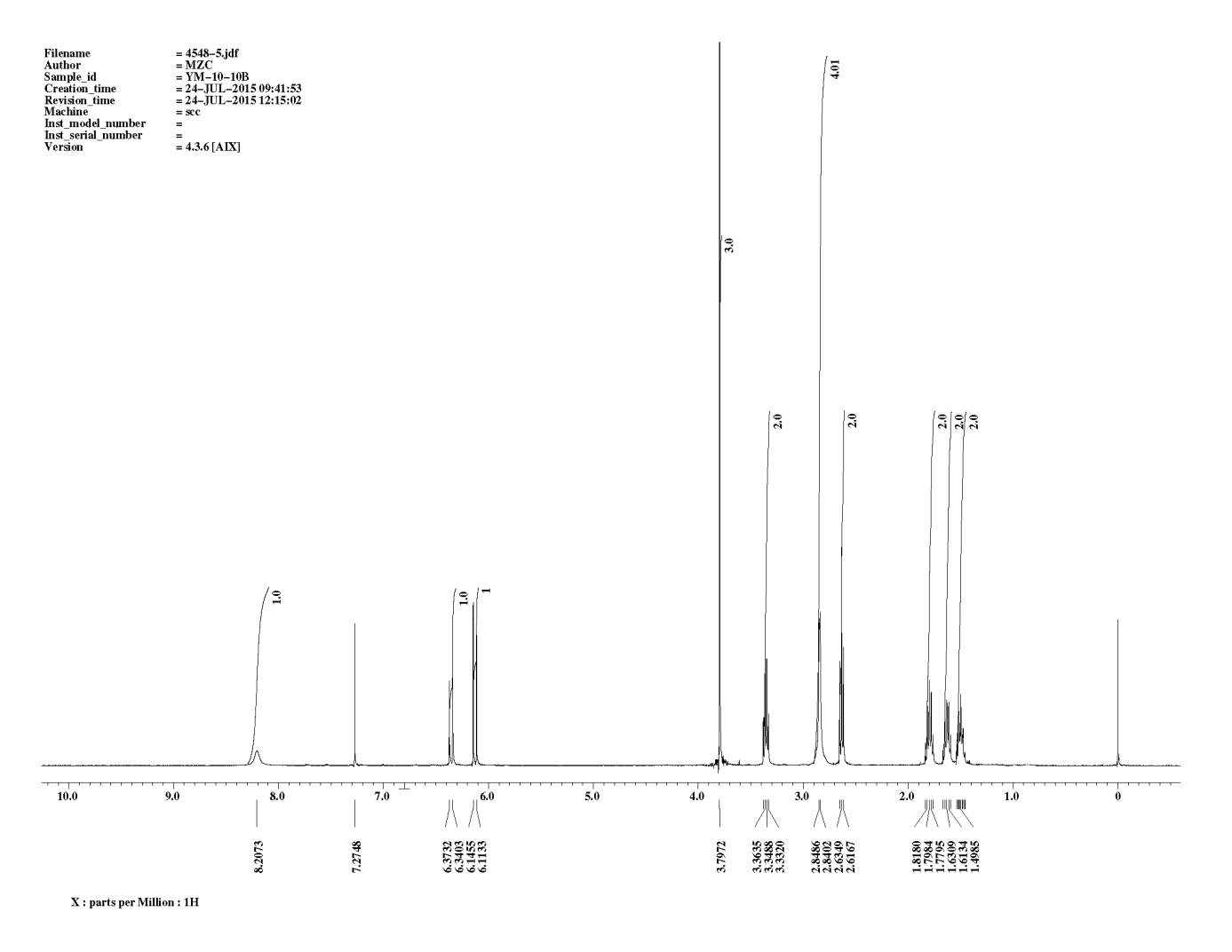


The ^1^H-NMR spectrum of Compd. **5b**


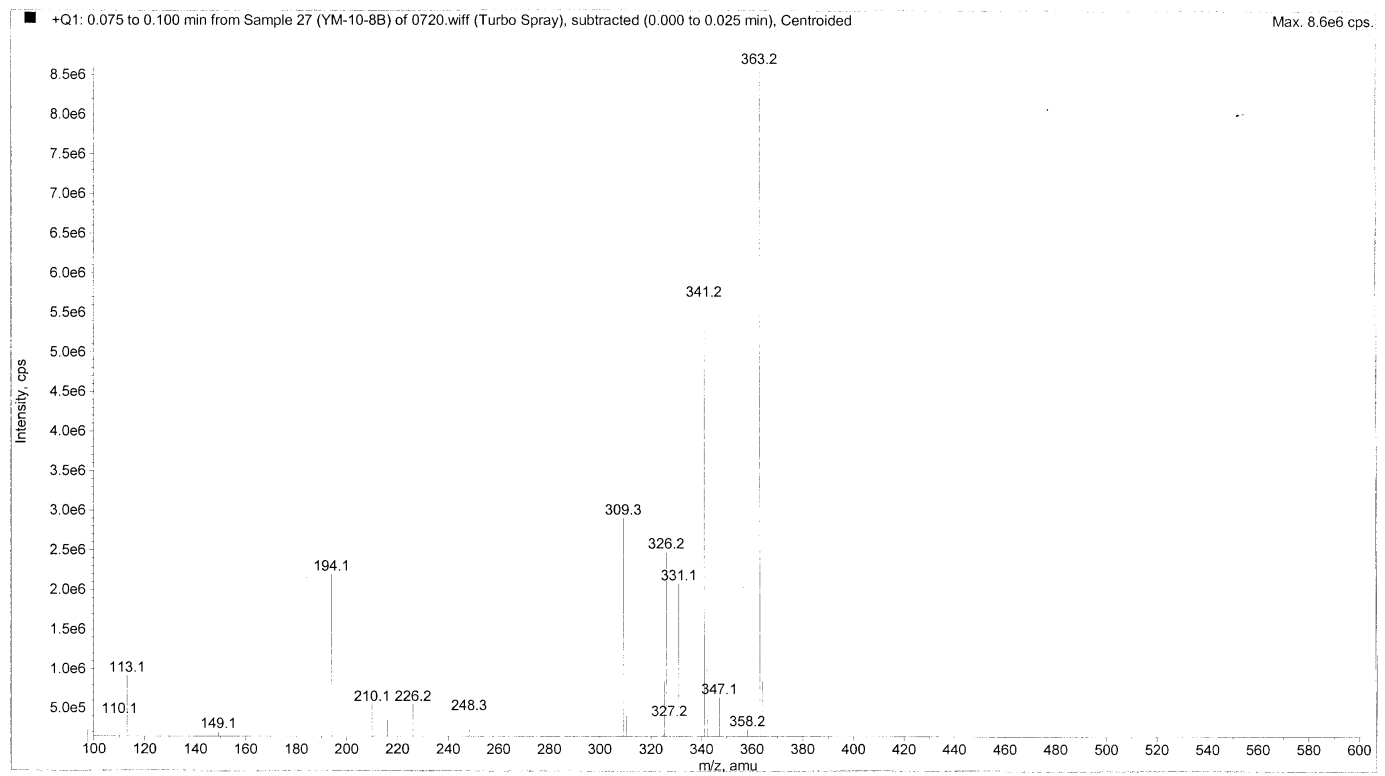


The MS spectrum of Compd. **5b**





The ^1^H-NMR spectrum of Compd. **7a**


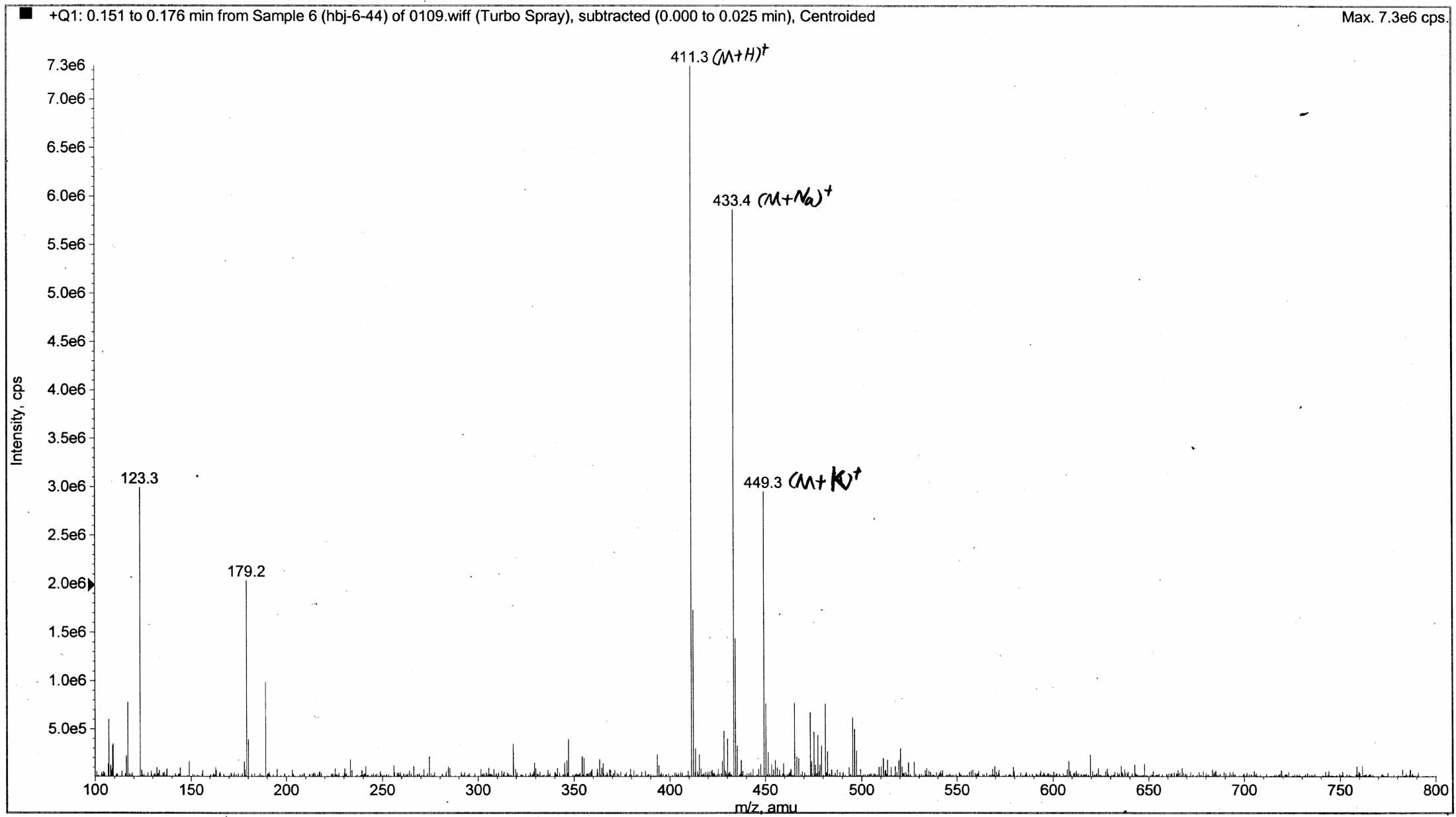


The MS spectrum of Compd. **7a**


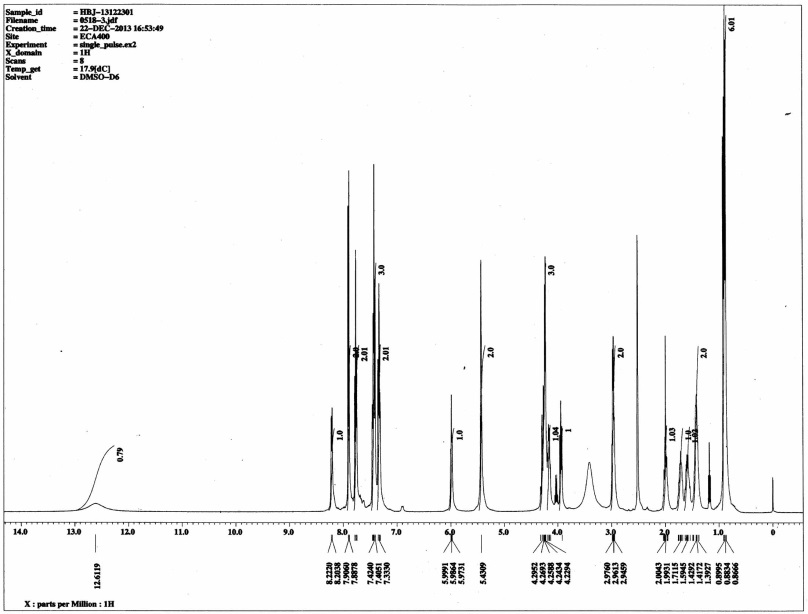


The ^1^H-NMR spectrum of Compd. **7b**


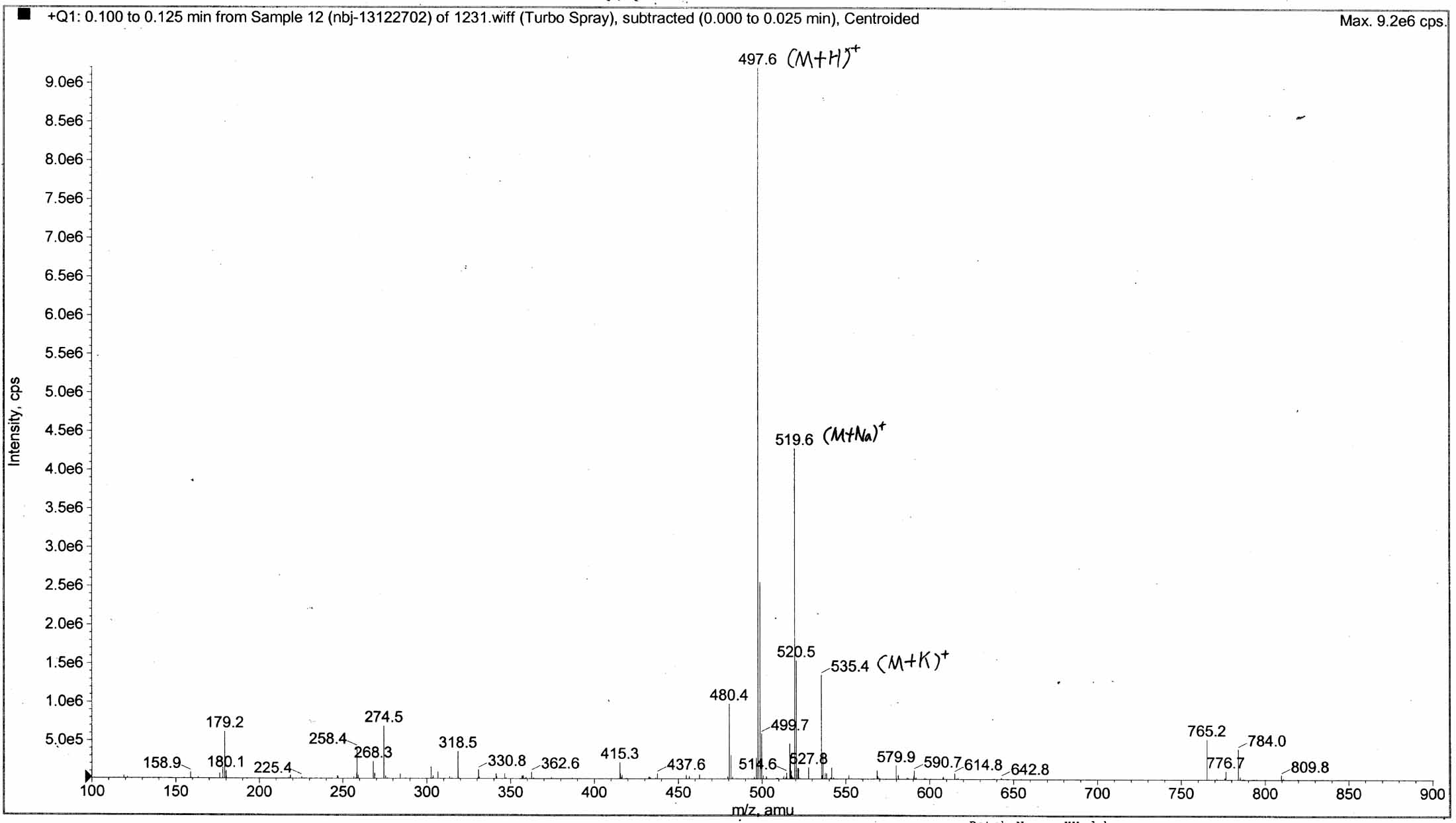


The MS spectrum of Compd. **7b**





The ^1^H-NMR spectrum of Compd. **8a**


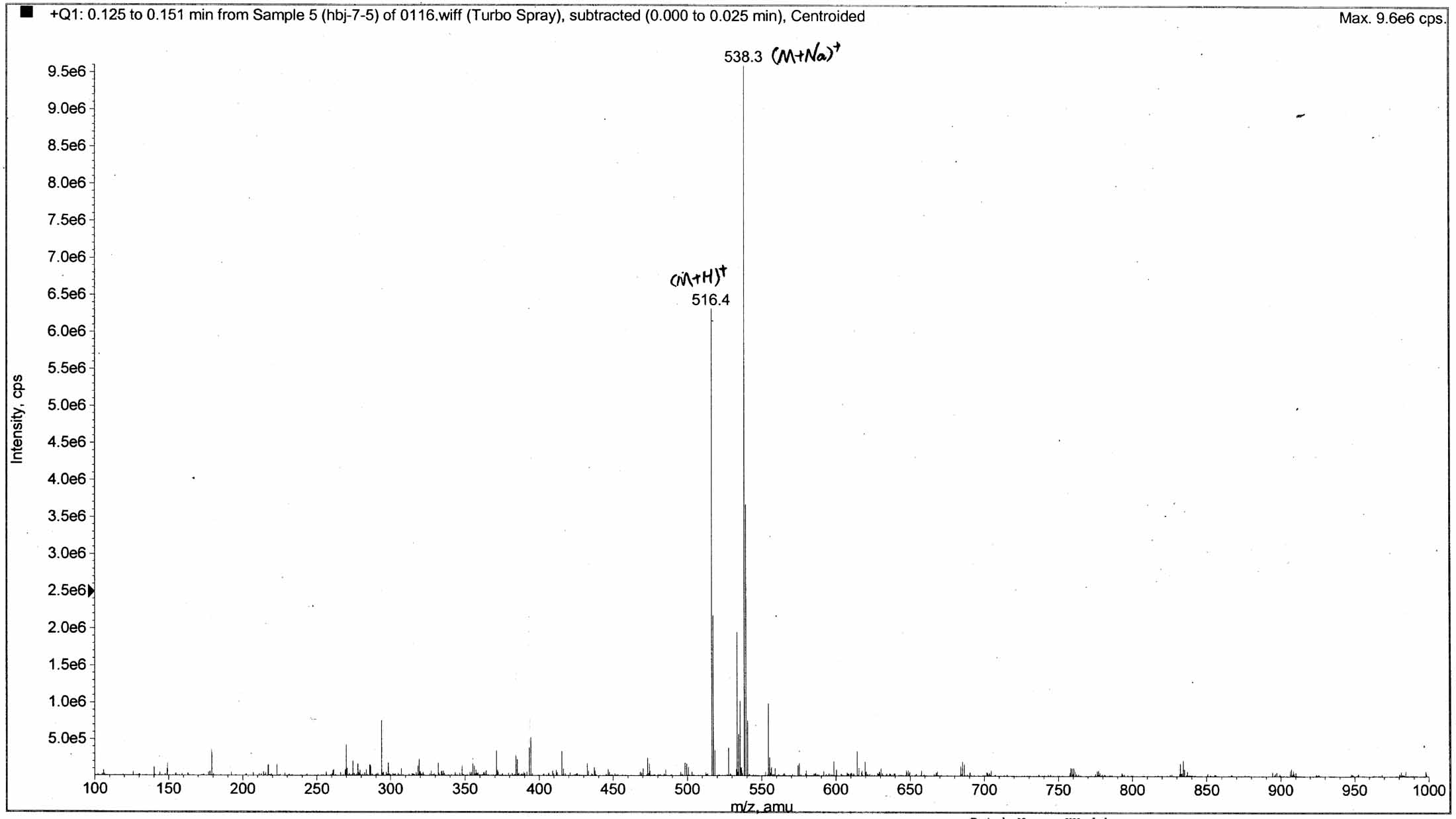


The MS spectrum of Compd. **8a**


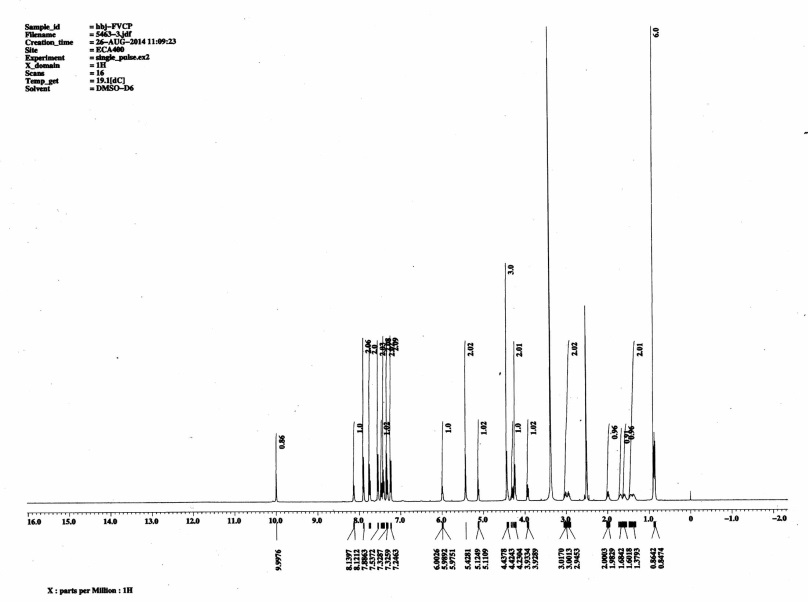


The ^1^H-NMR spectrum of Compd. **8b**


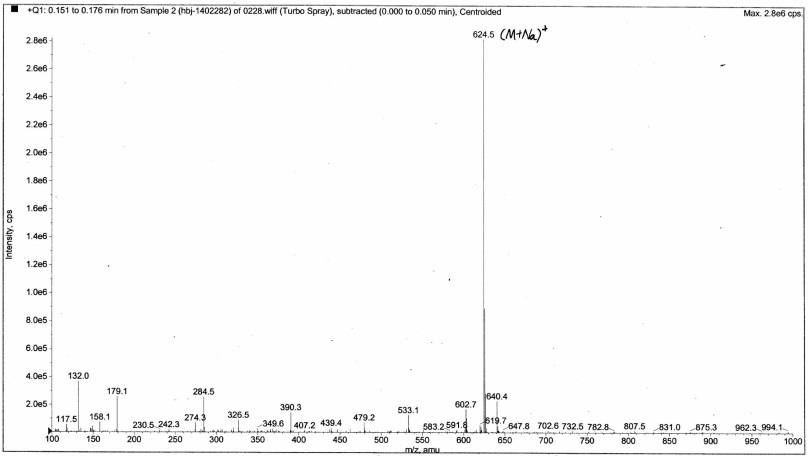


The MS spectrum of Compd. **8b**


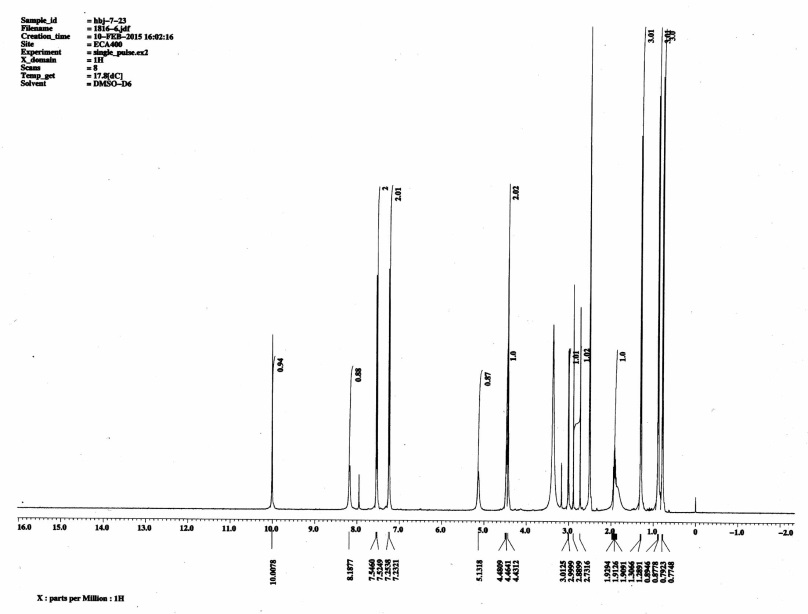


The ^1^H-NMR spectrum of Compd. **9a**


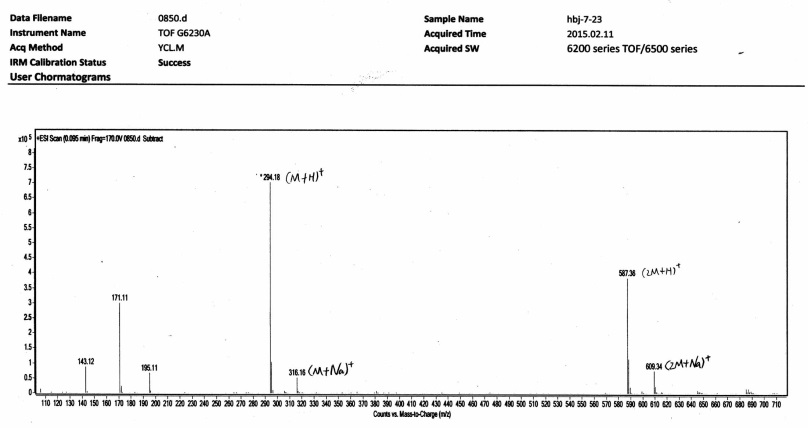


The MS spectrum of Compd. **9a**


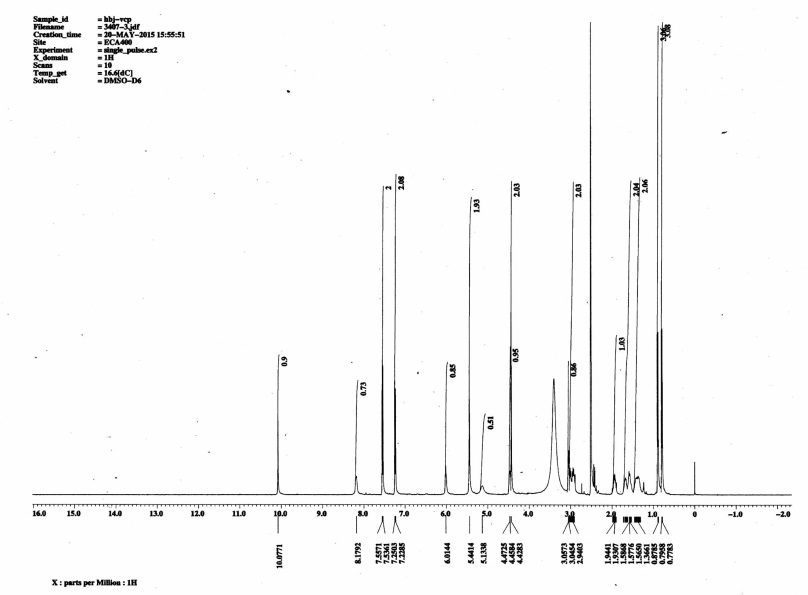


The ^1^H-NMR spectrum of Compd. **9b**


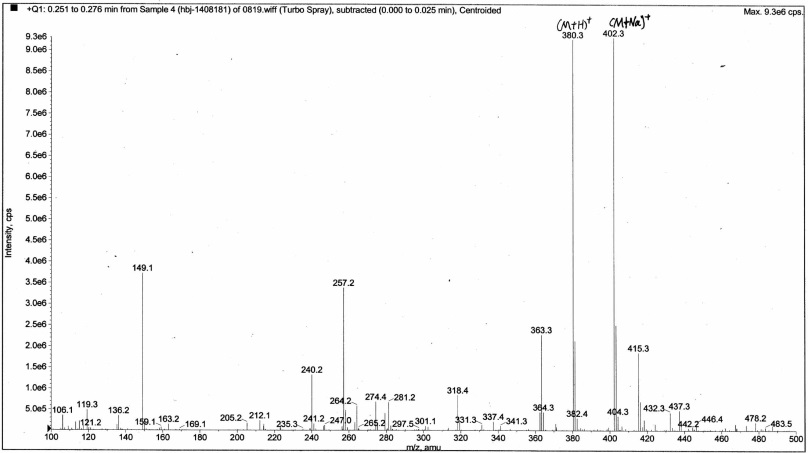


The MS spectrum of Compd. **9b**





The ^1^H-NMR spectrum of Compd. **10a**


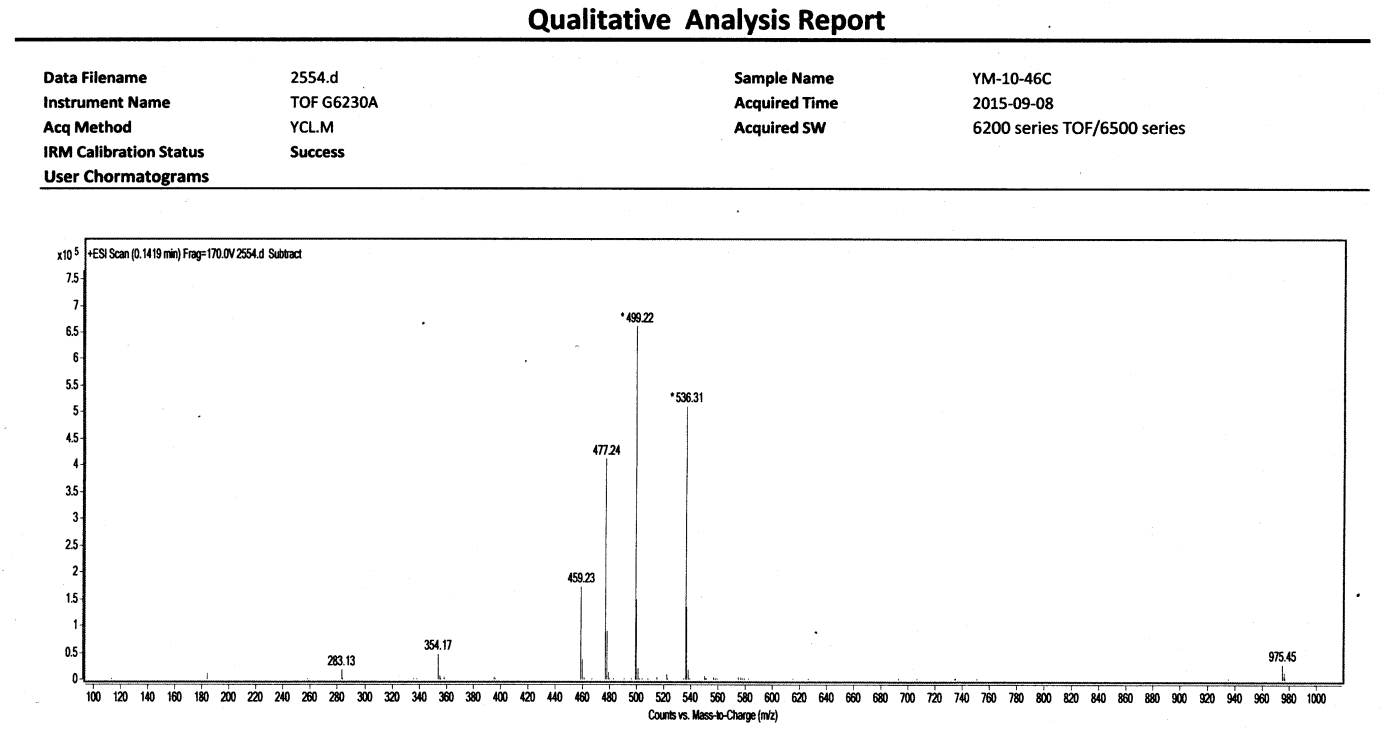


The MS spectrum of Compd. **10a**


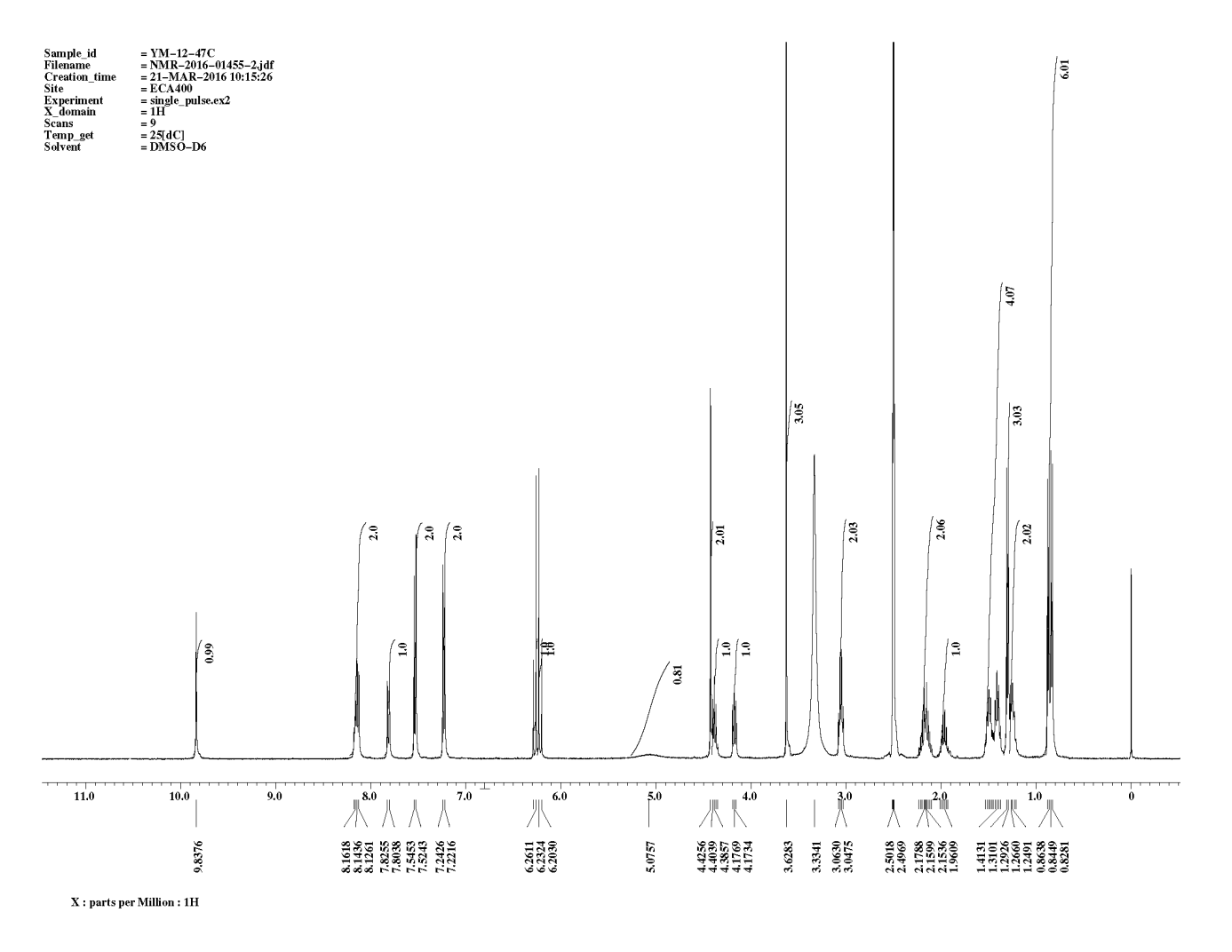


The ^1^H-NMR spectrum of Compd. **10b**


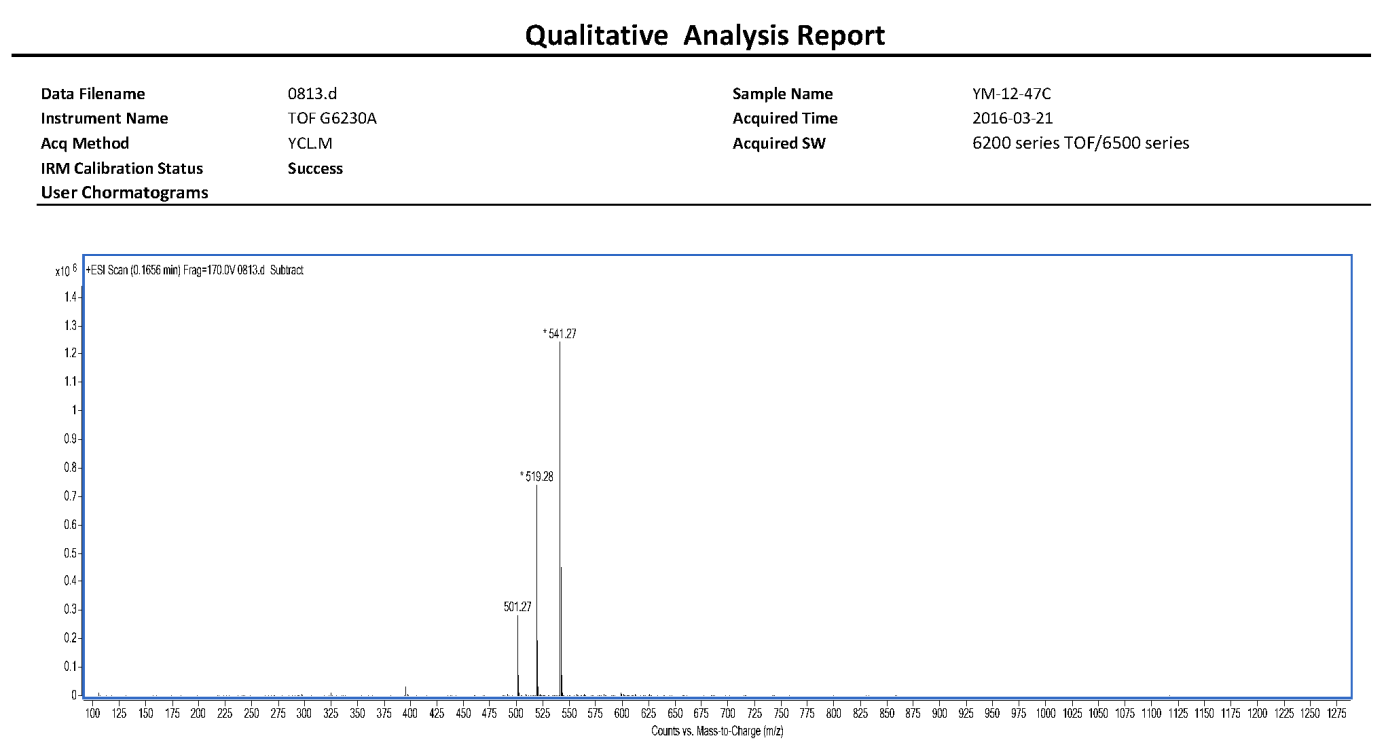


The MS spectrum of Compd. **10b**


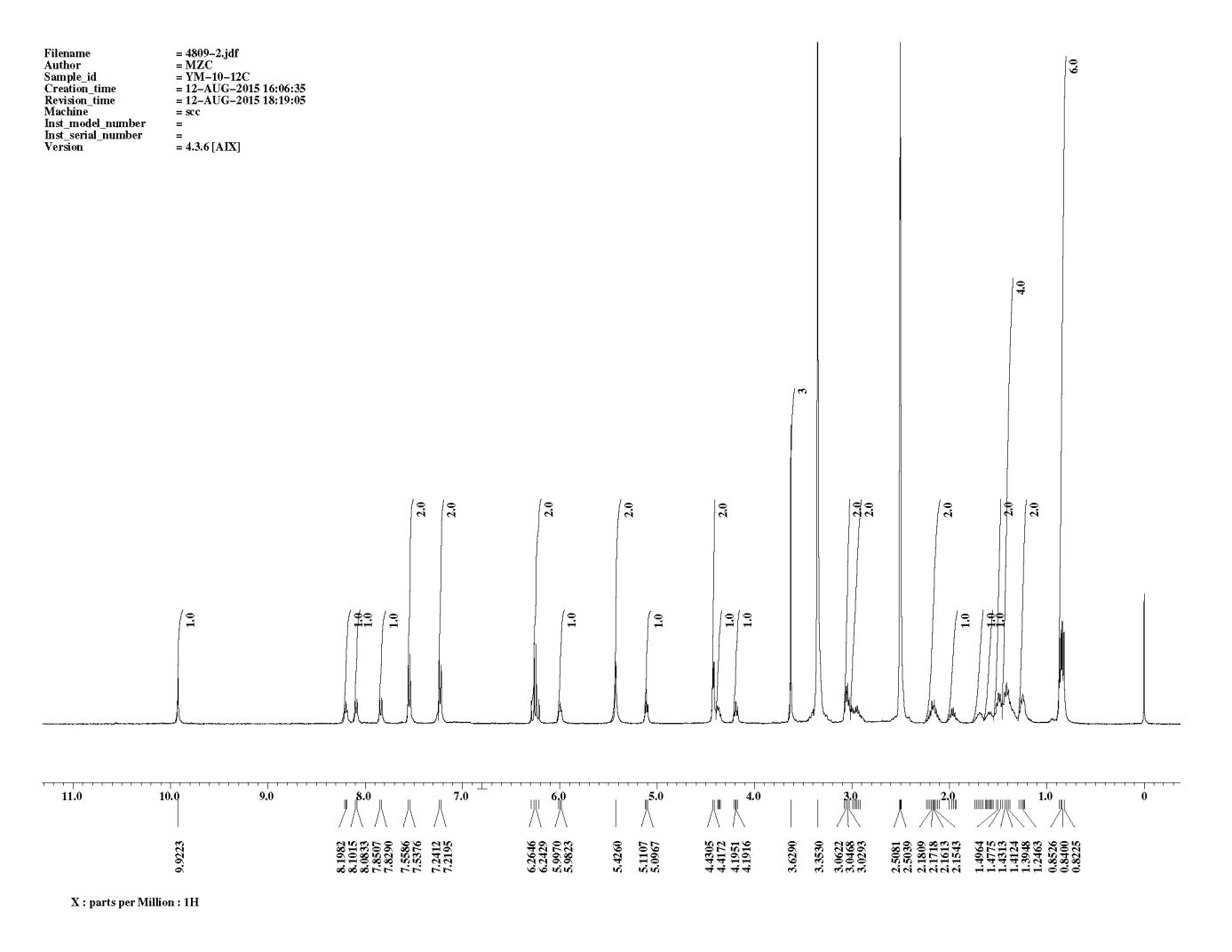


The ^1^H-NMR spectrum of Compd. **10c**


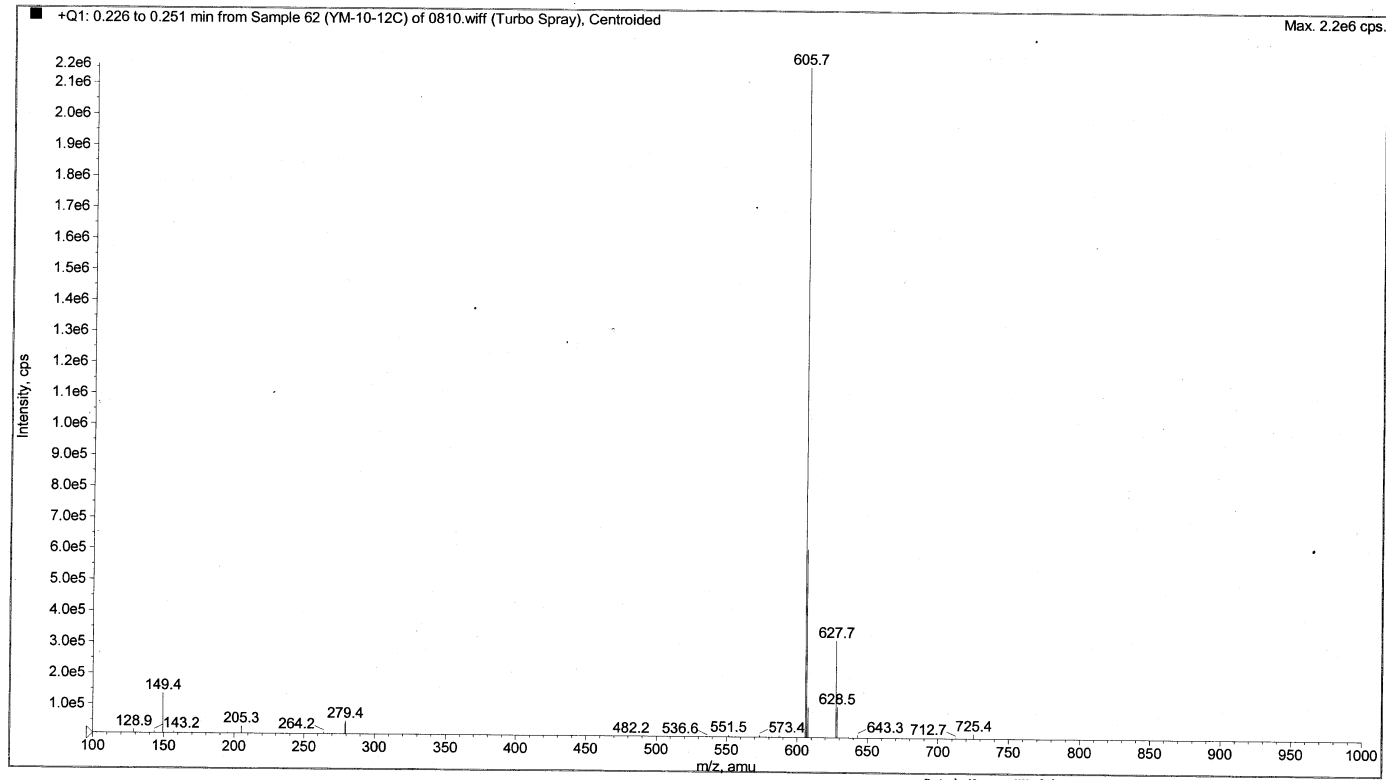


The MS spectrum of Compd. **10c**


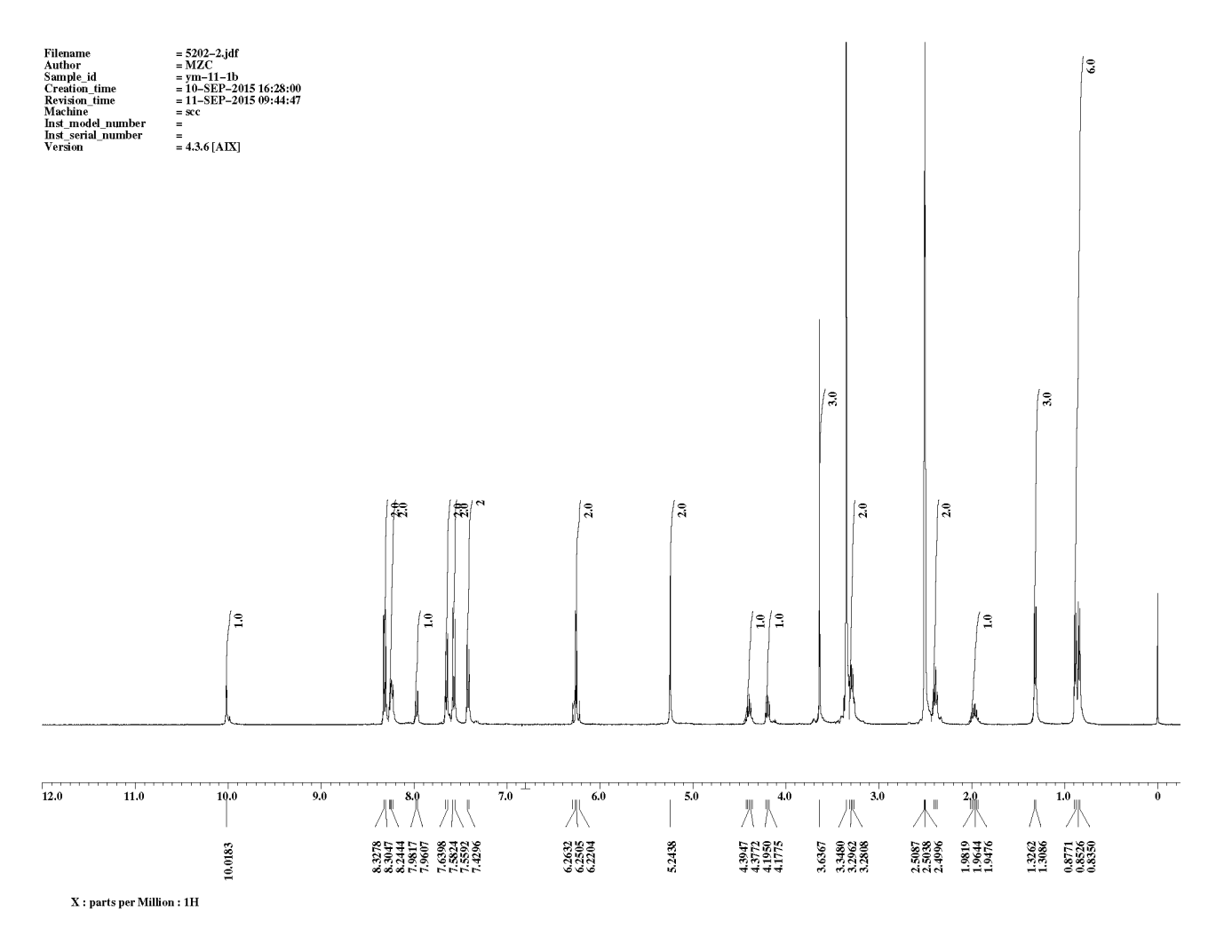


The ^1^H-NMR spectrum of Compd. **11a**


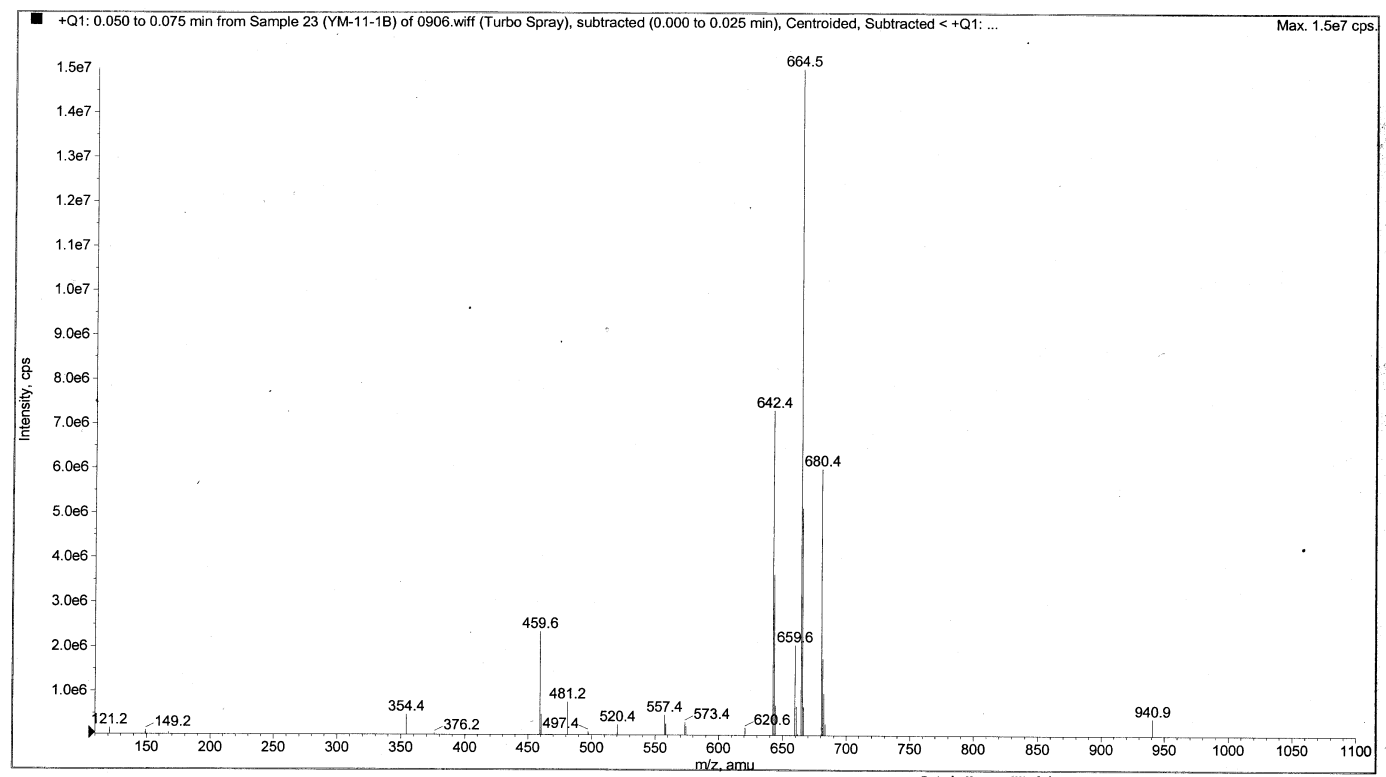


The MS spectrum of Compd. **11a**


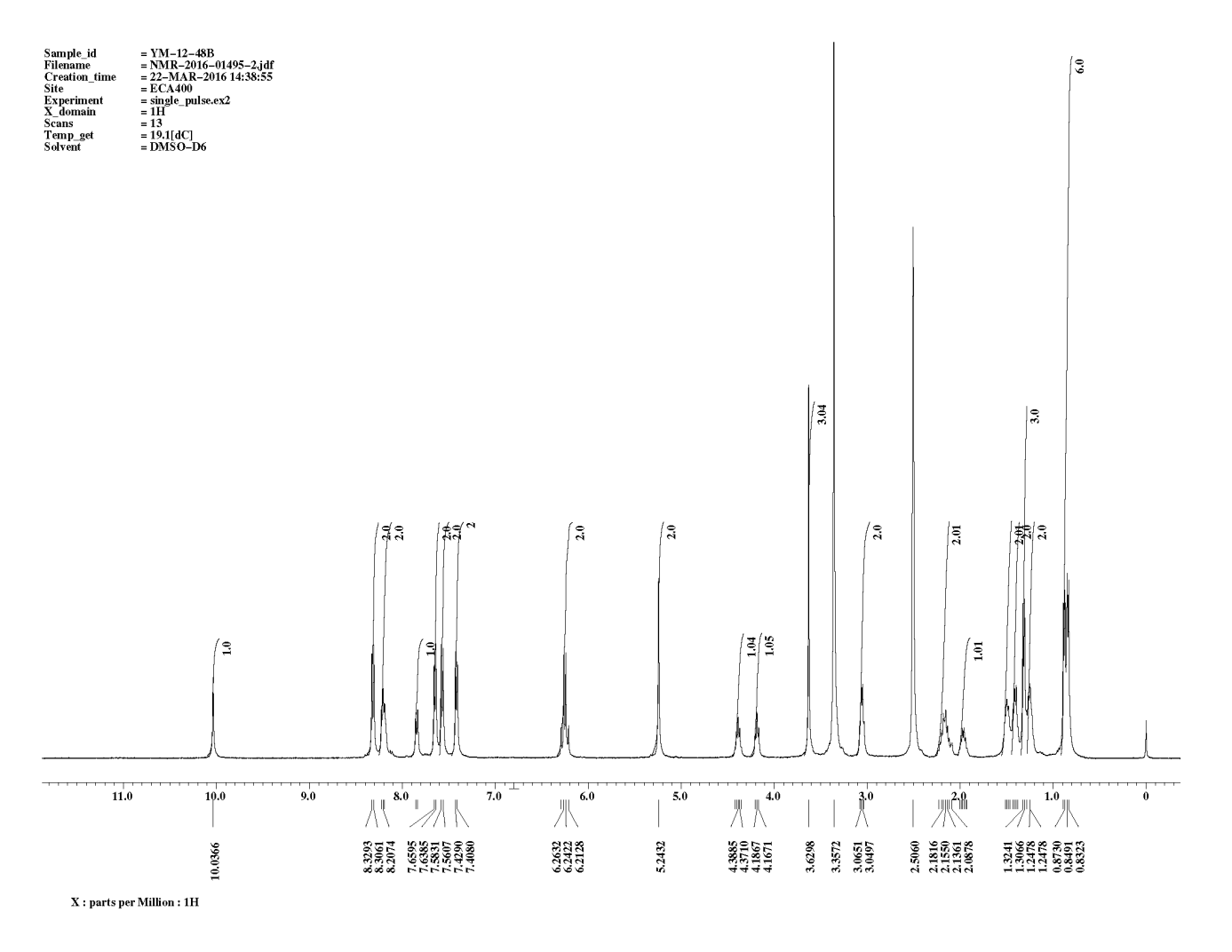


The ^1^H-NMR spectrum of Compd. **11b**


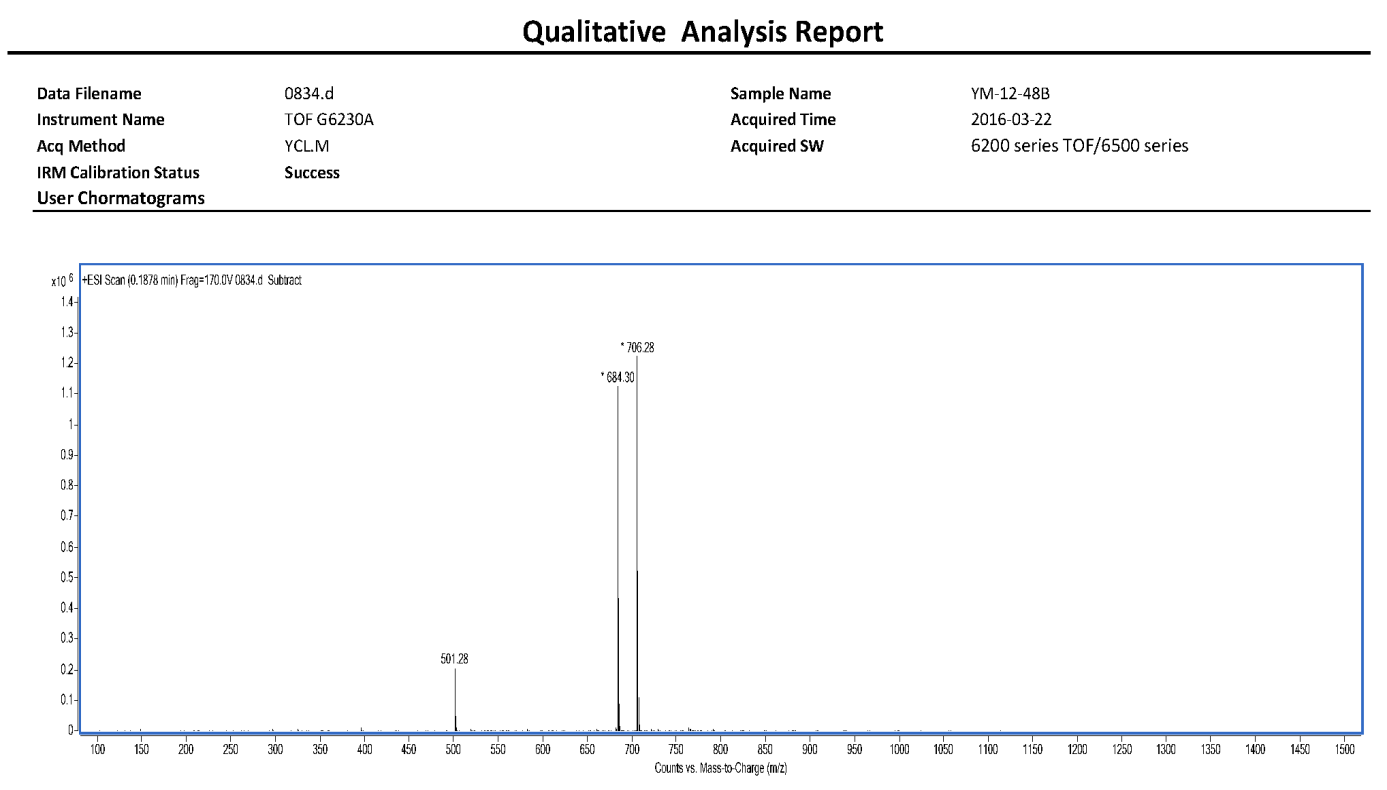


The MS spectrum of Compd. **11b**


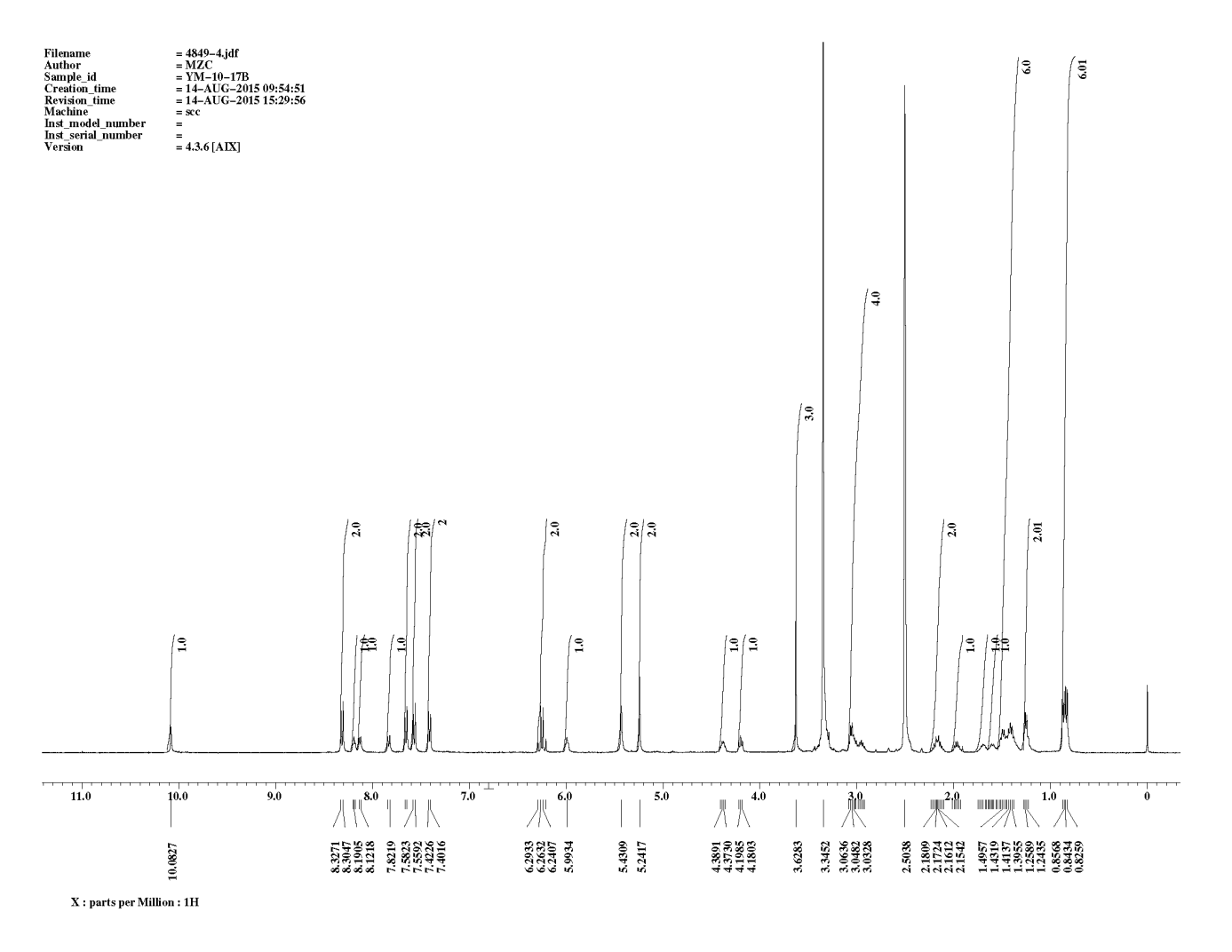


The ^1^H-NMR spectrum of Compd. **11c**


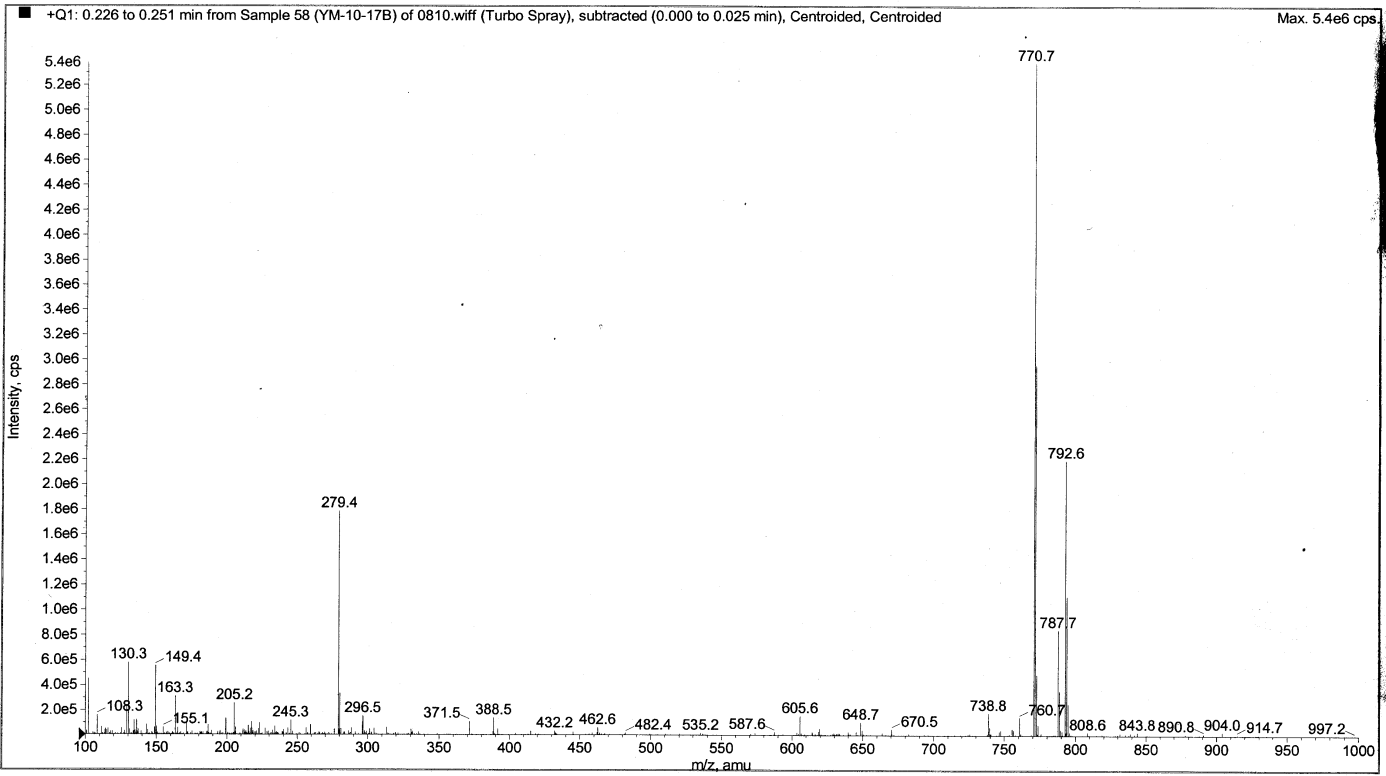


The MS spectrum of Compd. **11c**


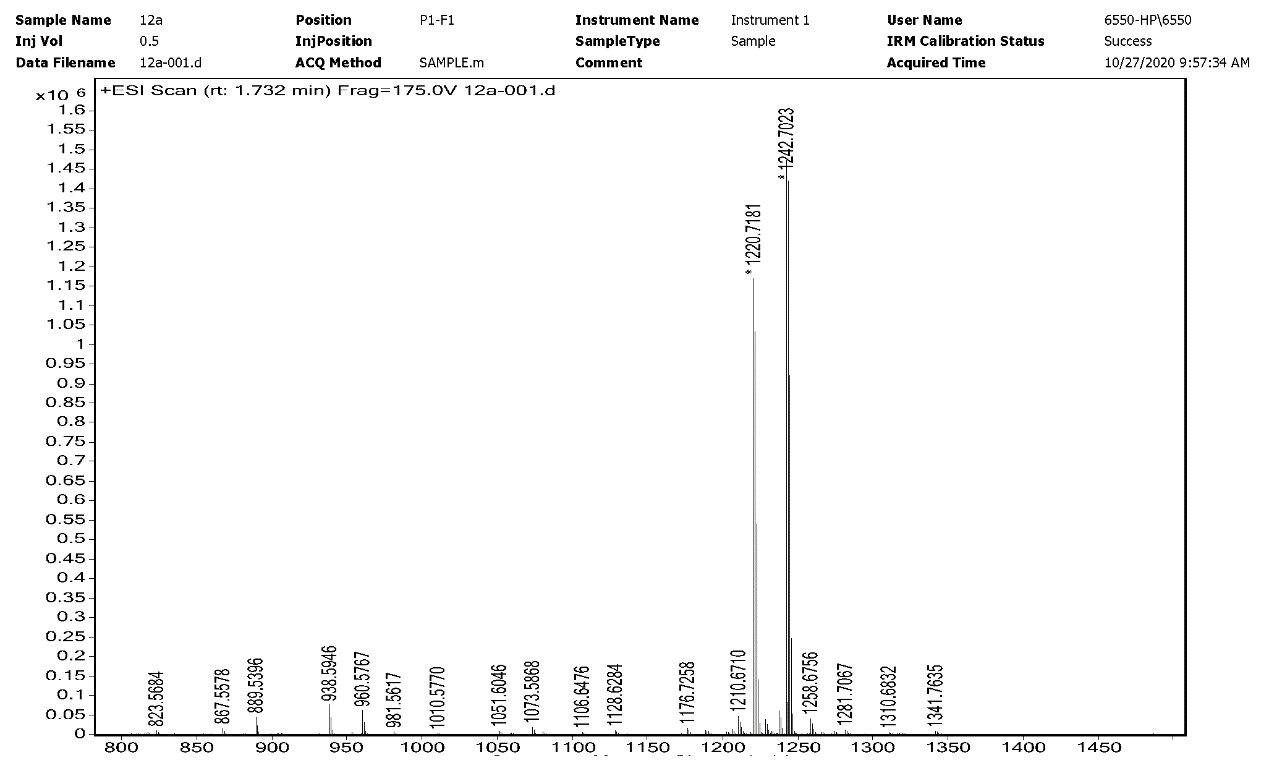


The HR-MS spectrum of Compd. **12a**


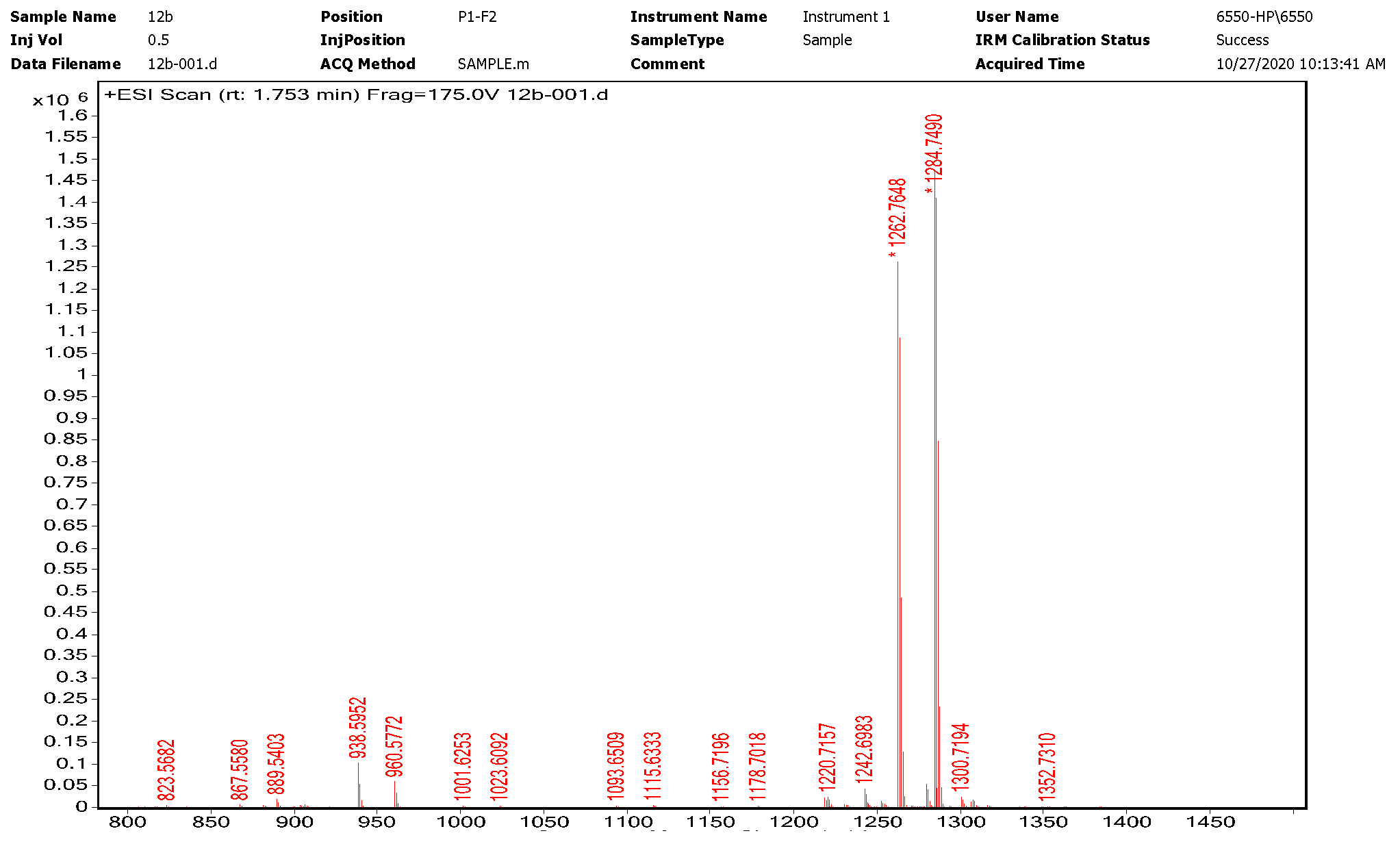


The HR-MS spectrum of Compd. **12b**


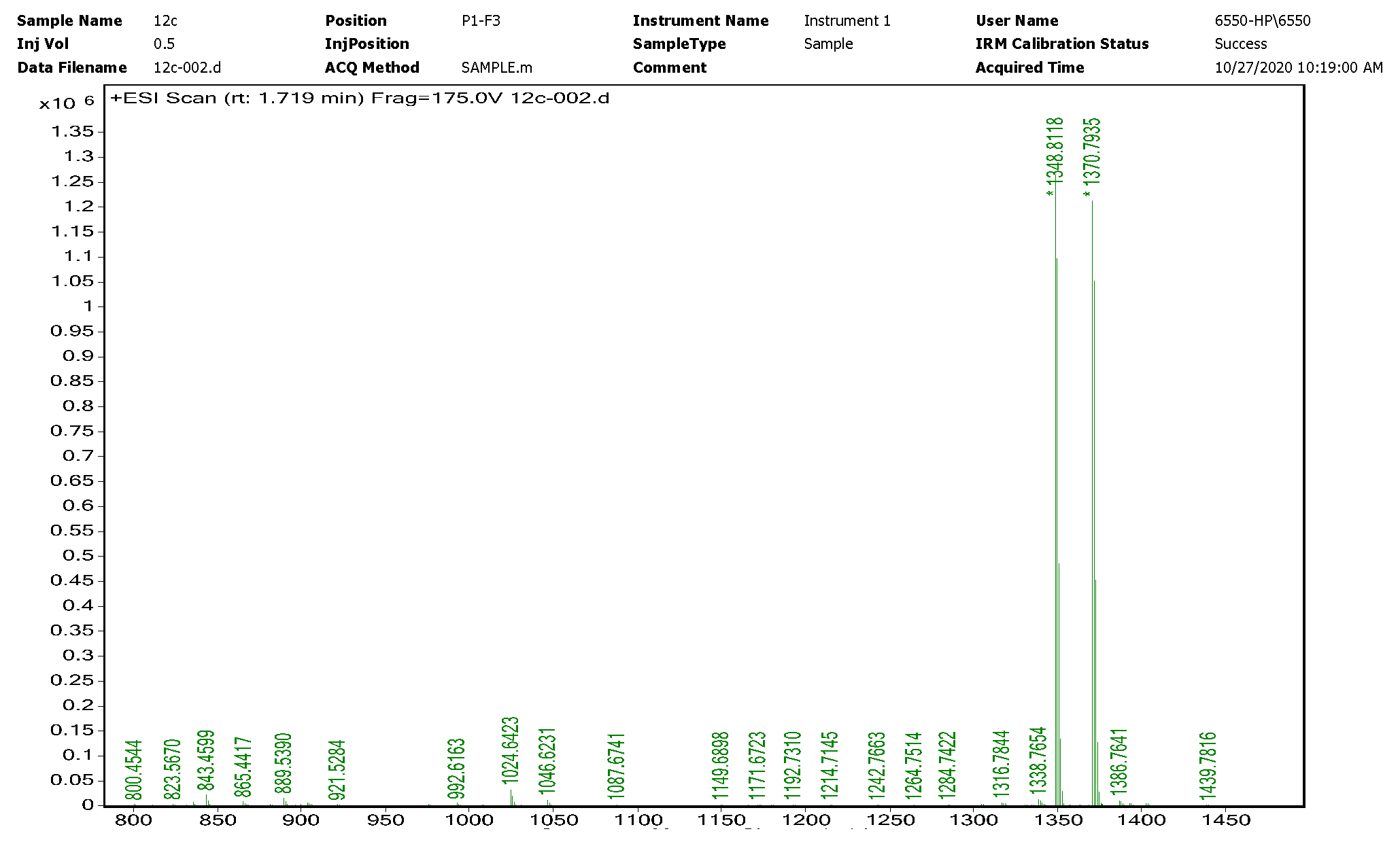


The HR-MS spectrum of Compd. **12c**


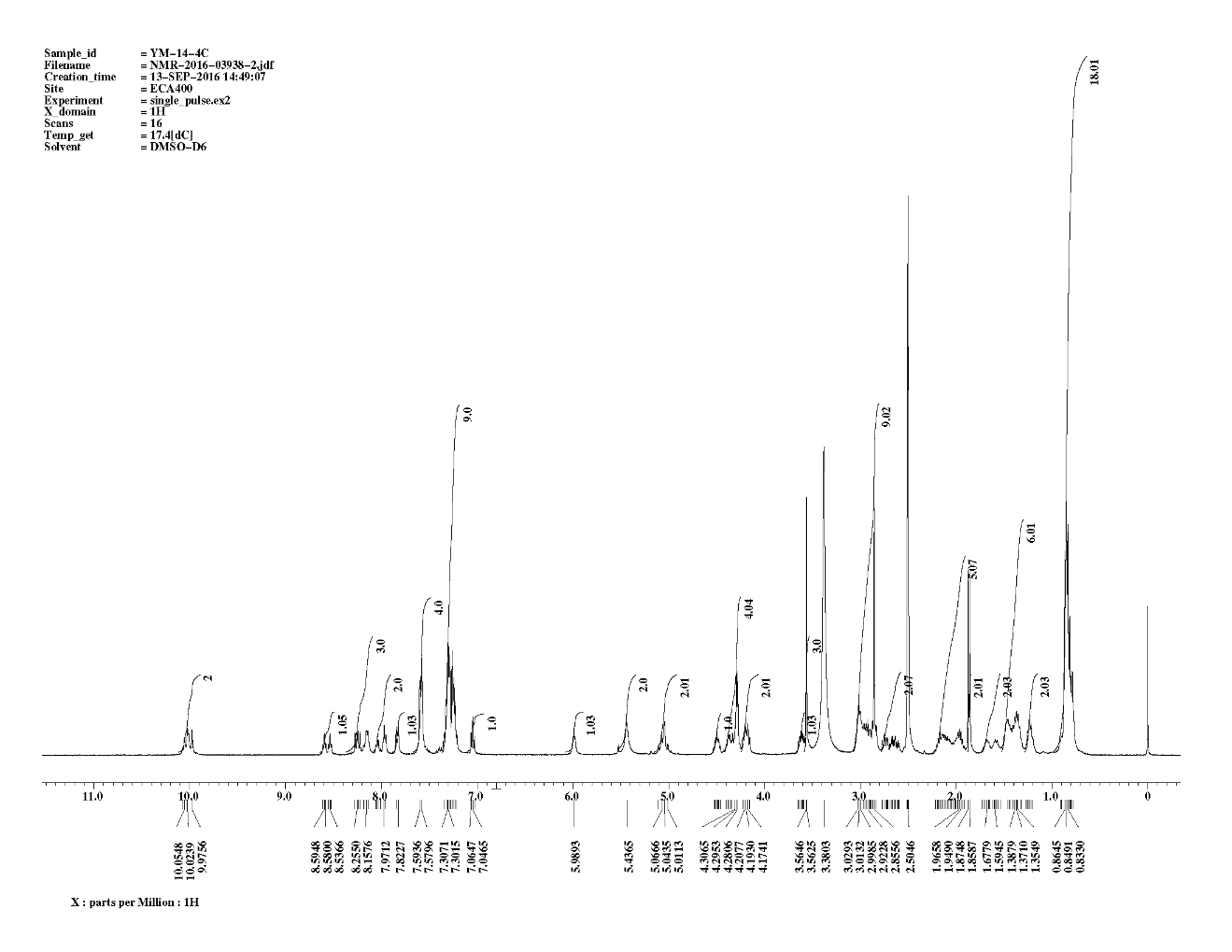


The ^1^H-NMR spectrum of Compd. **13**


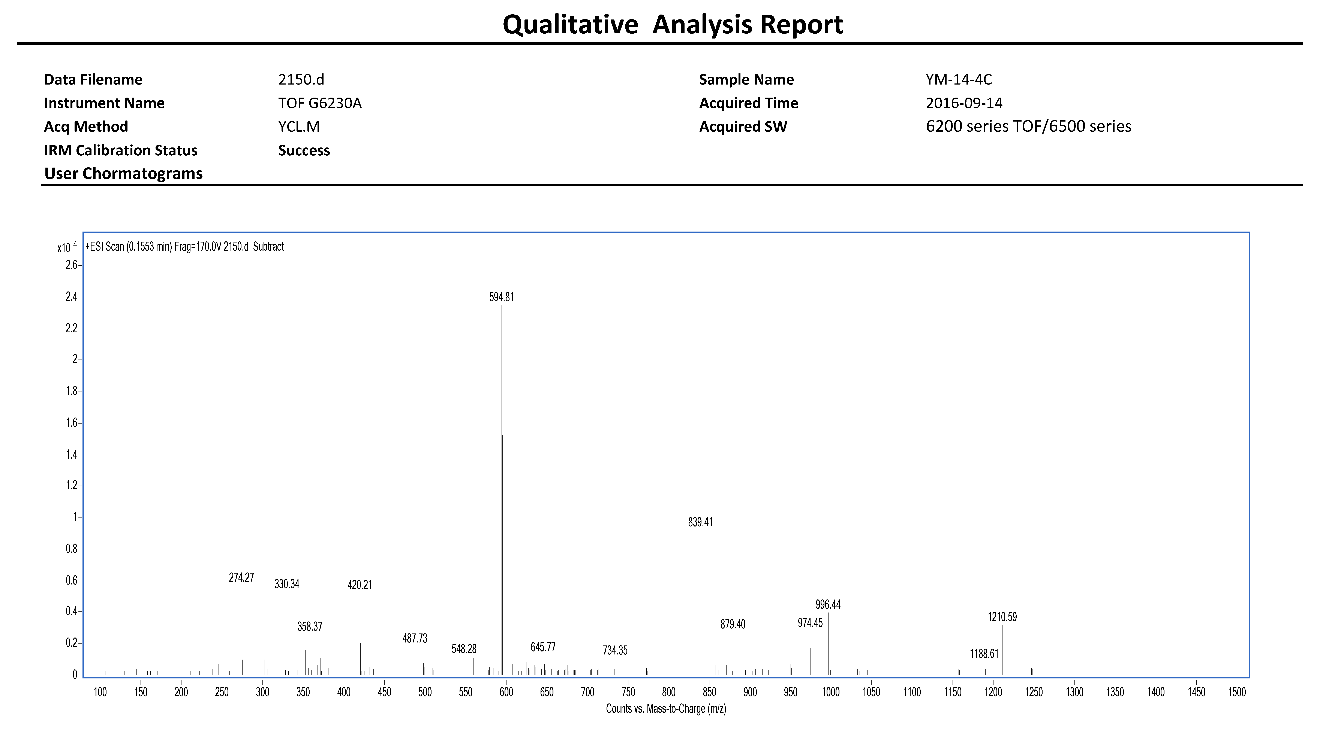


The MS spectrum of Compd. **13**


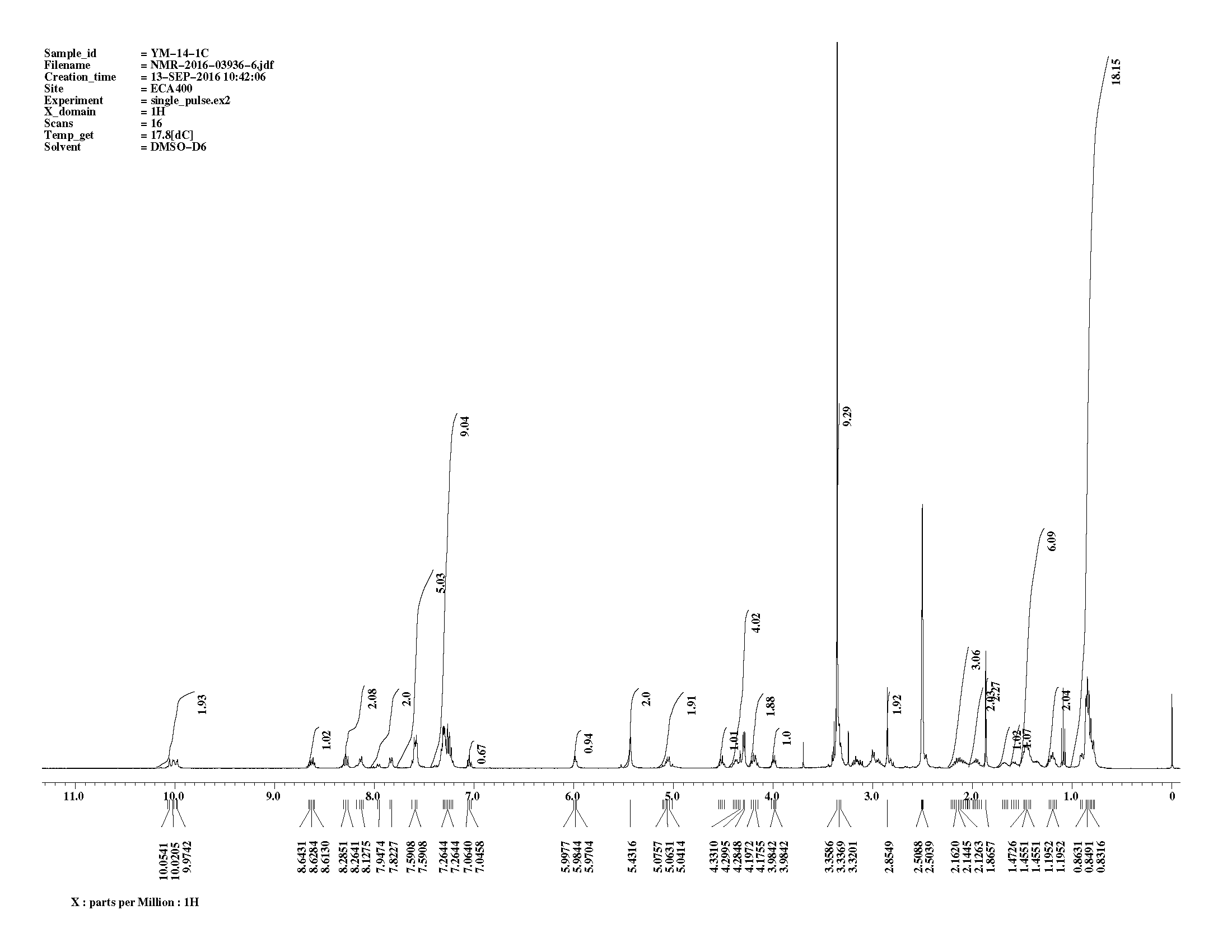


The ^1^H-NMR spectrum of Compd. **14**


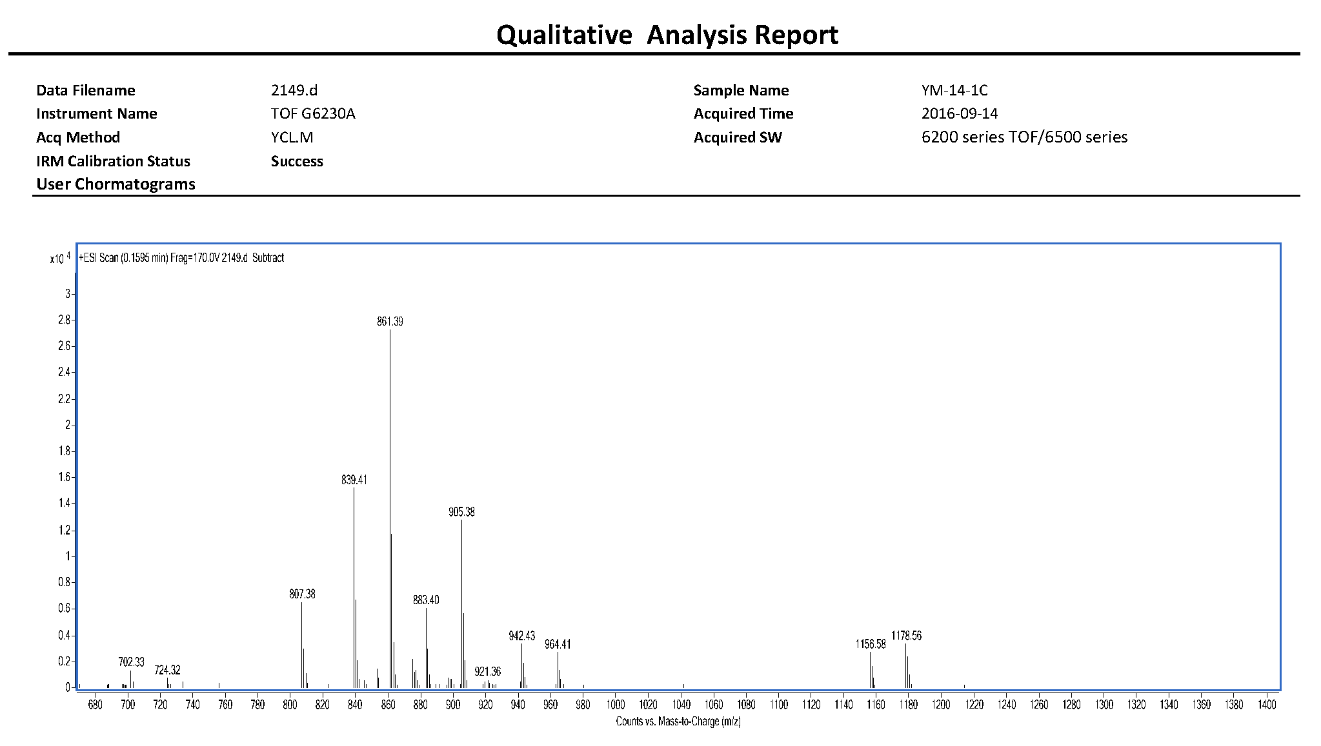


The MS spectrum of Compd. **14**


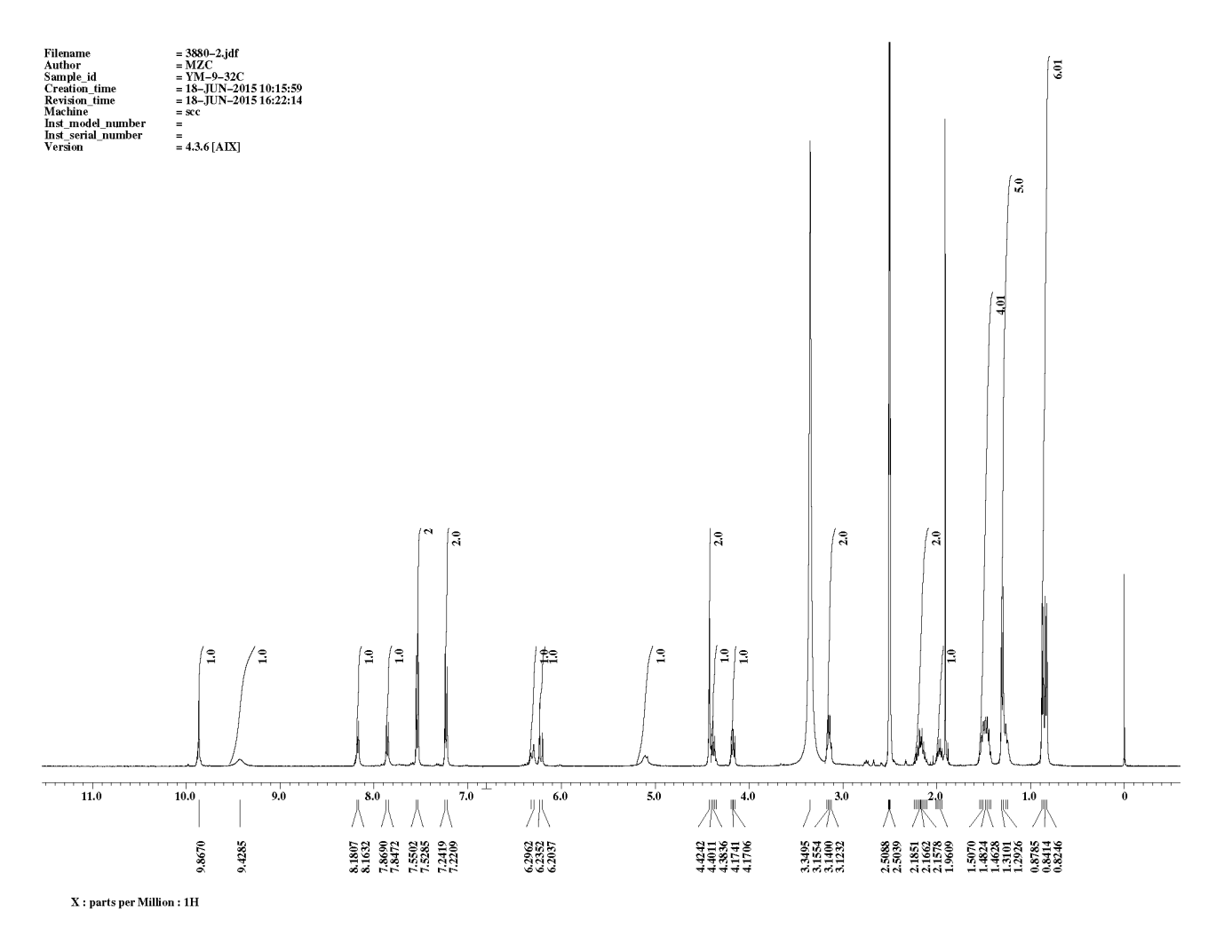


The ^1^H-NMR spectrum of Compd. **S1**


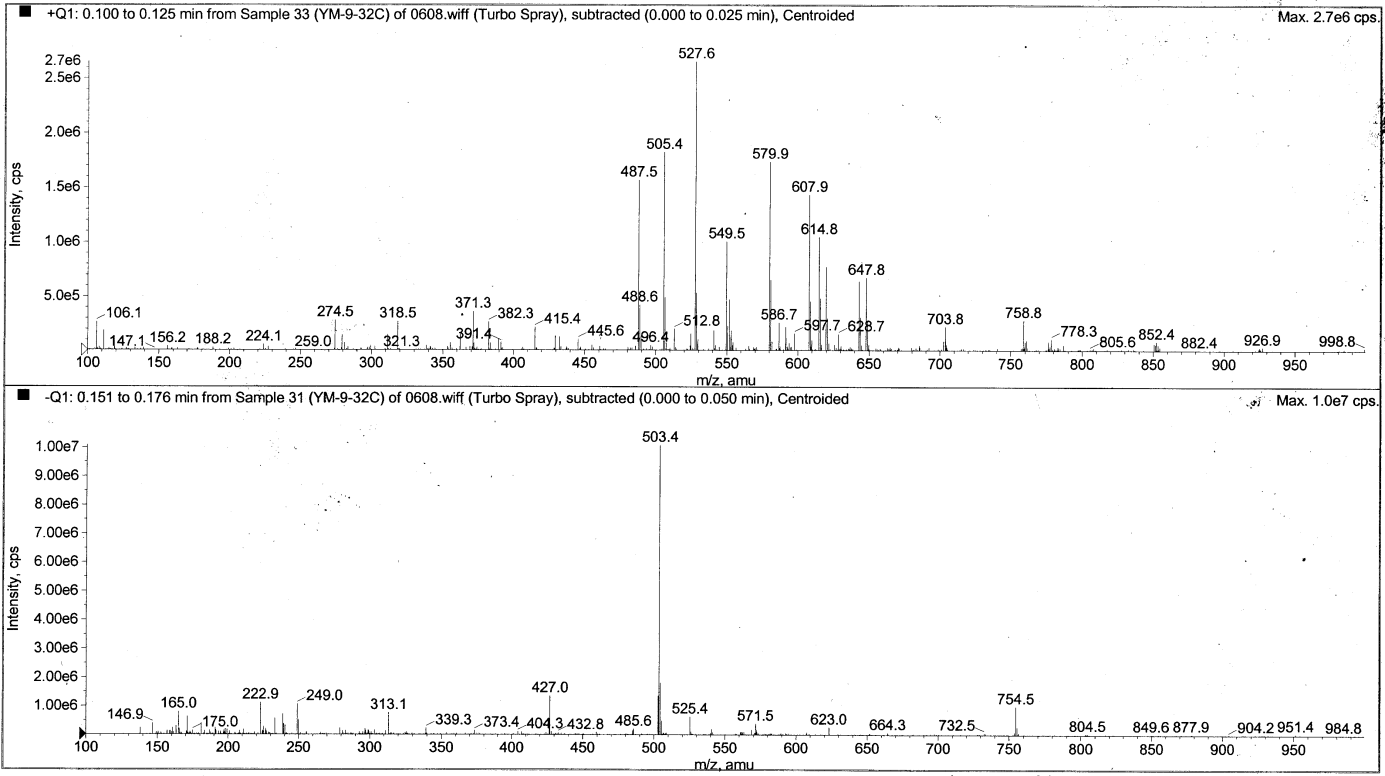


The MS spectrum of Compd. **S1**


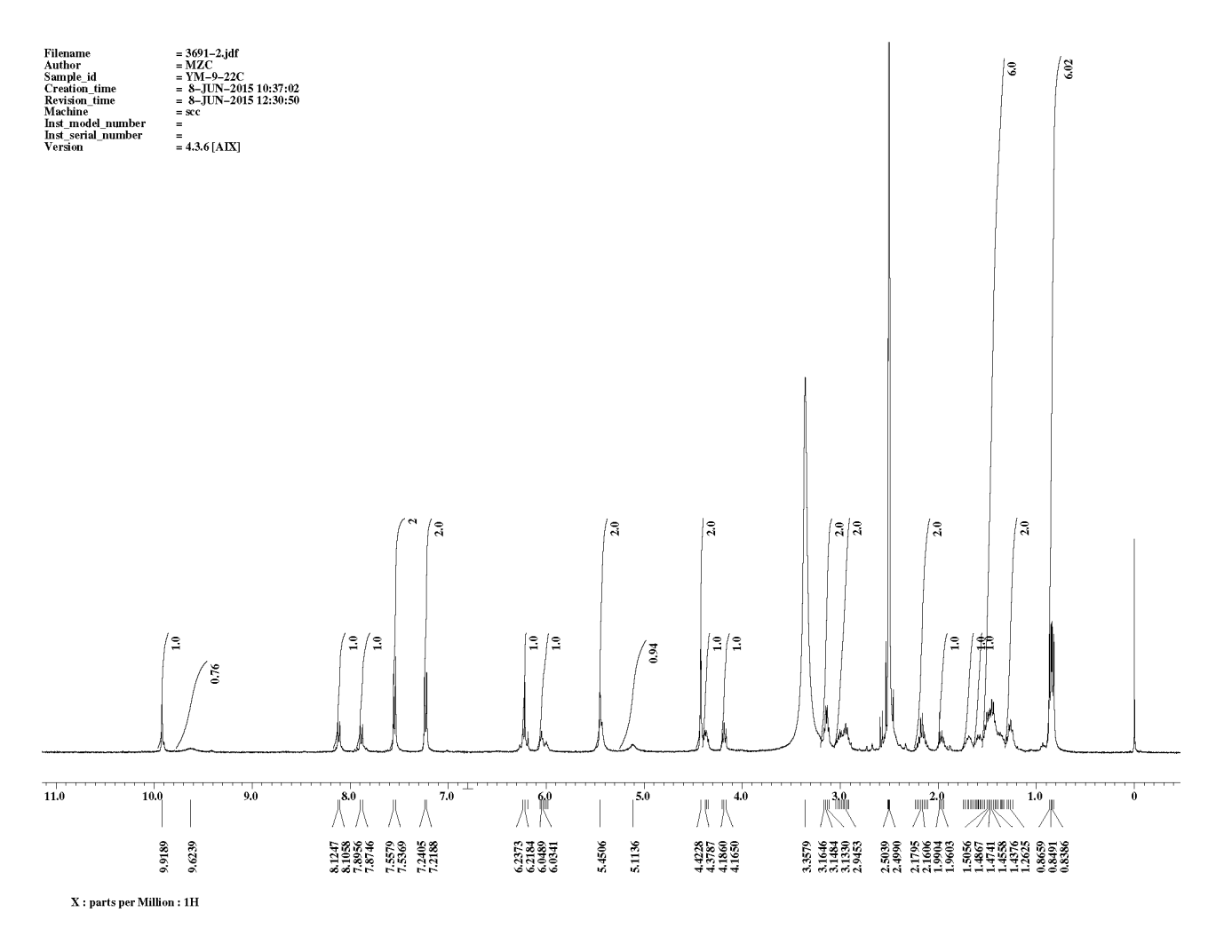


The ^1^H-NMR spectrum of Compd. **S2**


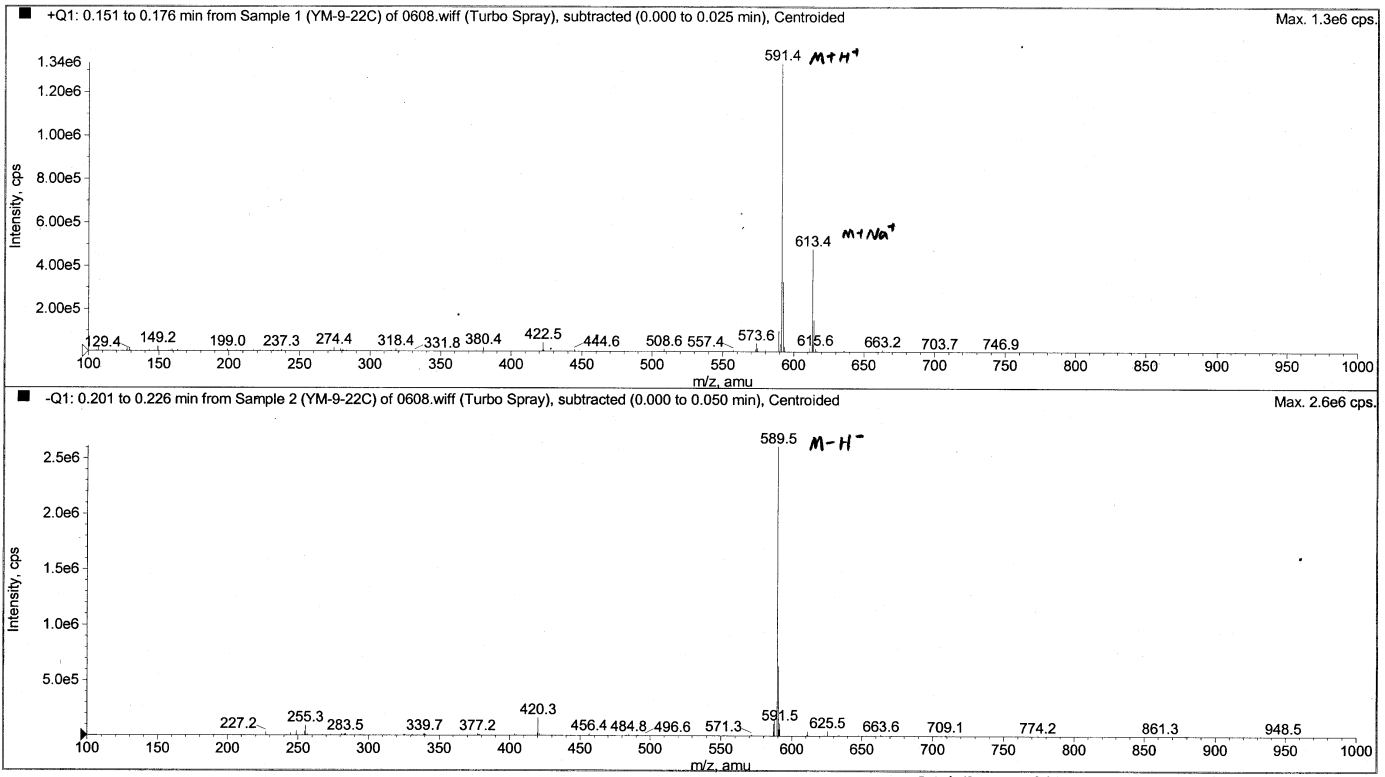


The MS spectrum of Compd. **S2**


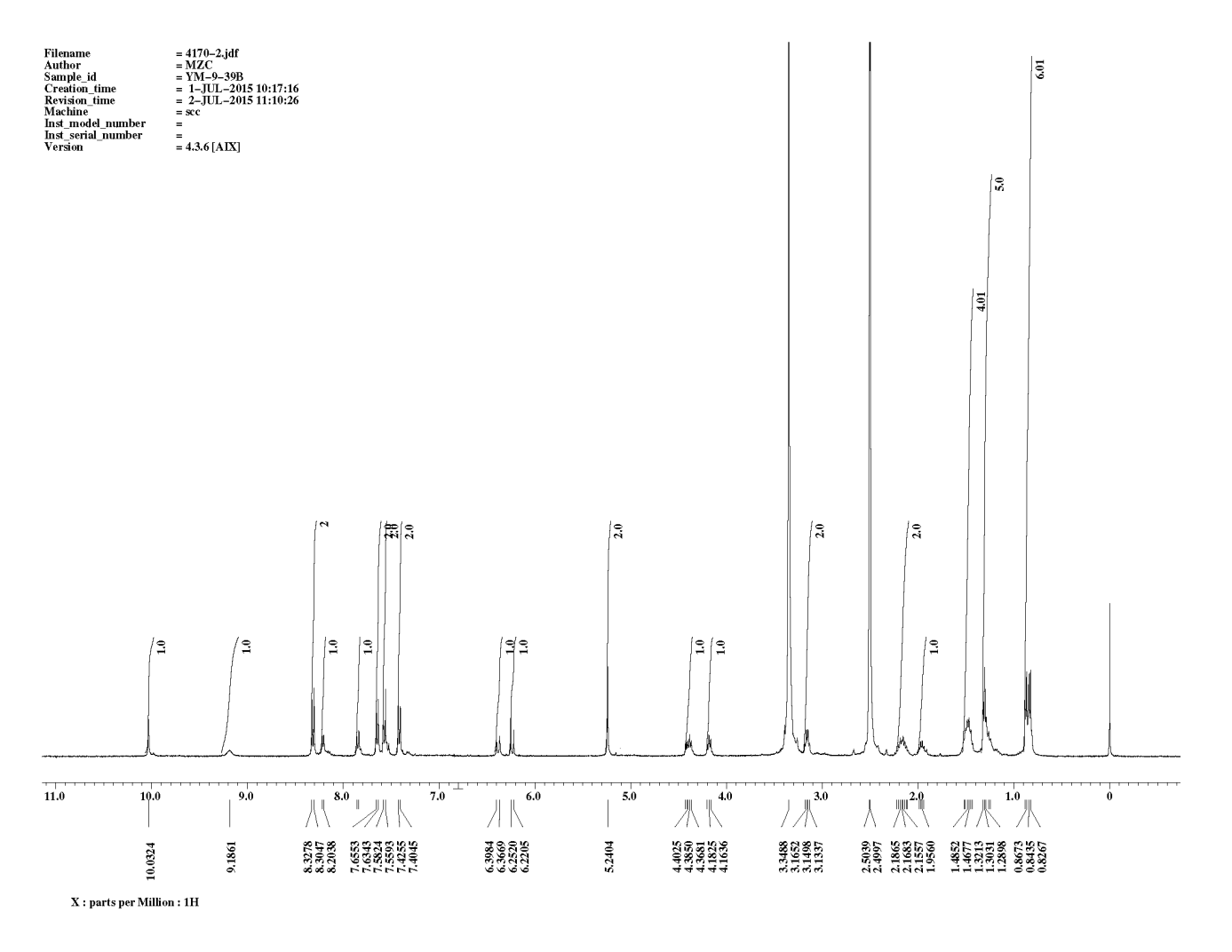


The ^1^H-NMR spectrum of Compd. **S3**


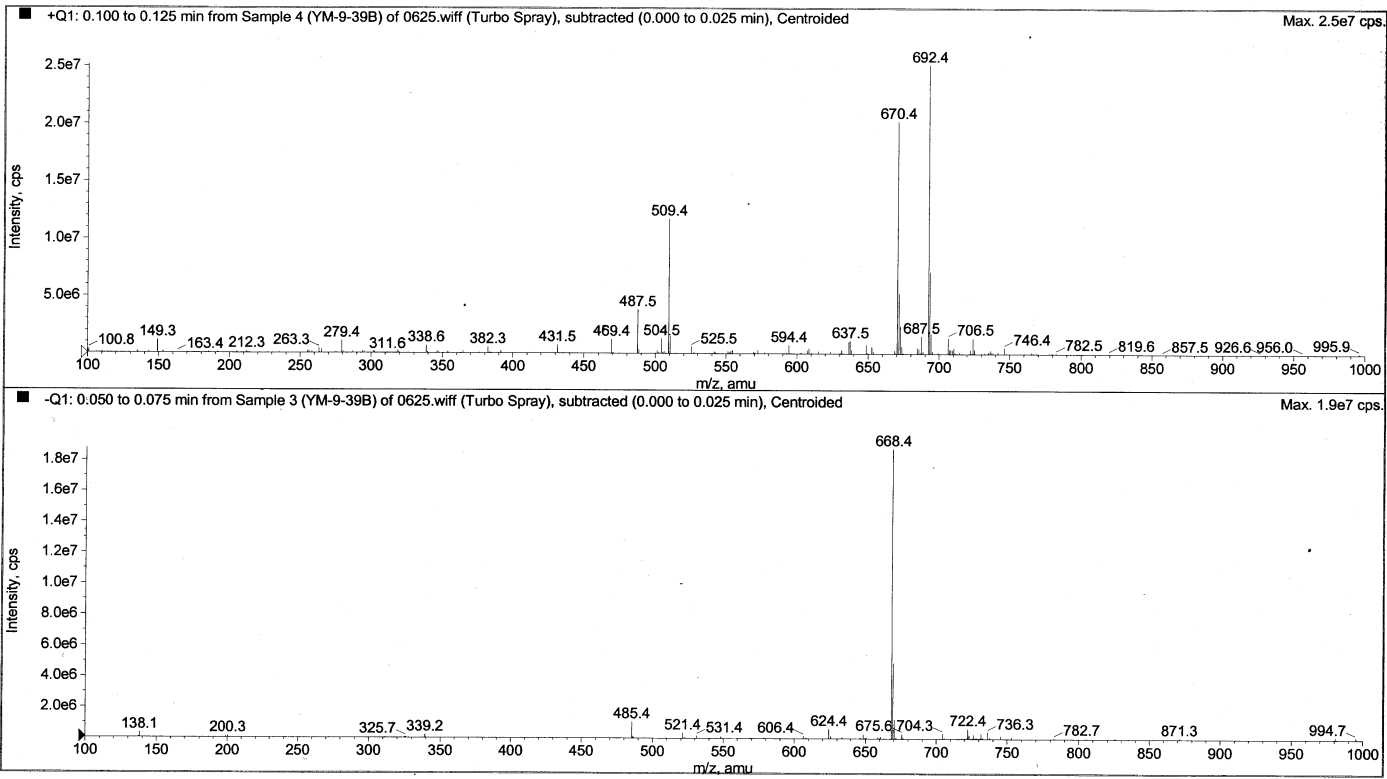


The MS spectrum of Compd. **S3**


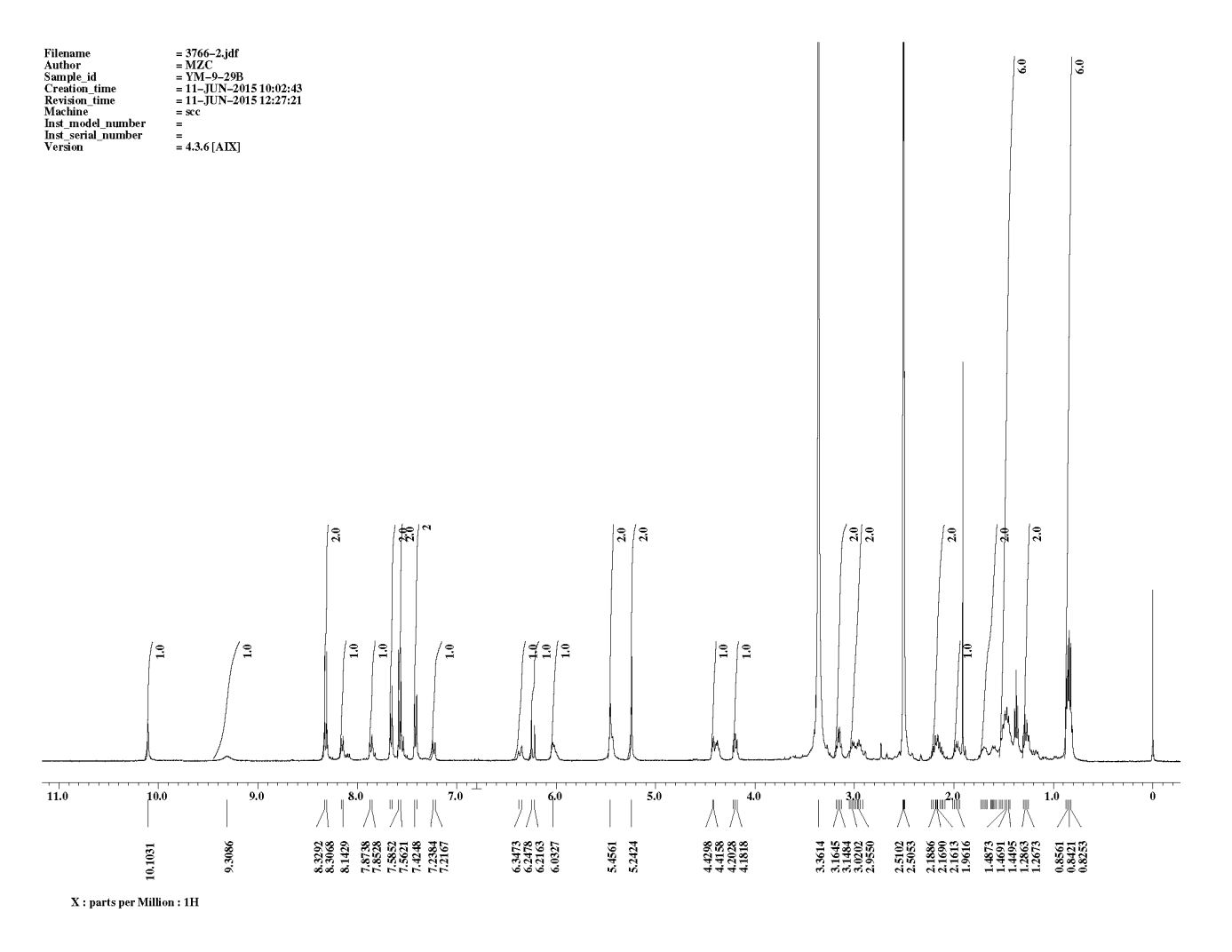


The ^1^H-NMR spectrum of Compd. **S4**


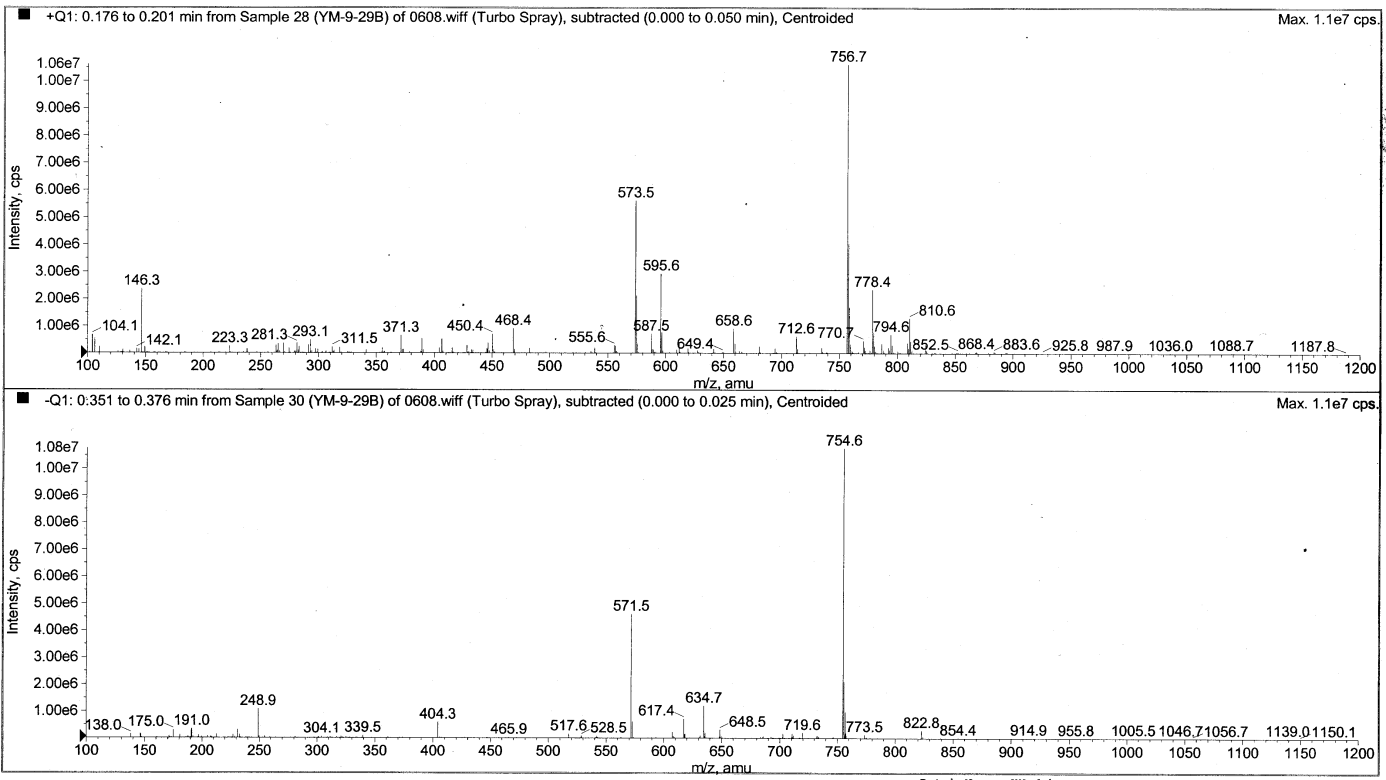


The MS spectrum of Compd. **S4**


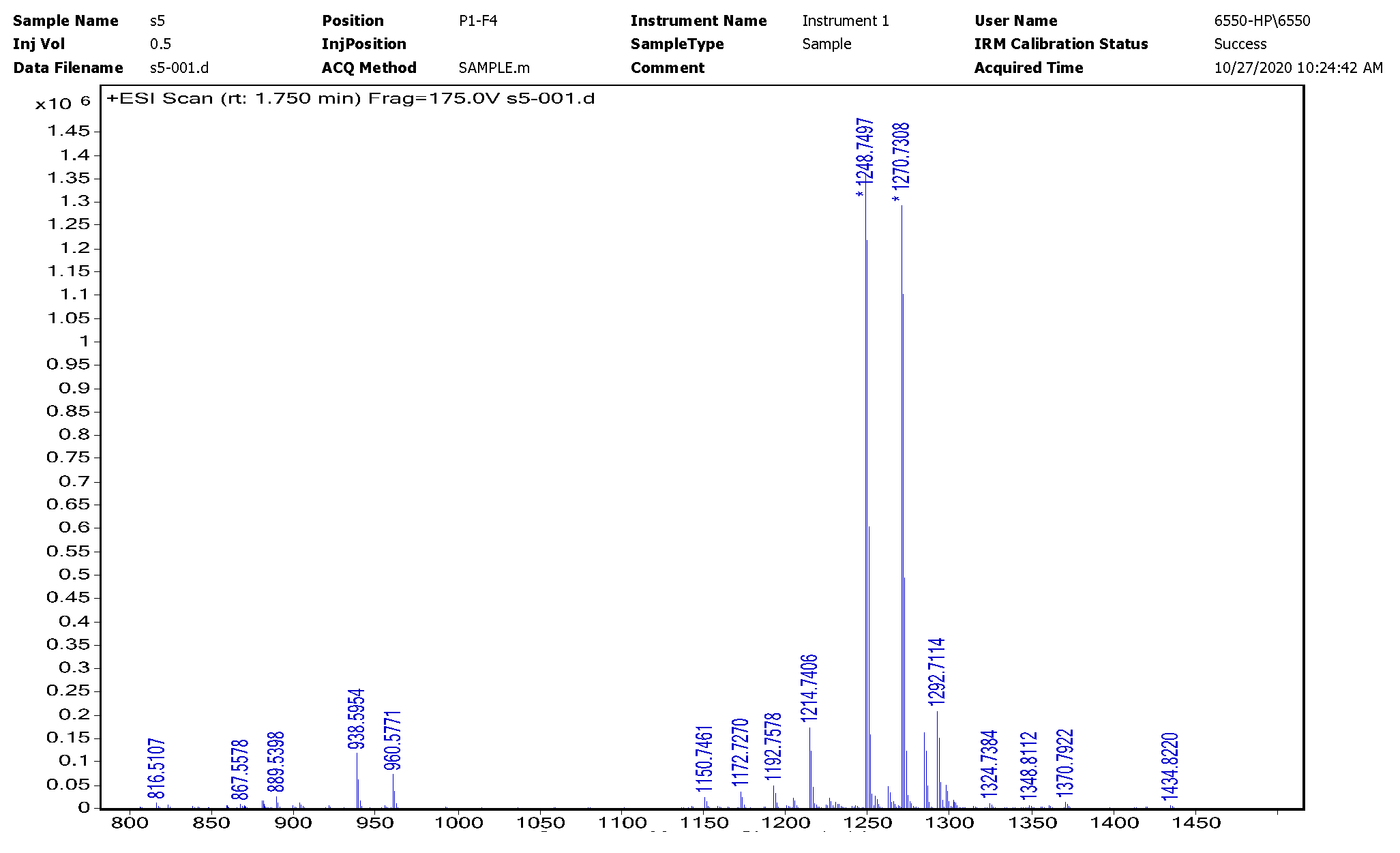


The HR-MS spectrum of Compd. **S5**


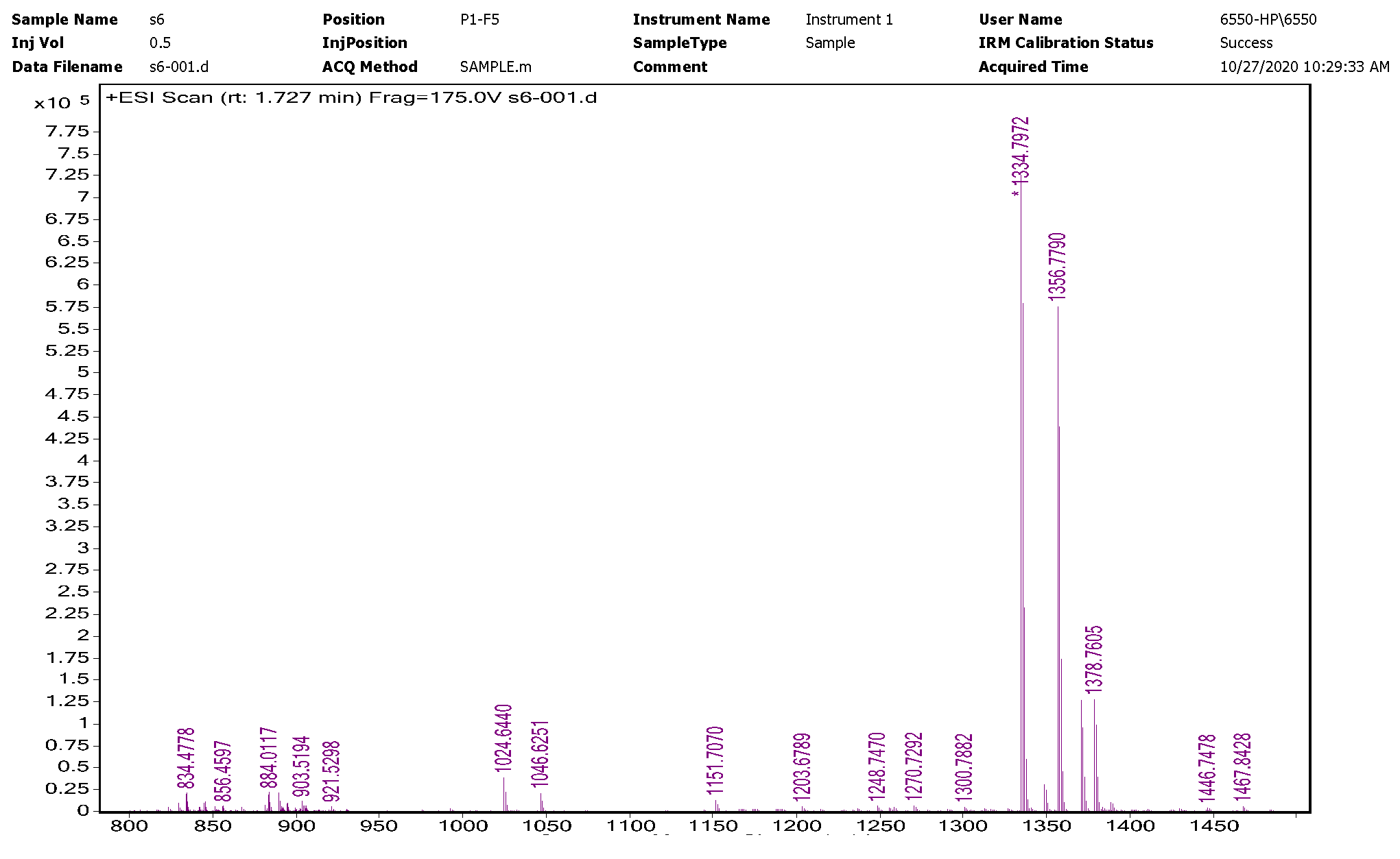


The HR-MS spectrum of Compd. **S6**


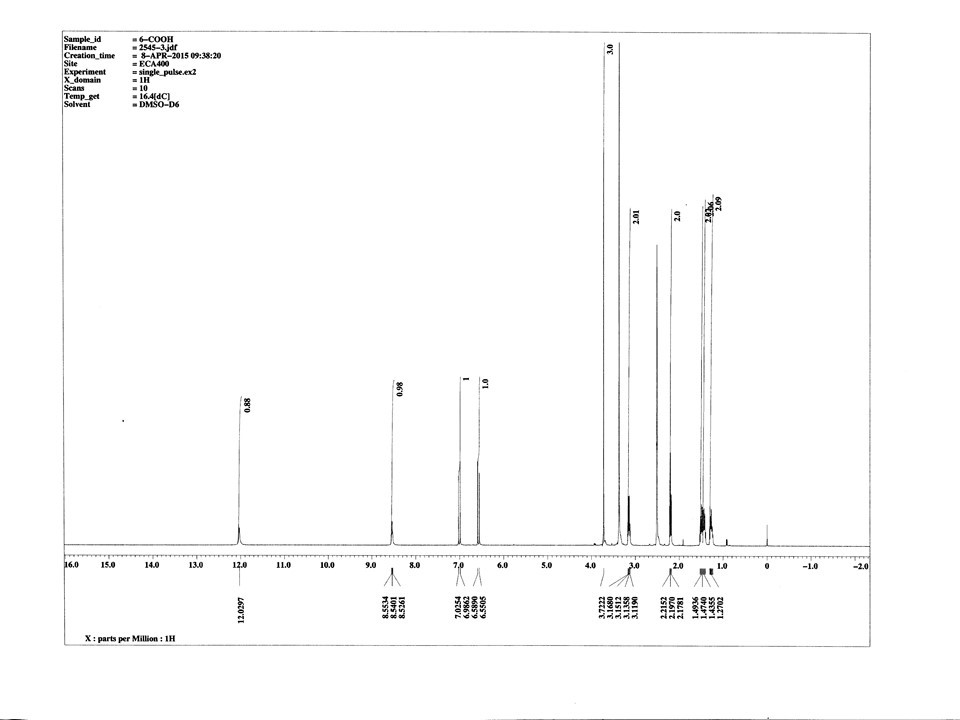


The ^1^H-NMR spectrum of Compd. **S7**


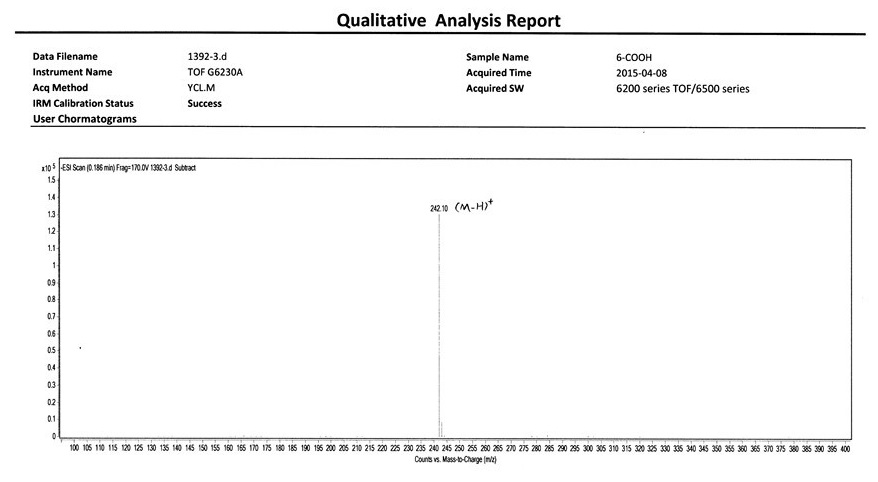


The MS spectrum of Compd. **S7**


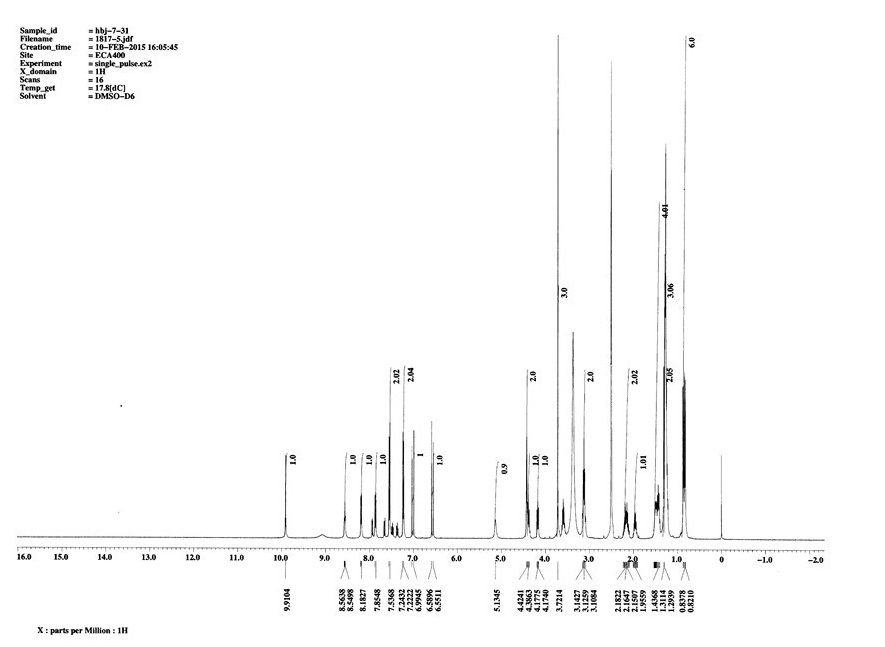


The ^1^H-NMR spectrum of Compd. **S8**


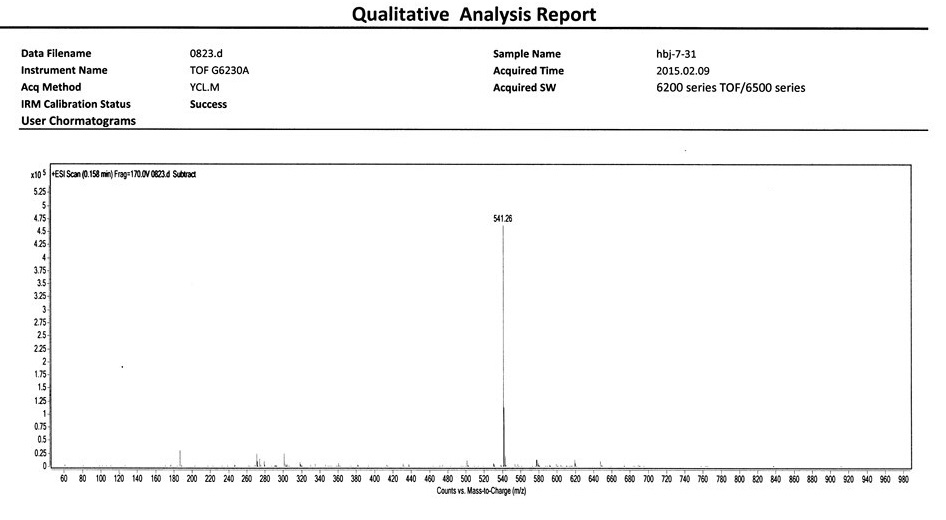


The MS spectrum of Compd. **S8**


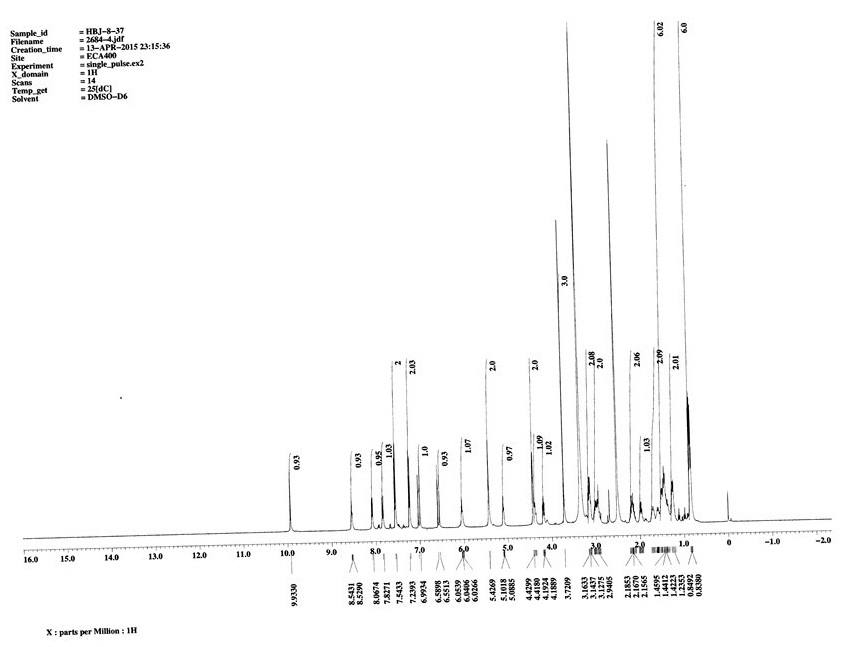


The ^1^H-NMR spectrum of Compd. **S9**


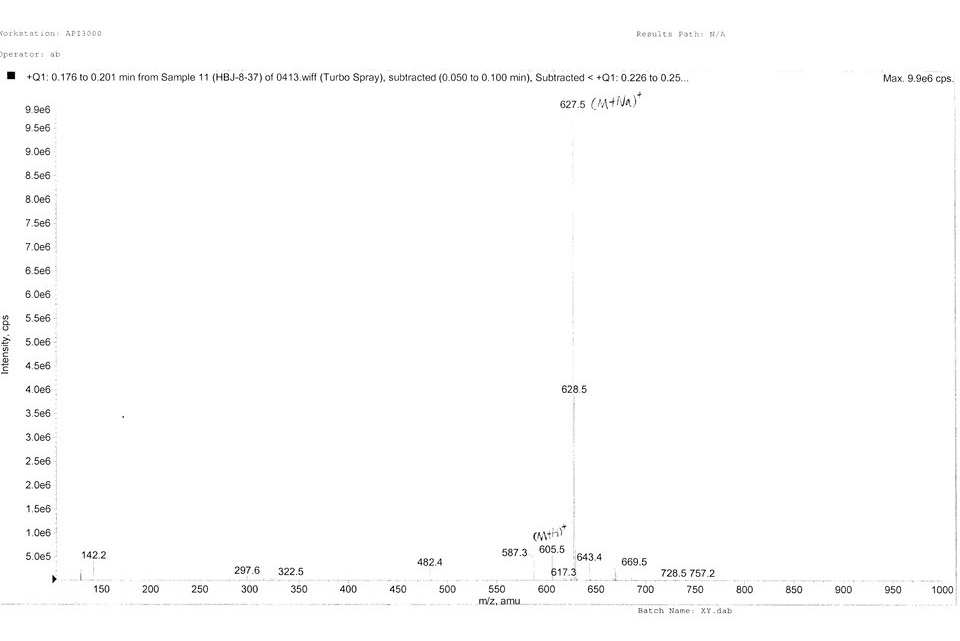


The MS spectrum of Compd. **S9**


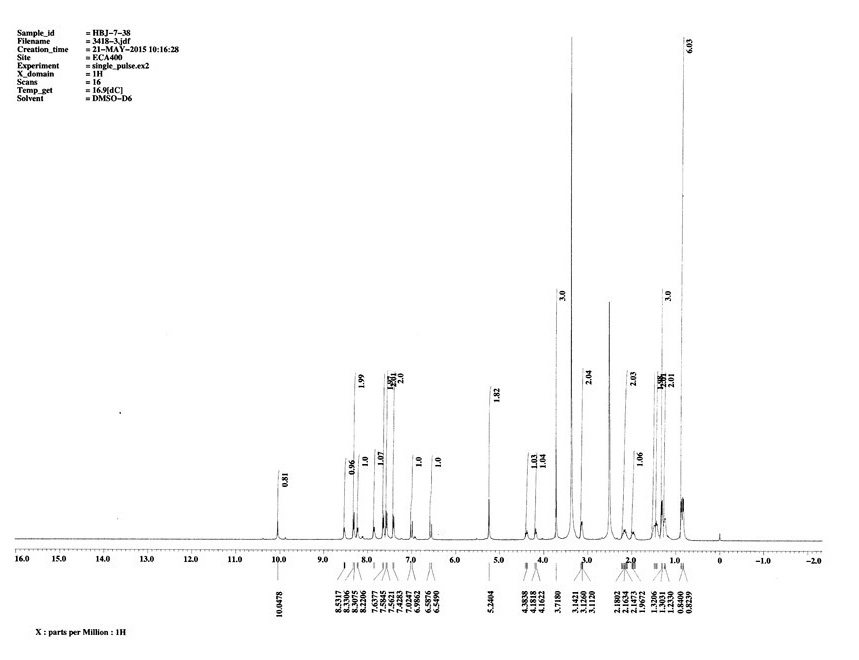


The ^1^H-NMR spectrum of Compd. **S10**


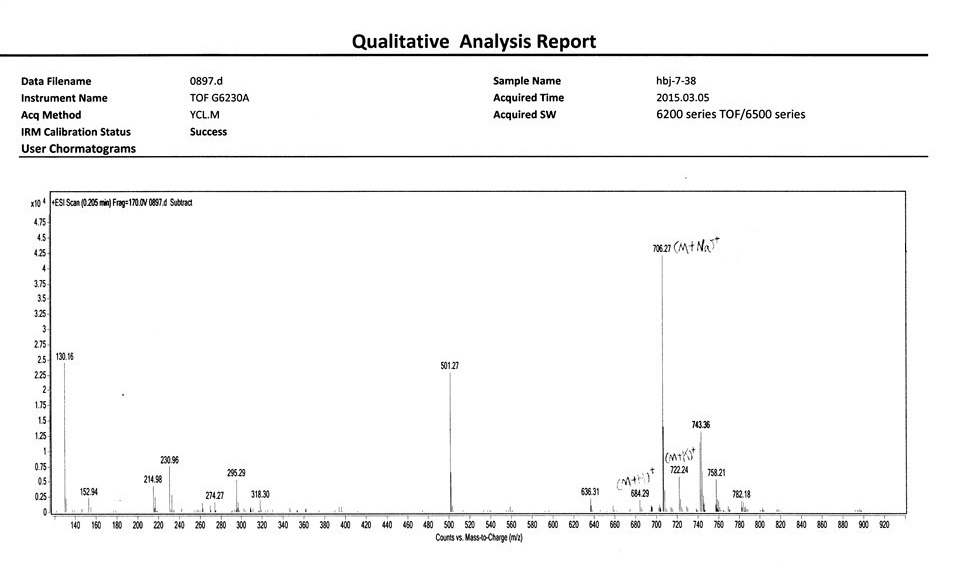


The MS spectrum of Compd. **S10**


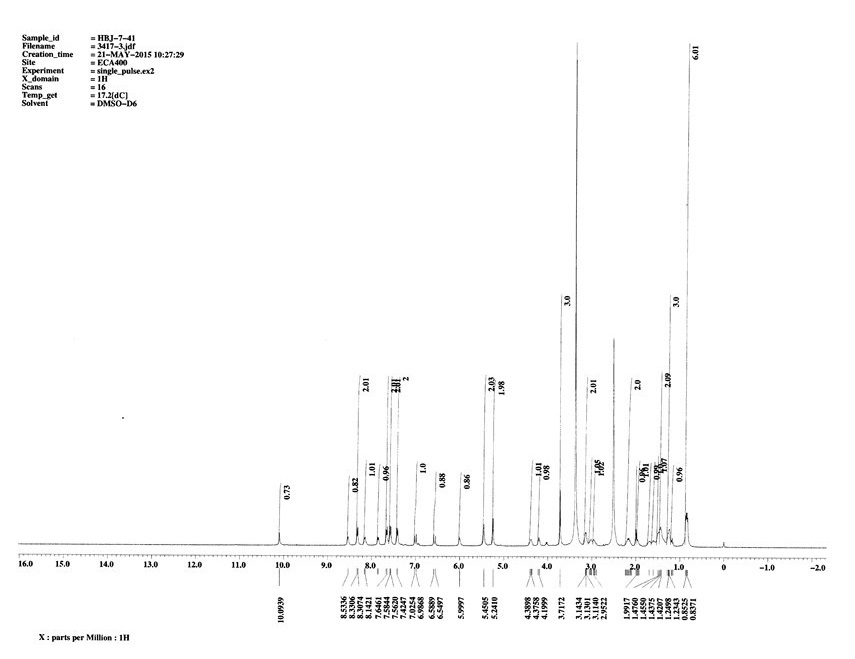


The ^1^H-NMR spectrum of Compd. **S11**


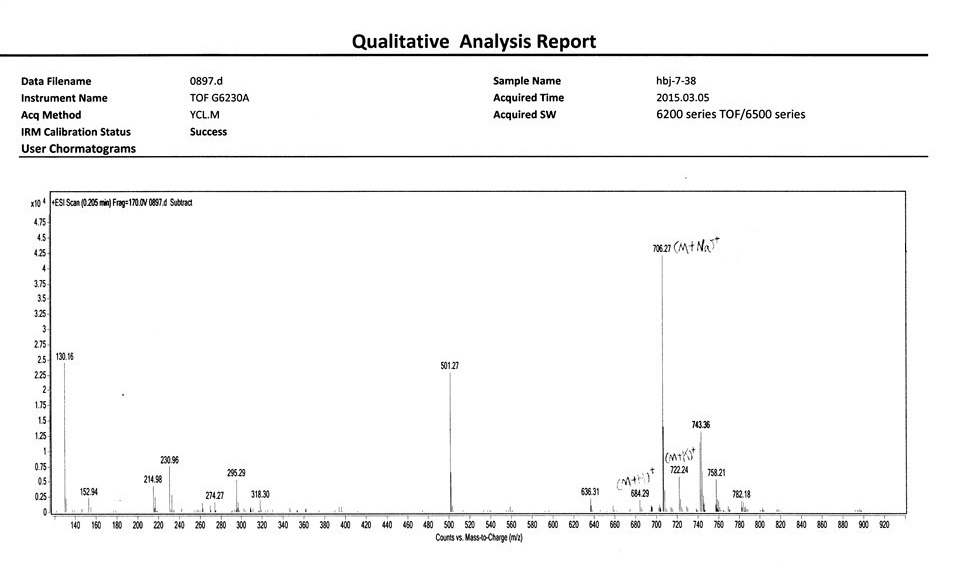


The MS spectrum of Compd. **S11**


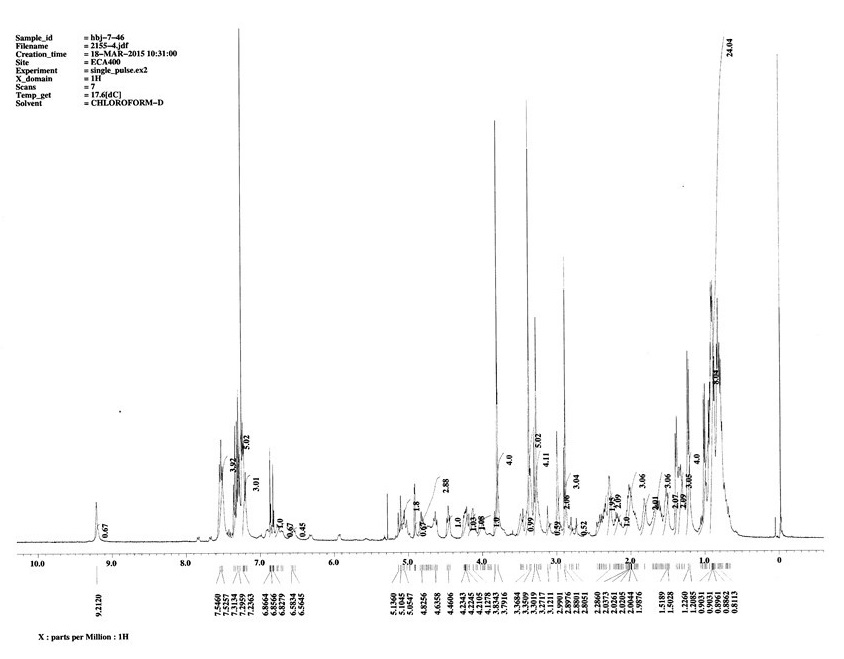


The ^1^H-NMR spectrum of Compd. **S12**


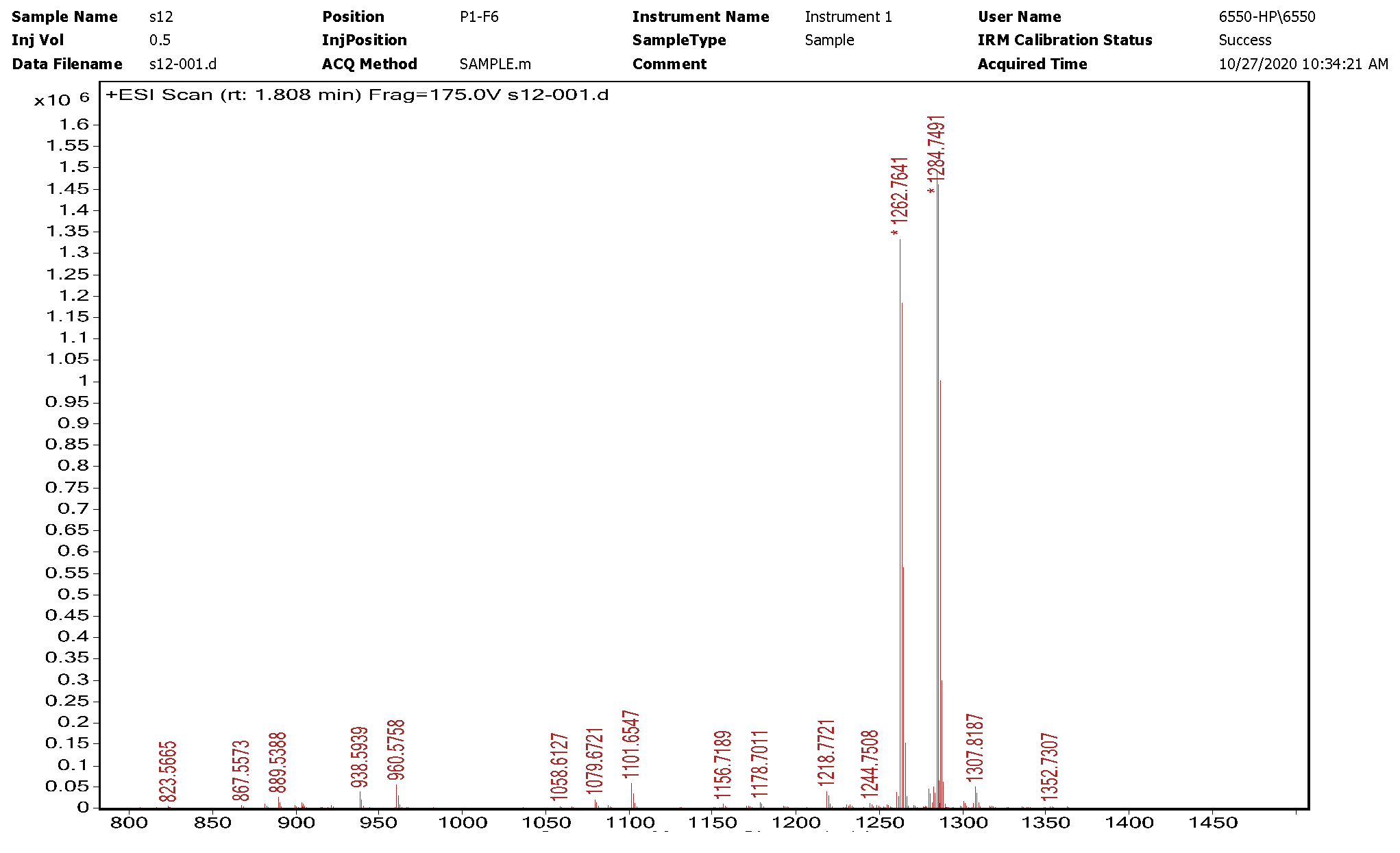


The HR-MS spectrum of Compd. **S12**


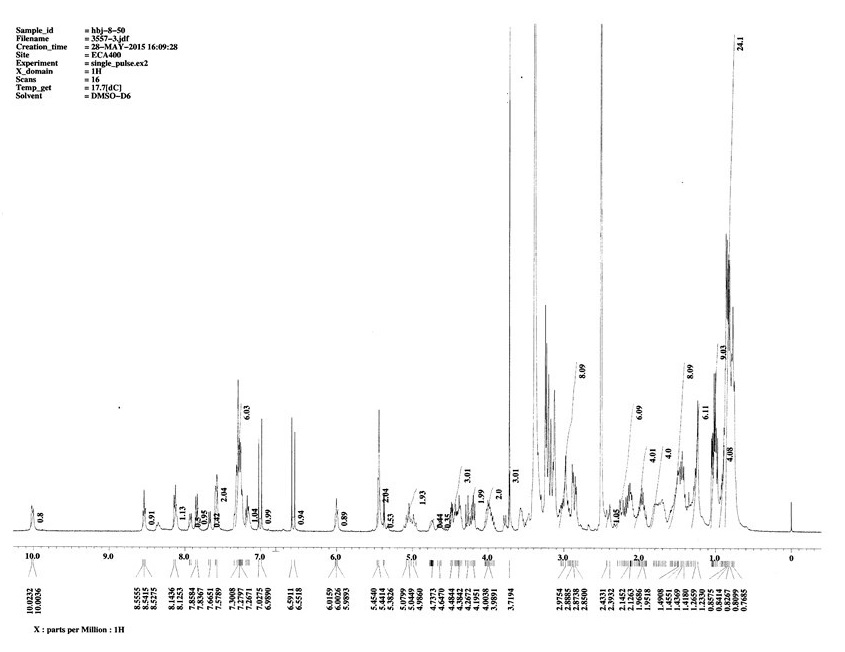


The ^1^H-NMR spectrum of Compd. **S13**


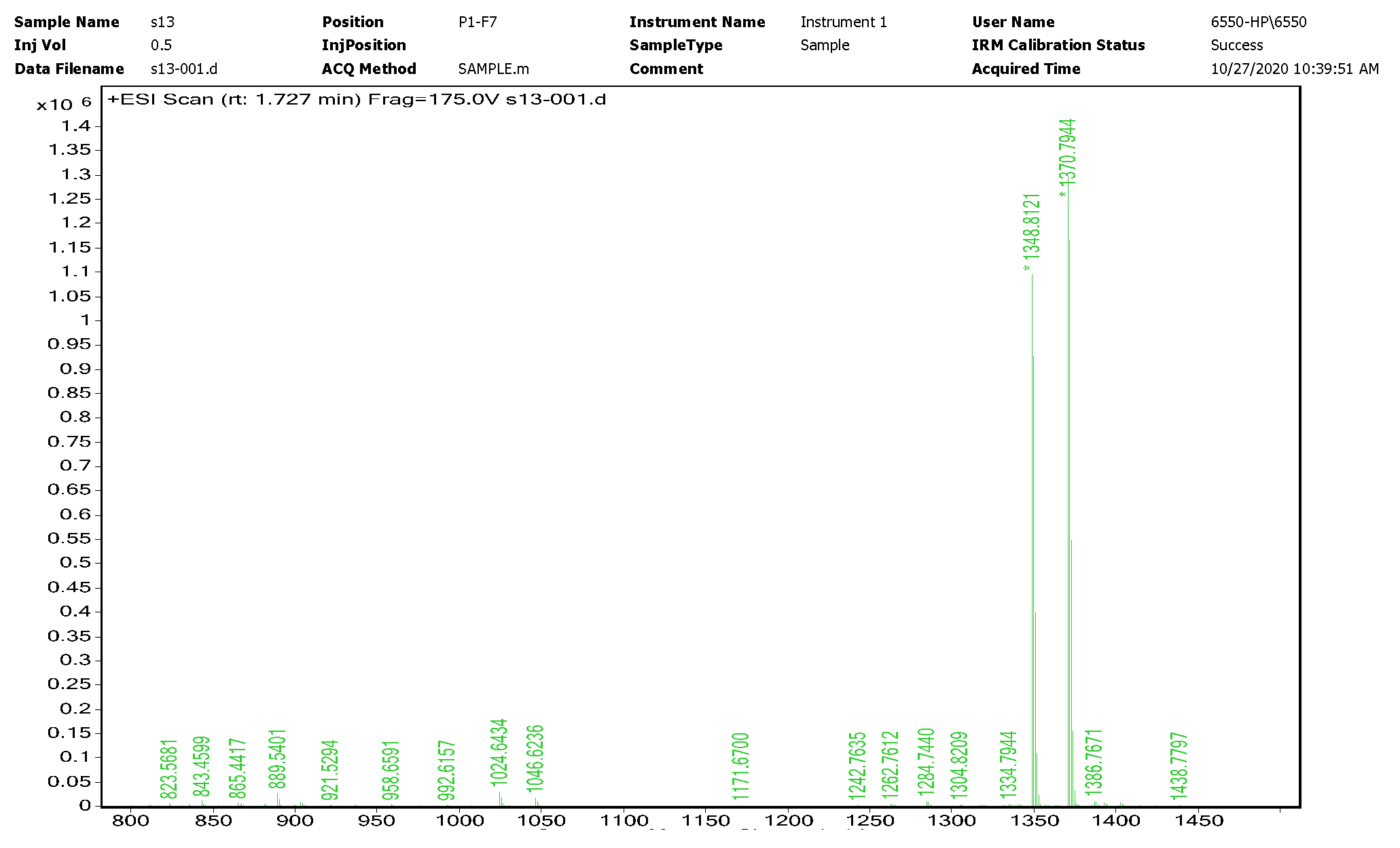


The HR-MS spectrum of Compd. **S13**


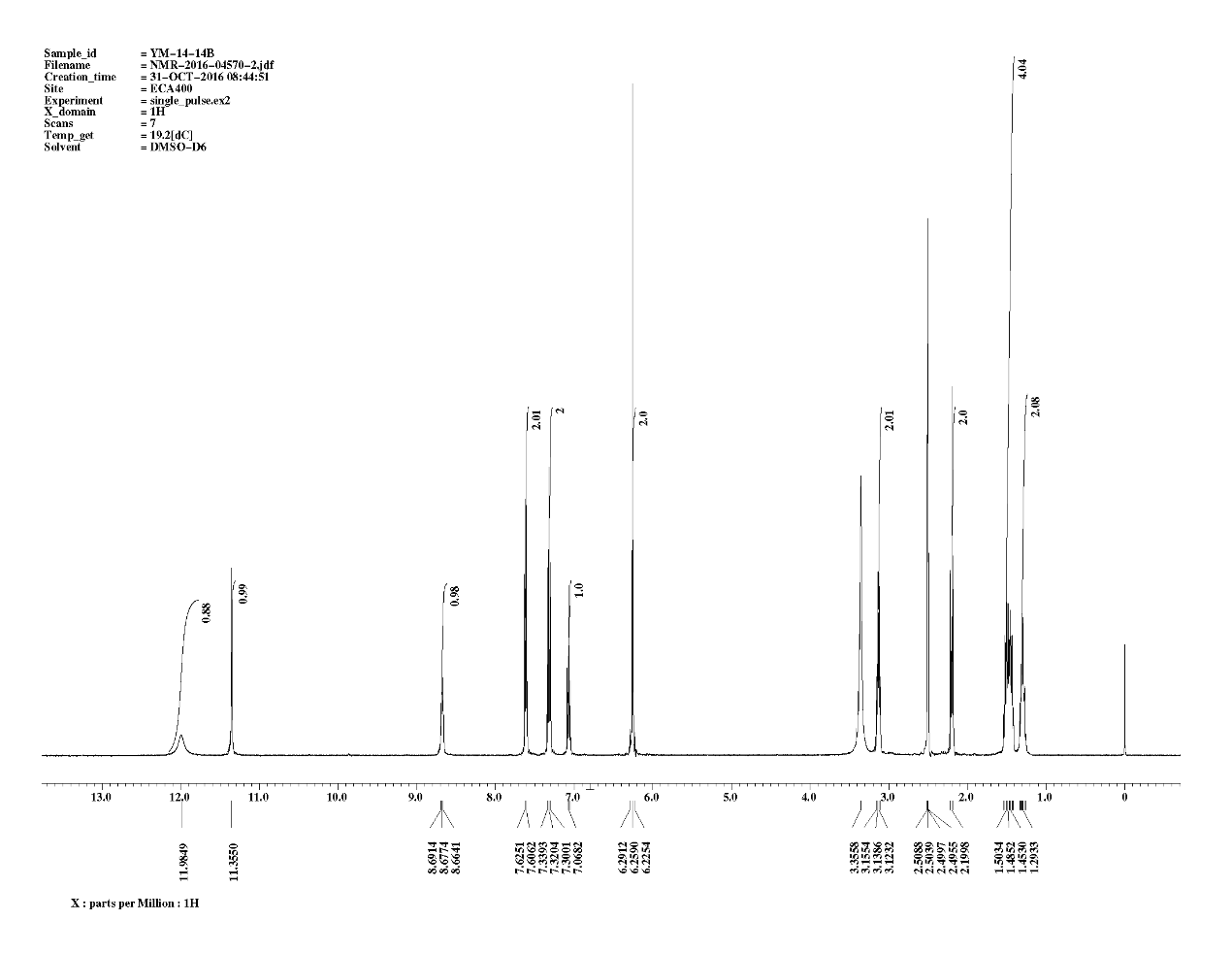


The ^1^H-NMR spectrum of Compd. **S14**


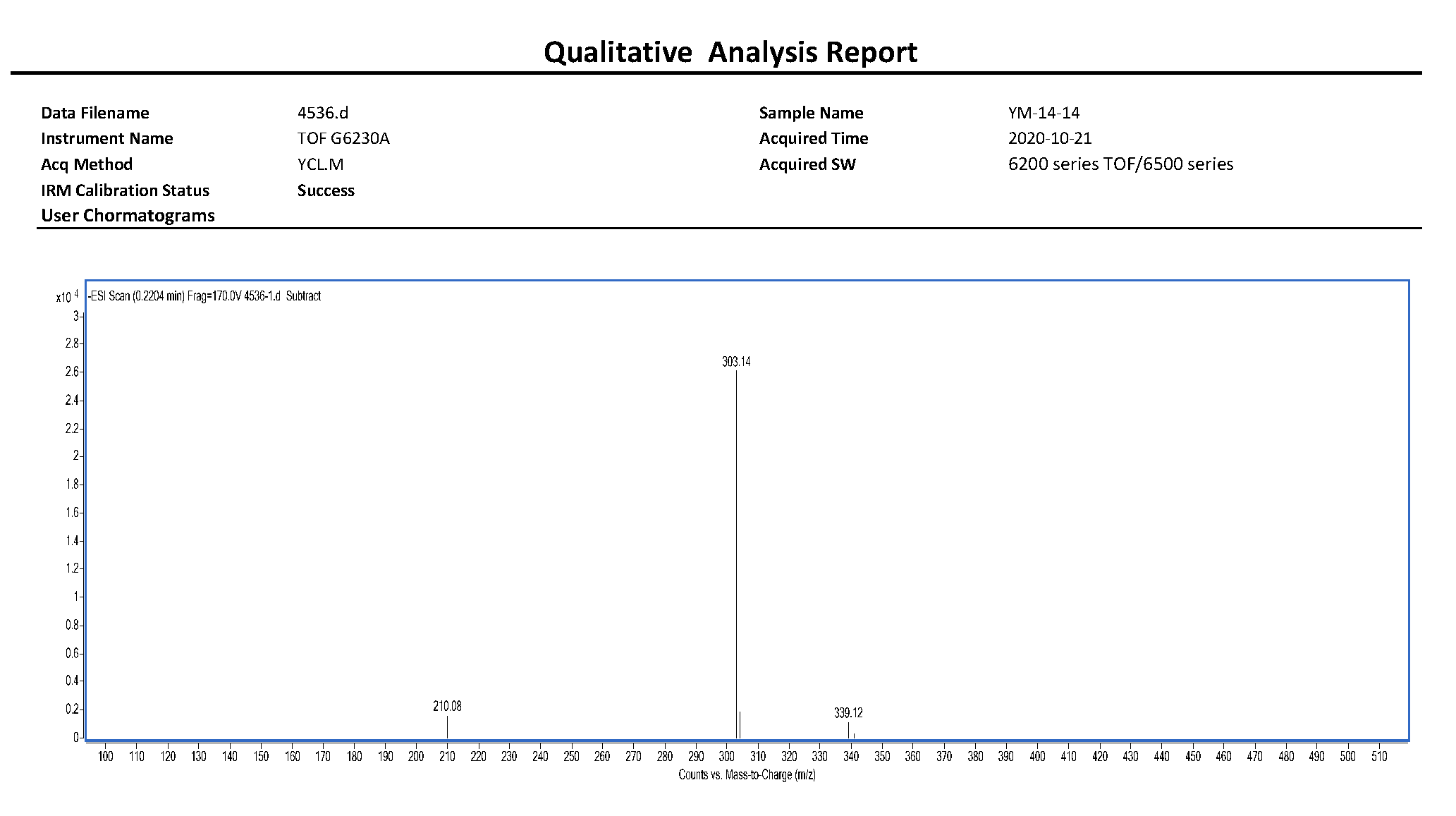


The MS spectrum of Compd. **S14**


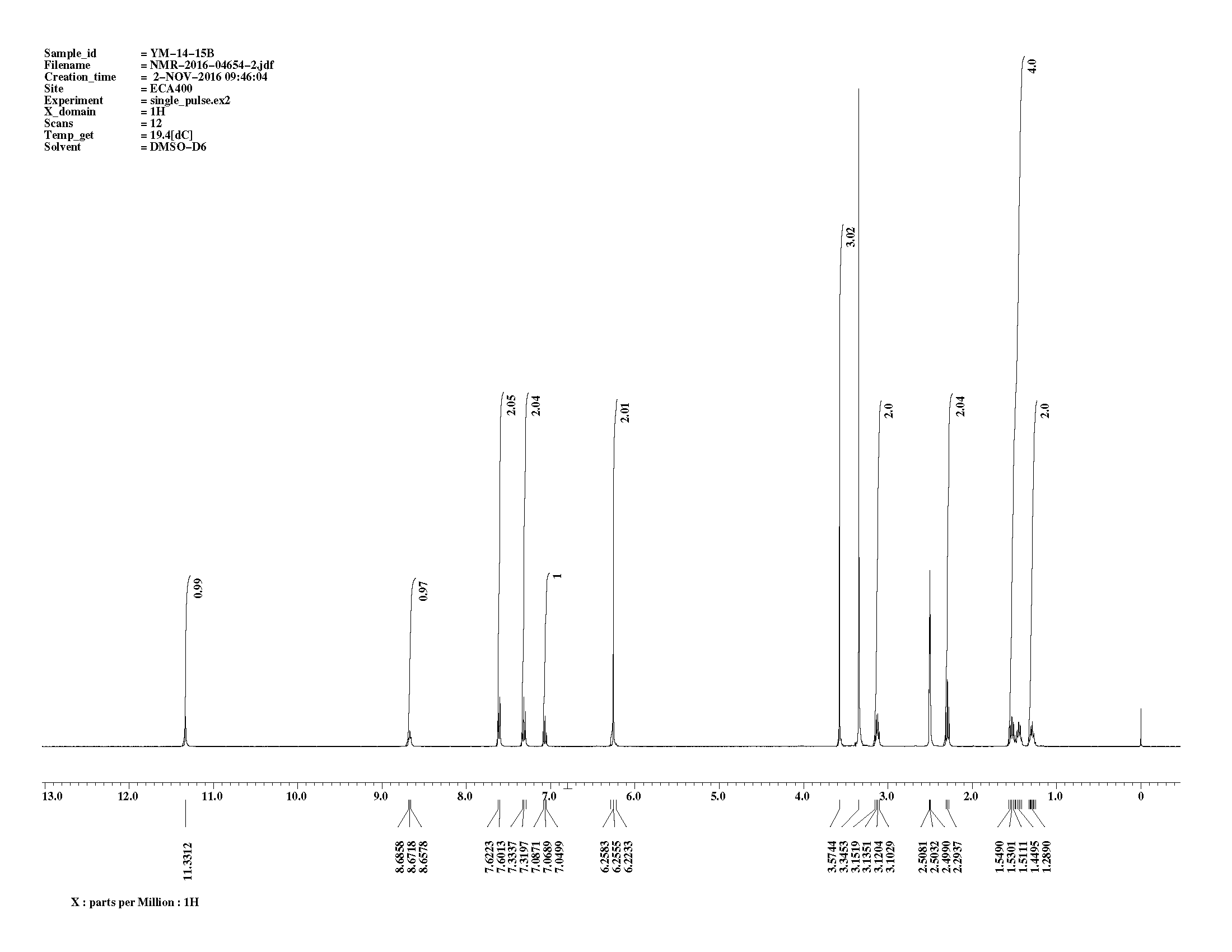


The ^1^H-NMR spectrum of Compd. **S15**


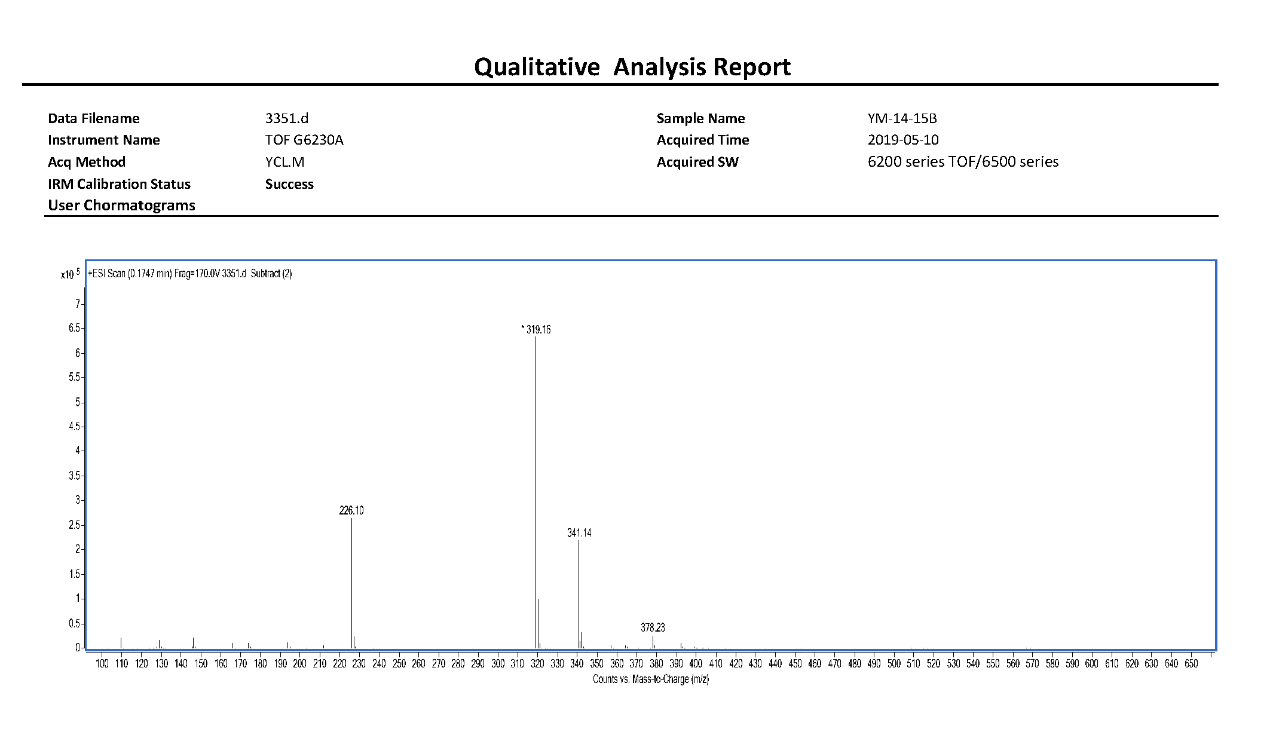


The MS spectrum of Compd. **S15**


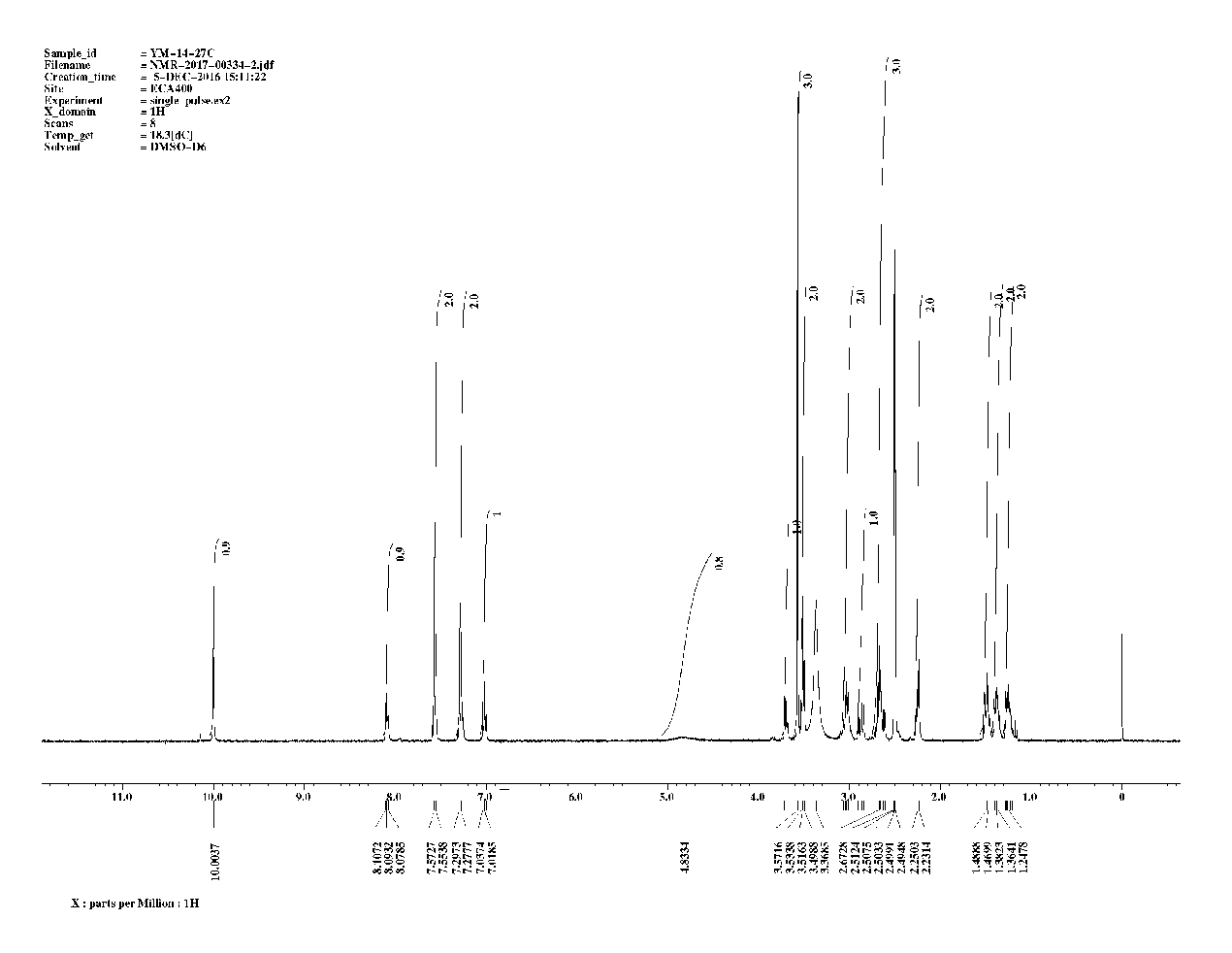


The ^1^H-NMR spectrum of Compd. **S16**


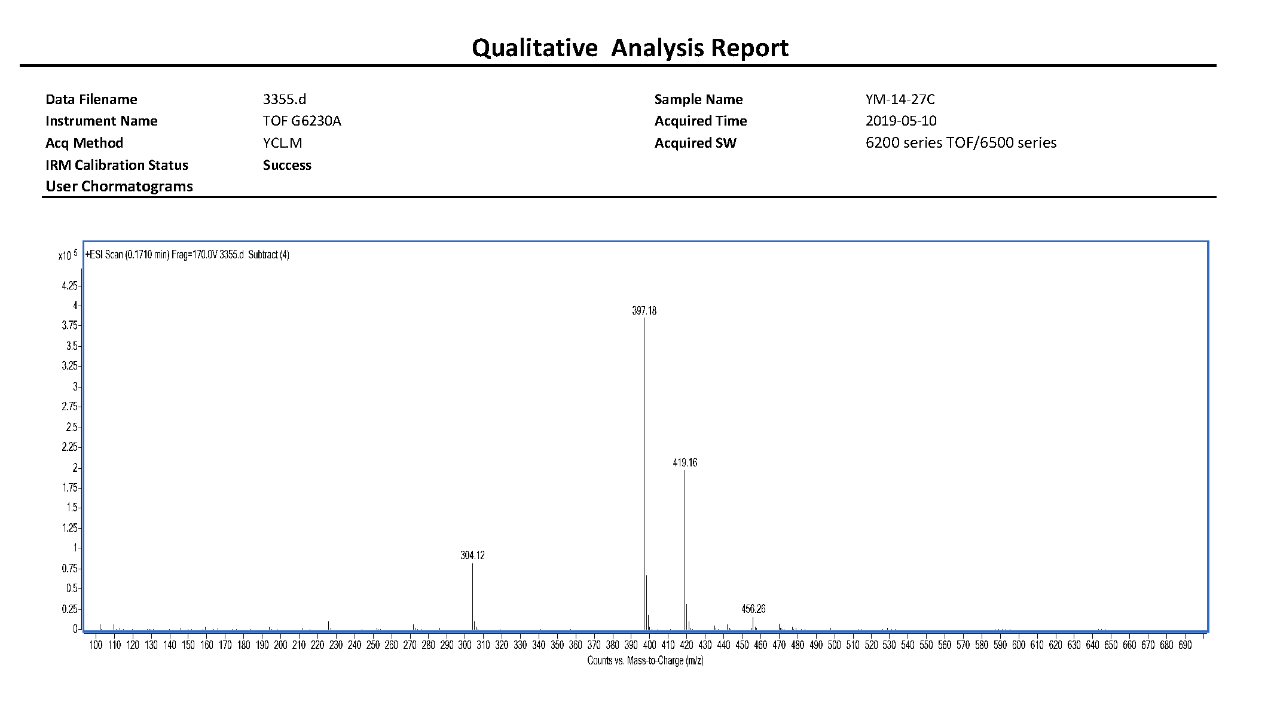


The MS spectrum of Compd. **S16**





The ^1^H-NMR spectrum of Compd. **S17**


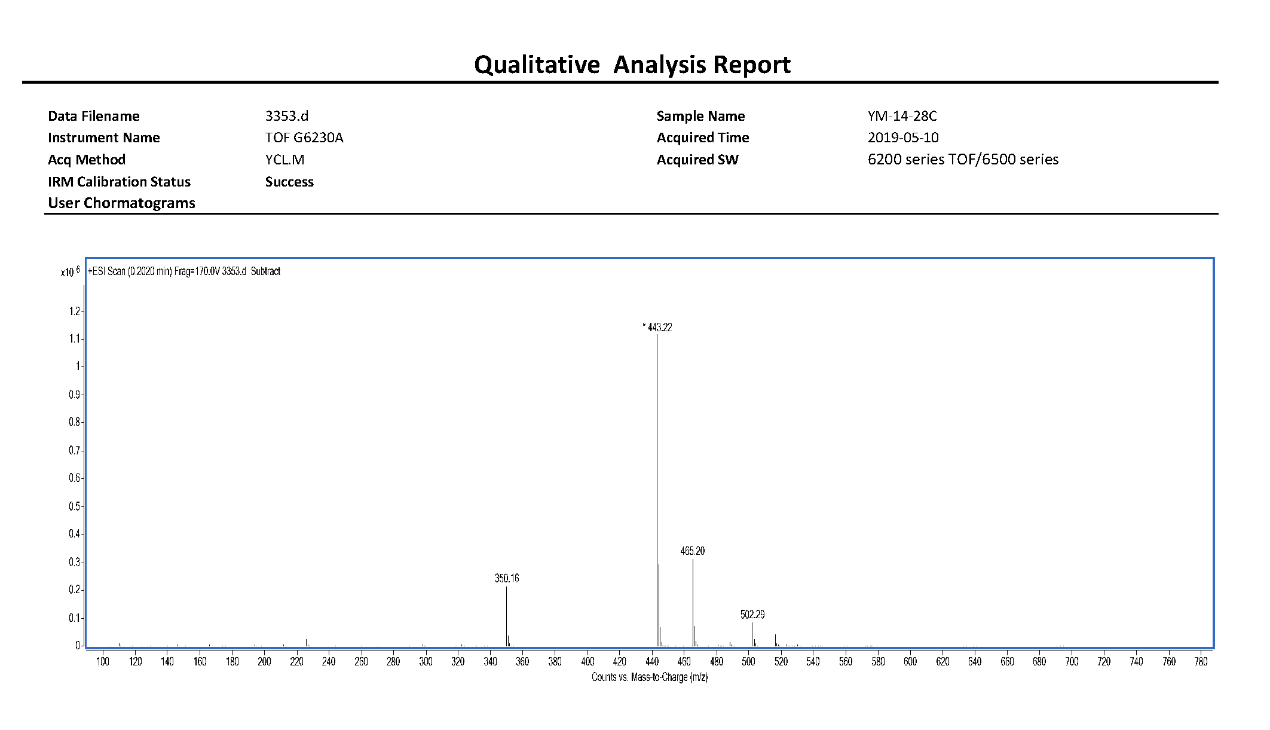


The MS spectrum of Compd. **S17**





The ^1^H-NMR spectrum of Compd. **S18**


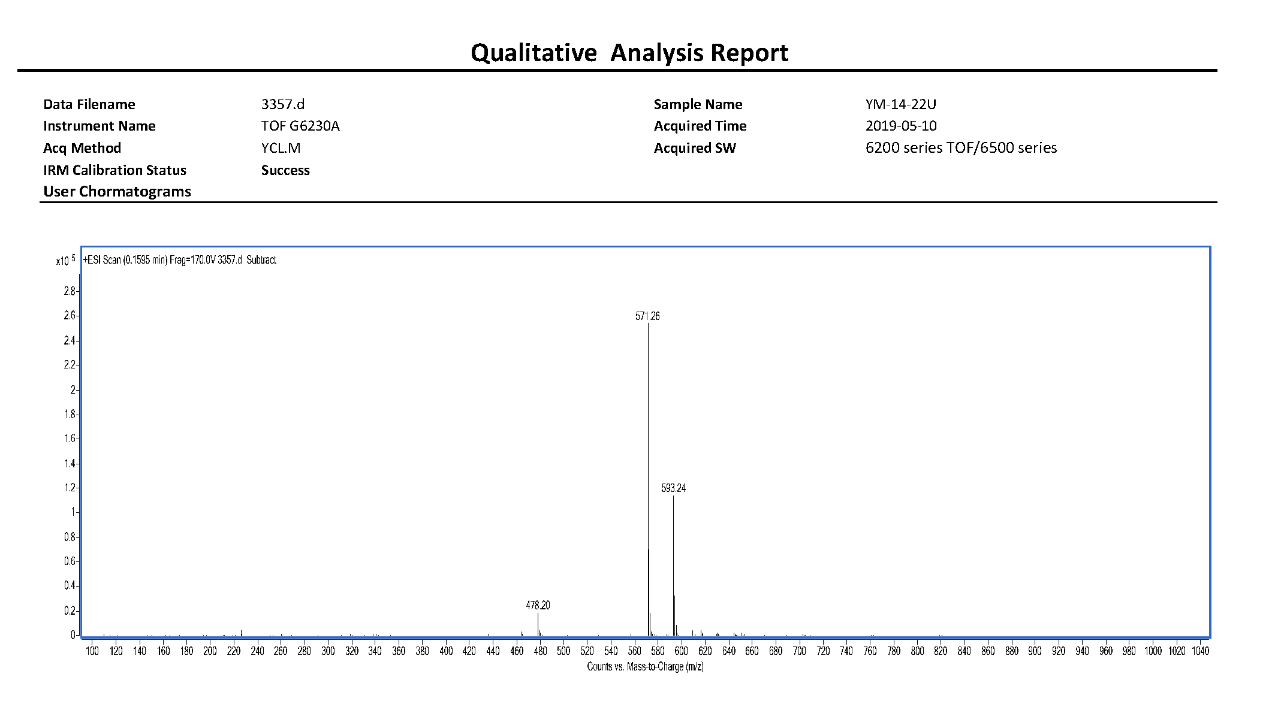


The MS spectrum of Compd. **S18**


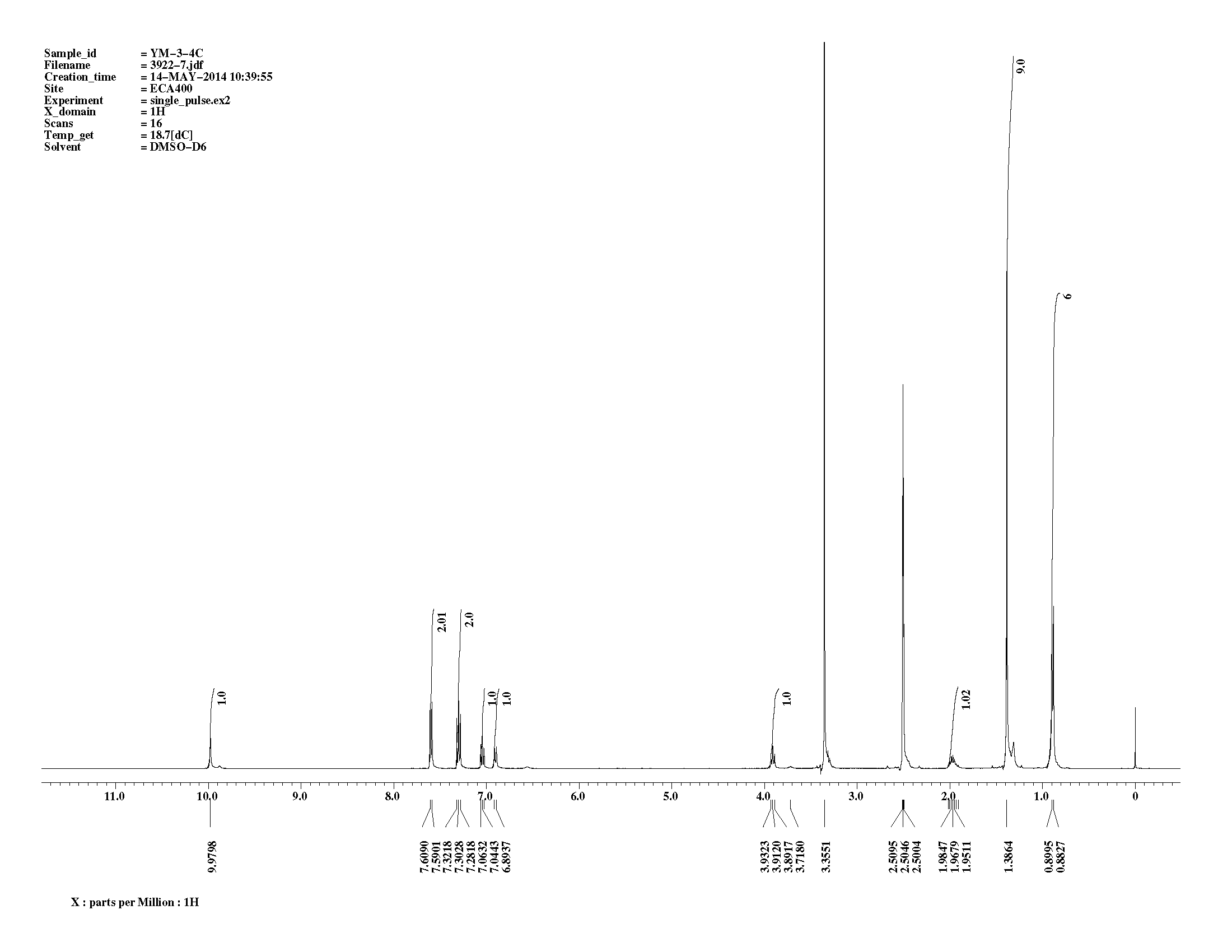


The ^1^H-NMR spectrum of Compd. **S20**





The MS spectrum of Compd. **S20**


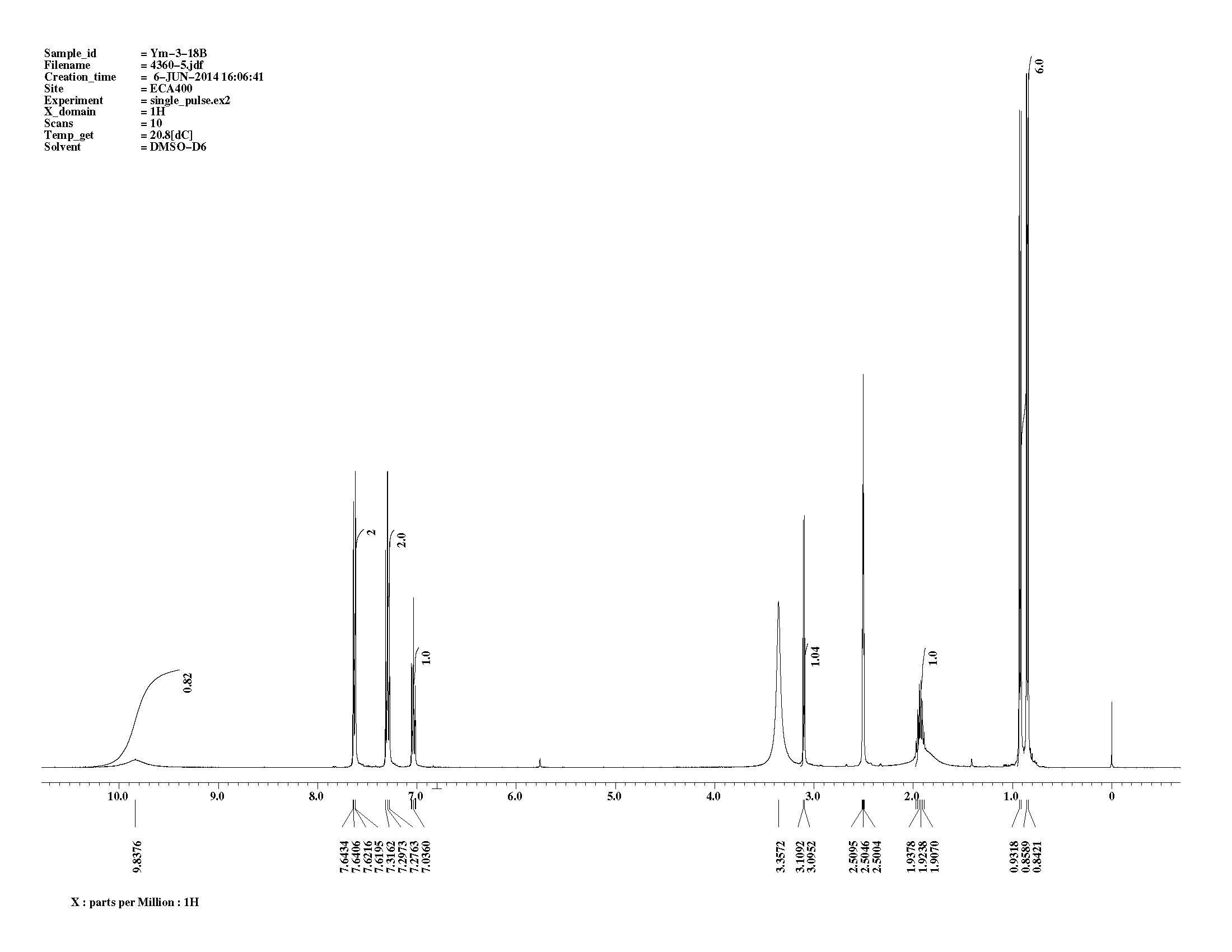


The ^1^H-NMR spectrum of Compd. **S21**





The MS spectrum of Compd. **S21**


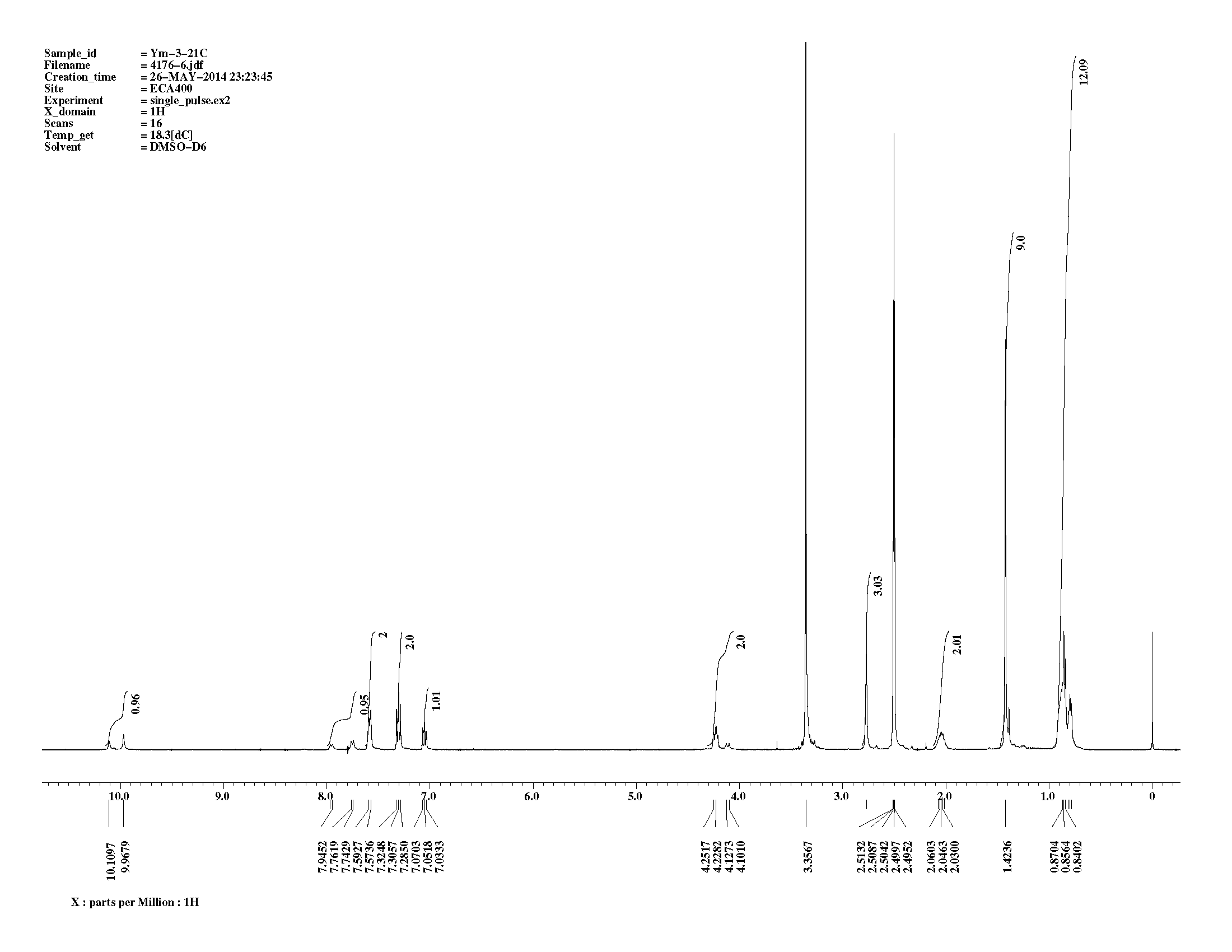


The ^1^H-NMR spectrum of Compd. **S22**





The MS spectrum of Compd. **S22**





The ^1^H-NMR spectrum of Compd. **S23**





The MS spectrum of Compd. **S23**


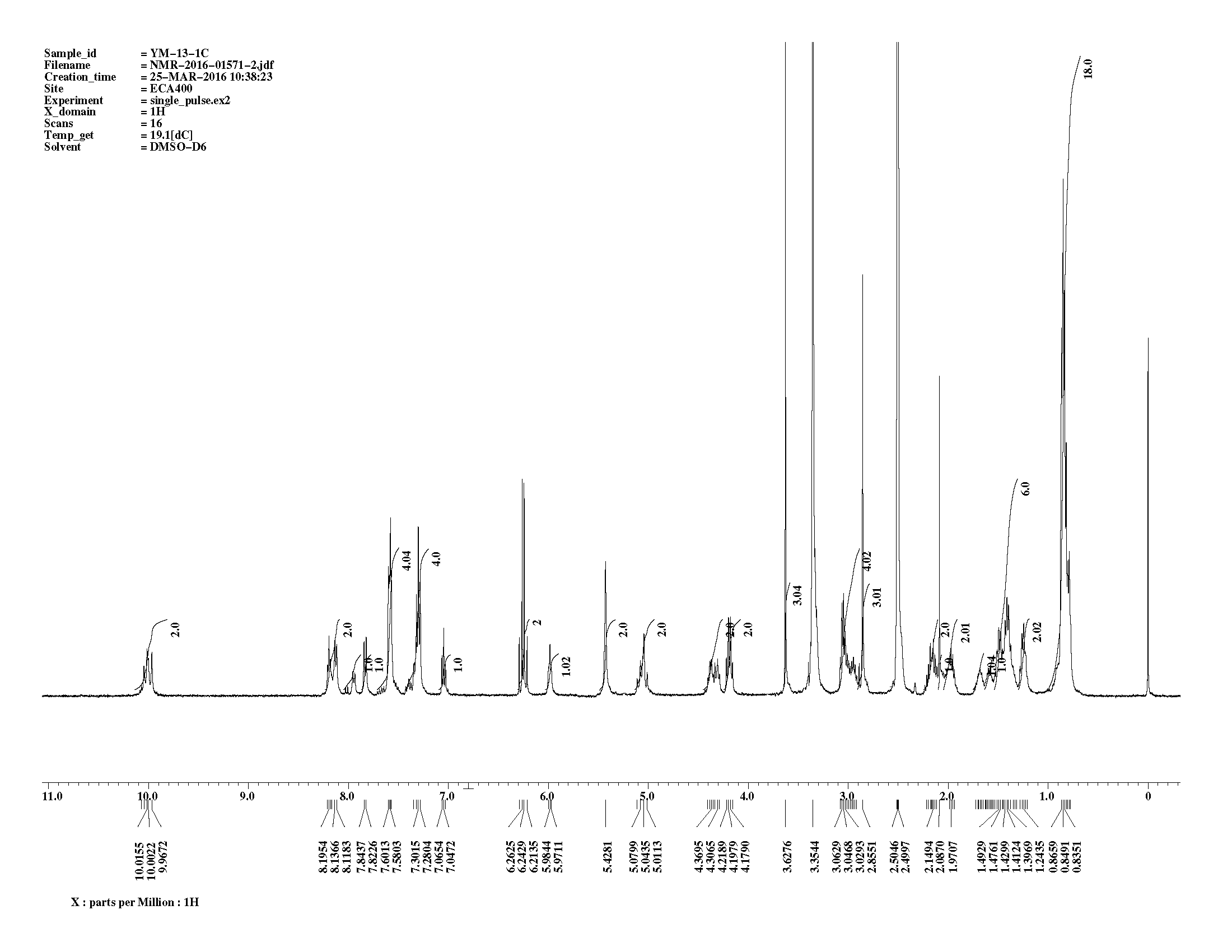


The ^1^H-NMR spectrum of Compd. **S24**





The MS spectrum of Compd. **S24**


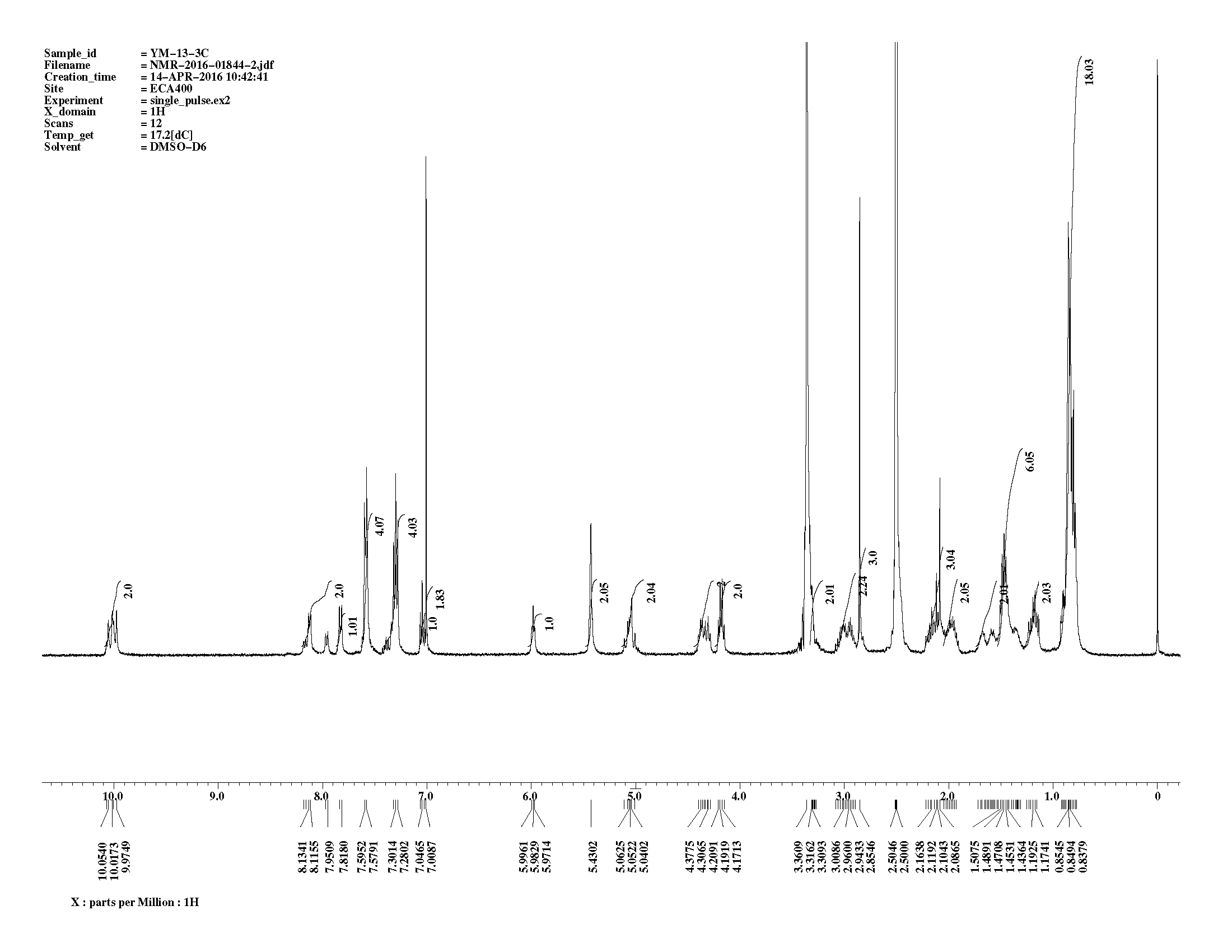


The ^1^H-NMR spectrum of Compd. **S26**


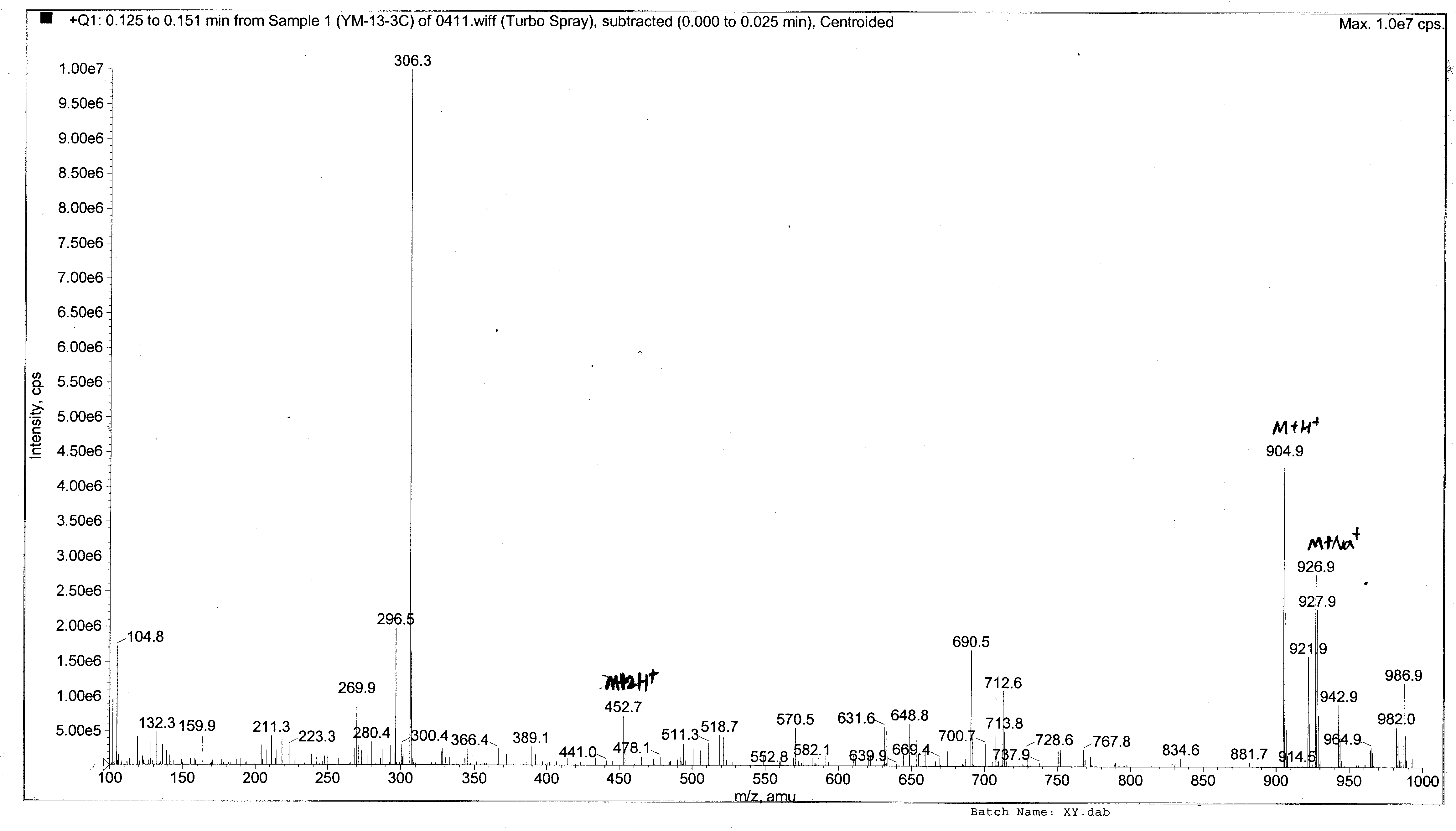


The MS spectrum of Compd. **S26**
